# Systematic Discovery of New Recognition Peptides Mediating Protein Interaction Networks

**DOI:** 10.1371/journal.pbio.0030405

**Published:** 2005-11-15

**Authors:** Victor Neduva, Rune Linding, Isabelle Su-Angrand, Alexander Stark, Federico de Masi, Toby J Gibson, Joe Lewis, Luis Serrano, Robert B Russell

**Affiliations:** **1**European Molecular Biology Laboratory, Heidelberg, Germany; **2**European Molecular Biology Laboratory–European Bioinformatics Institute, Hinxton, United Kingdom; University of MichiganUnited States of America

## Abstract

Many aspects of cell signalling, trafficking, and targeting are governed by interactions between globular protein domains and short peptide segments. These domains often bind multiple peptides that share a common sequence pattern, or “linear motif” (e.g., SH3 binding to PxxP). Many domains are known, though comparatively few linear motifs have been discovered. Their short length (three to eight residues), and the fact that they often reside in disordered regions in proteins makes them difficult to detect through sequence comparison or experiment. Nevertheless, each new motif provides critical molecular details of how interaction networks are constructed, and can explain how one protein is able to bind to very different partners. Here we show that binding motifs can be detected using data from genome-scale interaction studies, and thus avoid the normally slow discovery process. Our approach based on motif over-representation in non-homologous sequences, rediscovers known motifs and predicts dozens of others. Direct binding experiments reveal that two predicted motifs are indeed protein-binding modules: a DxxDxxxD protein phosphatase 1 binding motif with a *K*_D_ of 22 μM and a VxxxRxYS motif that binds Translin with a *K*_D_ of 43 μM. We estimate that there are dozens or even hundreds of linear motifs yet to be discovered that will give molecular insight into protein networks and greatly illuminate cellular processes.

## Abstract 2

Many protein interactions are mediated by short amino acid motifs. The authors describe a new approach to identify these interaction motifs and experimentally validate some of their binding predictions.

## Introduction

Protein interactions are central to all cellular processes. At the molecular level they can occur in a variety of ways. Probably the best known involve specific contacts between globular domains (∼100–200 residues) present in the interacting proteins. These are seen in many different contexts ranging from different subunits in large molecular machines (e.g., RNA polymerase II [1]), to more transient interactions (e.g., cyclins binding to CDK2 [2]).

However not all interactions are mediated by pairs of globular domains. Many involve the binding of a domain in one protein to short regions (approximately three to eight residues) in another [3,4]. These regions often show a particular sequence pattern, or “linear motif,” which captures the key residues involved in function or binding [5]. Linear motifs are critical to many processes including signal transduction (e.g., SH3 domains bind PxxP [6]), gene expression (e.g., Groucho→WRPW [7]) and DNA replication (e.g., PCNA→QxxxxxFF [8]).

In contrast to domains, which are readily detectable by sequence comparison, linear motifs are difficult to discover due to their short length, a tendency to reside in disordered regions in proteins, and limited conservation outside of closely related species. To date they have typically been found by time-consuming experiments, meaning that only a few hundred motifs are known compared to thousands of domains that might bind them. Although it is at present difficult to estimate just how many such interaction motifs exist, it is likely that many interactions are mediated by those not yet discovered. Here we perform the first systematic attempt to discover new motif candidates and their corresponding binding partners using results of genome-scale interaction datasets.

## Results

### Methodology

**Figure 1:**
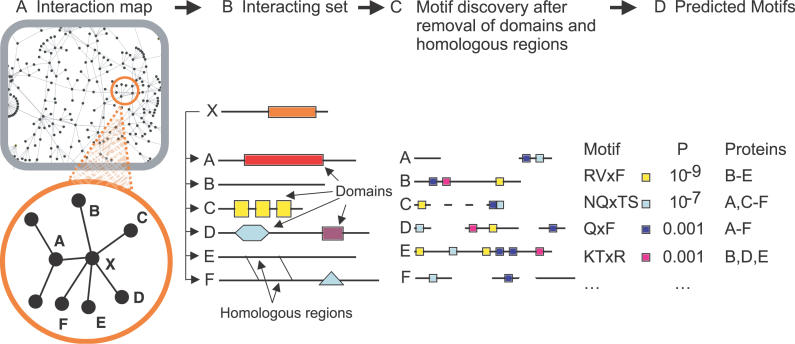
Schematic of the Linear Motif Discovery Strategy Interaction maps are probed for interaction sets (A): Partners of proteins with multiple interactions are clustered together when there are no known sequence features present (B). Domains and homologous regions are then identified (B) and removed prior to running exhaustive pattern discovery (C) to produce a list of motifs ranked by their probabilities *P* (D). Hypothetical motifs are shown as coloured squares in (C) and (D). “Proteins” in (D) gives the set of proteins containing at least one copy of the motif.

Our central hypothesis is that proteins with a common interaction partner will share a feature that mediates binding, either a domain or a linear motif. In the absence of a shared domain, a linear motif could well be the only common sequence feature and might thus be detectable simply by virtue of over-representation, which is the basis of our approach (Figure1).

Given a set of proteins sharing an interaction partner we first remove sequence regions unlikely to contain linear motifs: globular domains, trans-membrane segments, coiled-coils, collagen regions, and signal peptides. This is justified because only 15% of known linear motifs [5] occur within these regions, and including them can give rise to misleading motif signals, particularly if common domains are found in more than one protein in a set. Most importantly, this avoids the detection of repetitive, purely structural patterns, such as β-turns, coiled-coil heptads, or collagen repeats, because these are unlikely to occur in the unstructured parts of proteins that remain after this filtering. We also compare all sequences in a set to each other and leave only one representative of any homologous segments. We do this in order to measure over-representation that is not the result of homology; our assumption is that each of the remaining instances of a particular motif has arisen convergently and is thus an independent observation. We specifically avoid removing regions of low complexity because linear motifs frequently occur within them.

We then find all three to eight residue motifs in the remaining sequence [9], and score their over-representation as the binomial probability *(P)* of seeing them randomly in a similar set of sequences (see Materials and Methods). This allows multiple observations of an otherwise insignificant motif to become statistically significant by over-representation, and readily accounts for sets of different sizes and composition. For example, the SH3-binding pattern RxPxxP readily occurs in about one out of 20 randomly selected proteins, but its occurrence in seven sequences in a set of nine becomes highly significant. We also compute *P* for all closely related species based on whether or not the same motifs are seen in the corresponding orthologues, and multiply these to give a final score (*S_cons_;* see Materials and Methods).

We applied our approach to interacting sets of proteins from *Saccharomyces cerevisiae, Drosophila melanogaster, Caenorhabditis elegans,* and *Homo sapiens* [10–14]. For the first three species, these datasets are from yeast two-hybrid screens; human data comes from the human Proteome Resource Database (HPRD) [14] and consists of hand-curated interactions extracted from the literature (see Protocol S1). For each dataset, we constructed a control by selecting random sets of proteins of a similar length and number, and performed the same calculations. We then defined a confidence threshold (*p-*value < 0.001) for *S_cons_* for each dataset (see Materials and Methods). Note that this threshold does not necessarily reflect the accuracy in terms of identifying *binding* motifs, only that the particular sequence pattern reported is very unlikely to arise by chance. It is possible that patterns can arise for other reasons, including localization signals or other sequence features common to protein performing similar function.

Known motifs come in different flavours, for instance canonical SH3-binding motifs (PxxP) are embellished with different amino acids, which determine the specific SH3-containing protein they bind (e.g., RxPxxP and PxxPxK). The sets of proteins above (i.e., those sharing an interaction partner) are appropriate for finding such motif flavours because each protein containing a particular instance of a domain (e.g., SH3) is considered separately. However, it is also beneficial to detect more general motifs specific to a domain family. To do this we simply merge sets if the common binding partners shared a particular domain. We refer to these as “domain” sets in the sections that follow (see Protocol S1 and Figure S1).

### Benchmark

The Eukaryotic Linear Motif resource (ELM) [5] contains a curated set of experimentally validated instances of binding motifs (i.e., their location in a particular protein). This provides several pertinent sets of proteins to test the approach, namely each set of proteins containing a known instance of a particular motif (e.g., all PxxP motif–containing sequences known to interact with SH3 domains). Of 58 different sets, 22 contained at least four non-homologous instances of the motif, and could be used to test our approach. We ran the procedure on each set and monitored where the known motif (or a variant) was found in the list of all motifs ranked according to *S_cons_*. Despite many thousands of possibilities, the approach detected the correct motif as the very best ranked for 14 out of 22 and among the top ten for an additional three (Table 1). Applying the confidence threshold left eleven correct motifs at first rank, and no false predictions (see legend to Table 1). Inspection showed that those motifs that were either missed or scored poorly were generally highly degenerate in nature (e.g., the sumoylation site (VILAFP)Kx(EDNGP)).

**Table 1:**
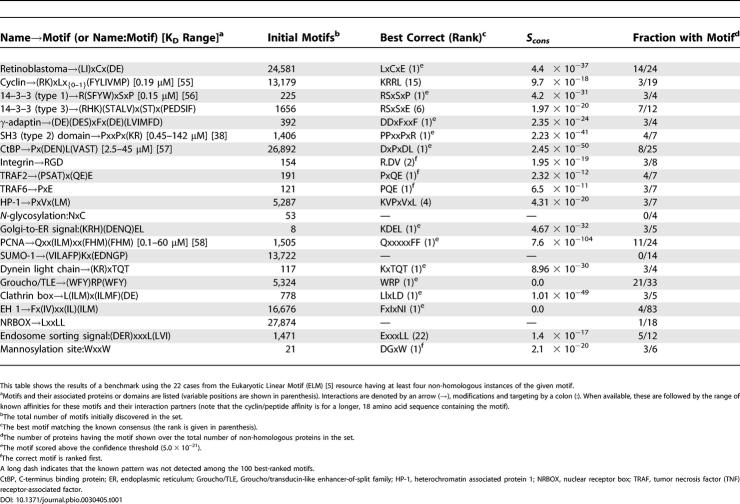
Detection of Known Linear Motifs in Experimentally Verified Sets from the Eukaryotic Linear Motif

### Motifs in Genome-Scale Interaction Sets

**Figure 2:**
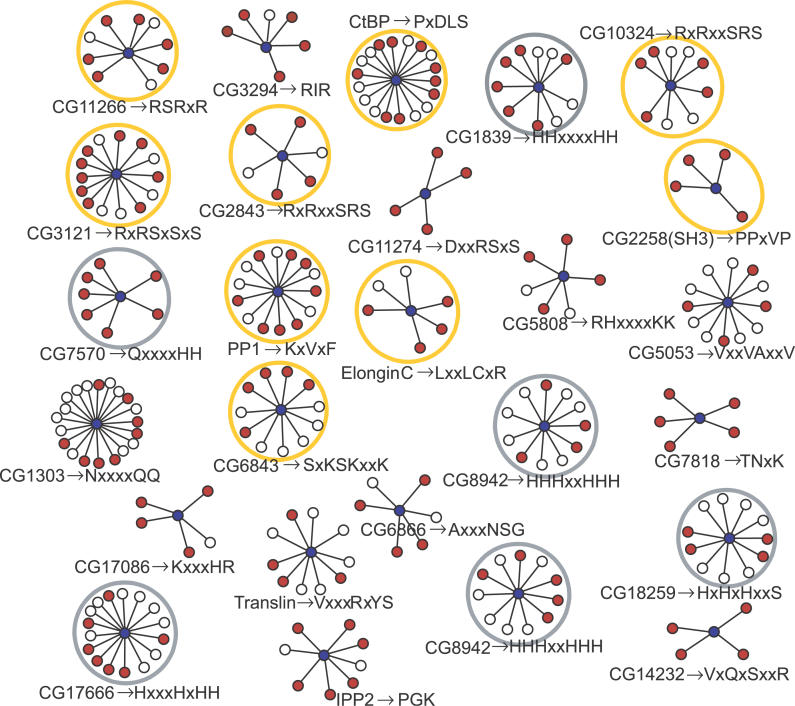
Overview of Motifs Found in the Fly Significant predictions from the yeast two-hybrid set for the fly. Blue dots in the center of each cluster represent proteins with four or more interaction partners (red and white dots) containing at least one confidently predicted motif (*p-*value < 0.001; *S_cons_* ≤ 8 × 10^−15^). Partner proteins containing the motif are represented by red dots, whereas proteins lacking the motif are indicated by white dots. Clusters are labelled as gene name→detected motif. Yellow circles enclose known motifs: SH3→PxxP [38], PP1→RVxF [22], C-terminal binding protein (CtBP)→PxDLS [52], SR splicing factors RS-rich segments [53], and CG6843→SxKSKxxK, a likely nuclear localization signal. The Translin→VxxxRxYS motif was experimentally tested (Figure 3). The grey circles enclose clusters with low-complexity patterns. Two additional known motifs were also found in the fly using more relaxed criteria than those used for the other motifs in the figure: Groucho→WRPW [7] and Dynein light chain→TQT [26] as the variant A(TI)QT(DE). The latter was also identified as significant in the domain sets. Proteins are denoted either by their FlyBase accession codes or protein names when available.

Considering the genome-scale interactions, each dataset produced a number of protein sets sharing a common interaction partner: yeast, 191; fly, 632; nematode, 367; and human, 1,986. Only a small fraction of these produced one or more confident motifs (as assessed by the binomial probability): yeast, 11; fly, 26; nematode, 27; and human, 112. In all cases, known motifs were among those produced, though to varying degrees: yeast, 1 (domain set); fly, 9; nematode, 4 (domain set); and human, 48 (all significant motifs from the protein set are given in Protocol S1 and Table S1; all motifs, including those with poorer significance, are available at http://lmd.embl.de). Figure 2 shows a summary of the 26 motifs found in the fly set, highlighting the nine rediscoveries of known motifs (including one likely nuclear localization signal). The better results in human data (i.e., 48/112) are undoubtedly because the hand-curated interactions (HPRD, [14]) contain fewer errors than those from the comparatively noisy high-throughput yeast two-hybrid screens for the other organisms. Here we found motifs spanning virtually the full range of those known (SH3→PxxP, 14–3–3 proteins→RxxSxP, Clathrin→LDxL, etc.), in addition to several that appear to be novel.

Inspection showed that known motifs were typically missed because the sets contained too few sequences with the correct motif to reach significance. For example, in yeast interaction data, just four out of 23 proteins interacting with the protein phosphatase 1 (PP1) domain contained the established (RK)VxF motif. A similar situation occurred with WW domains, where no more than three instances of known motifs were found among their interaction partners. It could be that certain motifs are just too rare in the interacting set to be detected. However, it is also well established that the yeast two-hybrid system, particularly when applied in genome-screens, can miss known interactions [15] and, moreover, make false predictions that cloud the signal from true motifs. The prediction accuracy and coverage will certainly increase when more comprehensive and reliable interaction data become available. The error prone nature of the underlying yeast two-hybrid data for yeast, fly, and nematode might be expected to yield inconsistencies (i.e., different motifs for the same protein) when comparing predictions from different species. Encouragingly, however, we found very few of these, and indeed in one case (see PP1, in Experimental Testing of New Motifs), we think the apparent inconsistency corresponds to two distinct motifs that bind to the same protein, each detected in a different species.

Many of the motifs detected in the protein sets were also found when interaction partners were pooled owing to the presence of a common domain (domain sets). Frequently a more general motif was found in the domain than in the protein sets. For example, in the fly we identified the canonical TQT motif as the binding site for Dynein light chain domains, but found the more specific pattern A(TI)QT(DE) for the specific Dynein homologue Cdlc2. Other motifs were seen only in one of the protein or domain sets. For example, in yeast a correct SH3 motif was only found in the domain sets, because no single SH3-domain protein had a sufficient number of interaction partners for the motifs to be found with significance. The reverse was true in the fly in which the correct SH3 motif was found only in the protein set, because the domain set had too many proteins lacking the canonical motif, meaning that the signal was lost.

There is also a problem of ambiguity in both protein and domain sets. For multi-domain proteins predicted to bind a motif, it is not possible to discern which domain is mediating the interaction. This can be partly resolved by considering domain sets, but even here there are still some examples where genuine motifs were predicted for the wrong domains. Inspection showed that this was either because the process selected the wrong domain of a frequently co-occurring pair (e.g., SH2 domains predicting to bind SH3 ligands) or selected an activator/inhibitor of the correct binding domain (e.g., protein phosphatase inhibitor 2 (IPP2) binding to PP1-like RVxF-like motifs [16]). The latter highlights the possibility of the yeast two-hybrid system identifying indirect interactions [17]. These domain ambiguities can likely only be resolved by experiment.

### Experimental Testing of New Motifs

For a selection of new protein→motif associations, we tested direct binding via fluorescence anisotropy, using labelled peptides corresponding to the regions containing the predicted motifs (Protocol S1). Because the motifs we have predicted might be the lowest common denominator of what could be a slightly longer binding region, we included two additional residues to the N- and C-terminus of each peptide (extracted from the original sequence). We first selected candidate motifs in yeast, fly, and nematode based on the feasibility of expressing and purifying the common interaction partner (i.e., the protein predicted to bind the motif). Of the 55 significant novel motif predictions, only 13 contained a single globular domain, were not excessively long (≤ 650 amino acids), and lacked long regions of predicted disorder or low complexity. From these we selected five that had available clones and established purification protocols. These spanned a range of novelty, ranging from variations on a known motif, to those for which there was some supporting, but not direct, evidence in the literature, to those lacking any additional support. Of the five selected, we could obtain clones for four and could purify three.

**Figure 3:**
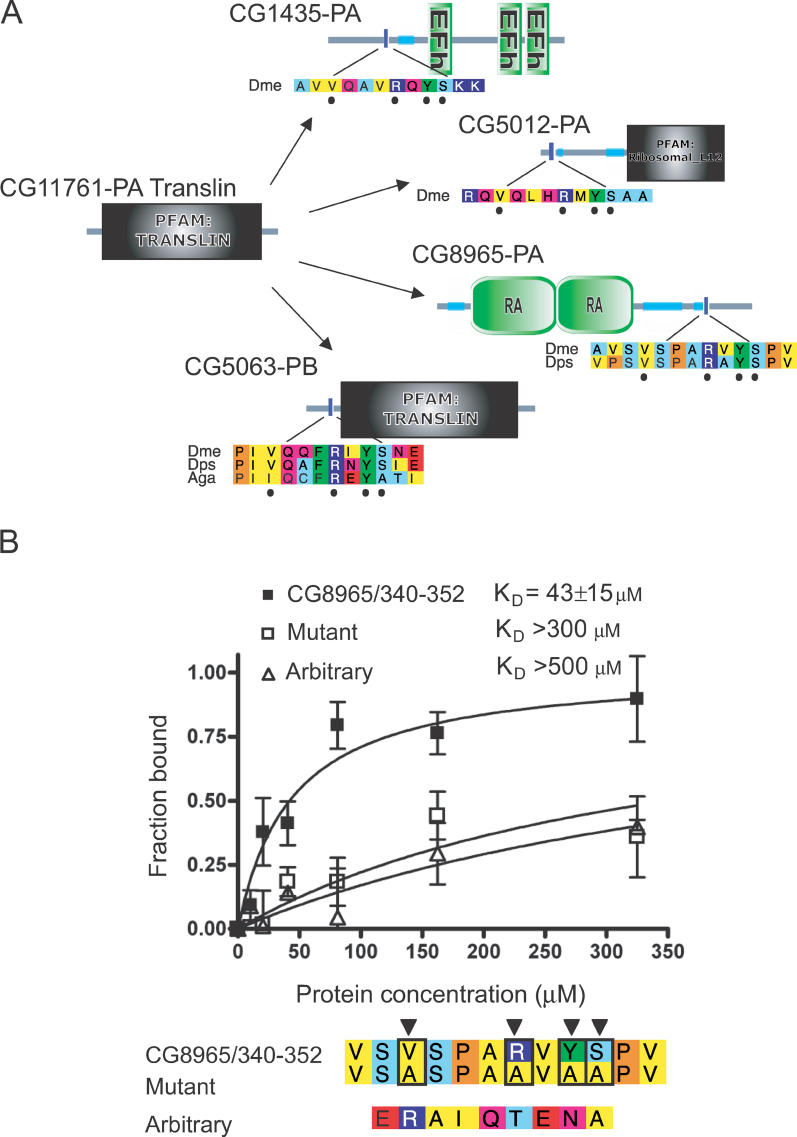
A Novel Fly VxxxRxYS Motif That Binds Translin (A) Translin (left) shown surrounded by interaction partners containing the predicted motif VxxxRxYS. Proteins are shown as lines with domains (labelled shapes), predicted coiled coils (light blue/green segments), and the location of motifs (blue vertical bars). Sequences for the motif-containing region are shown aligned to the best homologues in closely related species. Amino acids are coloured according to residue type: blue, positive; red, negative; light blue, small; yellow, hydrophobic; green, aromatic; magenta, polar; and orange, proline. Those constituting the predicted motif are denoted by circles. Aga, *Anopheles gambiae;* Dme, *D. melanogaster;* Dps, *D. pseudoobscura*. (B) Saturation curves, showing bound fraction (fluorescently labelled peptides at saturation) as a function of Translin concentration. Polarization values (mP) at zero concentration and B_max_ were normalised to give the bound fraction. *K*_D_ was computed by non-linear regression on values from three independent experiments. The lower panel shows the alignment of the native and mutated peptides together with the arbitrary peptide (selected randomly). Black triangles show positions specifying the motif (VxxxRxYS). The alignment is coloured as described in (A).

We tested a highly significant Translin→VxxxRxYS motif found in fly data (Figure 3A). Translin is a protein thought to be involved in chromosomal rearrangements, and binds double-stranded RNA and DNA [18,19]. The fluorescence polarisation assay shows that it binds the peptide motif specifically compared to a mutated counterpart, or randomly selected peptides (Figure 3B). The affinity of binding (*K*_D_ = 43 ± 15 μM) is within the range typical for known linear motifs when considered in isolation (5–150 μM; see Table 1). Mutated controls or arbitrarily chosen peptides do not show specific binding (Figure 3B; note that the apparent linear increase in both is due to the high protein concentrations reached). We can only speculate what role this motif plays in modulating Translin function. However, there are several precedents for interaction motifs playing critical regulatory roles by binding to other DNA- or RNA-binding proteins, such as PCNA [20] or CtBP [21].

**Figure 4:**
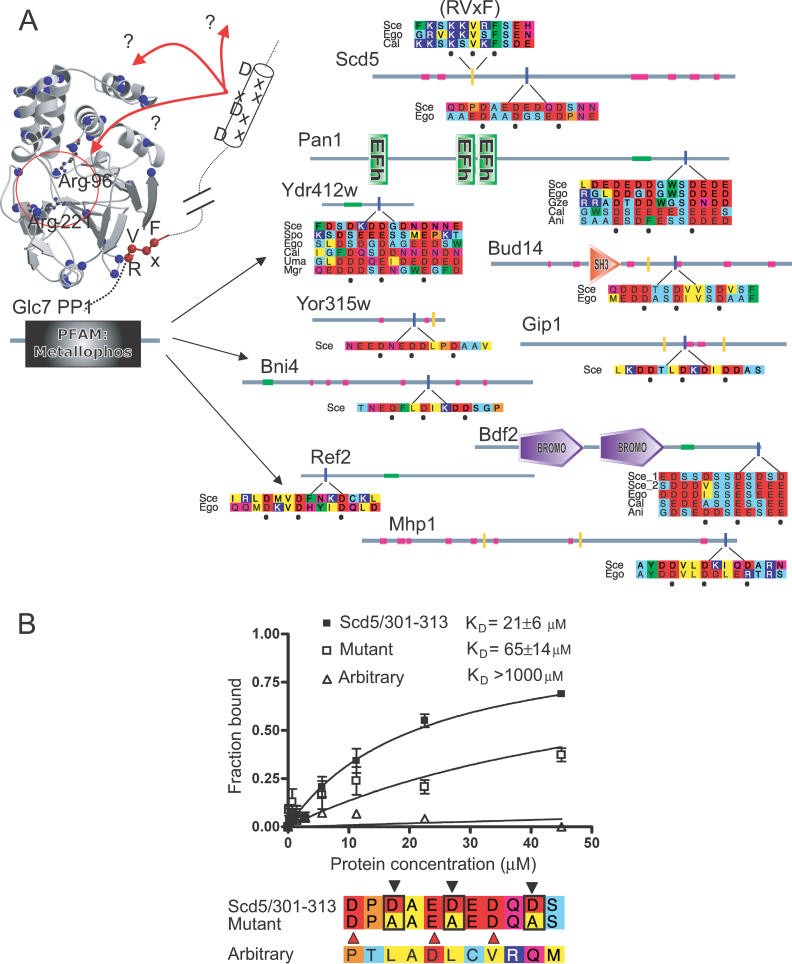
An Acidic Yeast PP1 Binding Motif (A) PP1 (Glc7) with the set of interaction partners containing the DxxDxxxD motifs. Details are as for Figure 3A. Here the location of RVxF motifs (defined as matches to (RK)x_0–1_(VI)x(FW)) are shown as yellow bars, and low-complexity regions are magenta. The figure also shows the structure of PP1 bound to RVxF (red spheres) [54] with a hypothetical helix containing the motif. Blue spheres show the location of Arg or Lys residues, and the active site is circled with critical Arginines shown in ball-and-stick. Red arrows show hypothetical interactions of the motif either with sites on PP1 or elsewhere. Ani, Aspergillus nidulans; Cal, Candida albicans; Ego, Eremothecium gossypii; Gze, Gibberella zeae; Mgr, Magnaporthe grisea; Sce, S. cerevisiae; Spo, Schizosaccharomyces pombe; Str, Salinospora tropicalis; Uma, Ustilago maydis; Xla, Xenopus laevis. (B) Saturation curves, showing bound fraction as a function of PP1 concentration. The polarization values (mP) were normalized to an extrapolated B_max_ because B_max_ could not be reached experimentally. Other details are as given in Figure 3B. Red triangles in the lower panel show the location of the near match to the motif in the mutated sequence.

We also tested a DxxDxxxD motif found in 10 of 12 interaction partners of yeast protein phosphatase 1 (PP1, Figure 4A). Eight are well-known PP1 interactors, and five contain the canonical RVxF PP1-binding motif. Fluorescence polarisation shows that a peptide corresponding to the region in Scd5 binds specifically to PP1 (*K*_D_ = 22 ± 5 μM), compared to arbitrary peptides (Figure 4B). Inspection of other PP1-binding proteins [22] reveals that 12 of 33 also contain the new motif, with an additional 15 containing a more relaxed pattern (permitting Glu). Deletions of the canonical RVxF motif do not always disrupt PP1-binding, and have led others to suggest additional binding sites [23]. Interestingly, deletions of some segments containing this new motif can affect PP1 binding in other proteins [22]. Other support comes from pull-down studies, which identified a similar region (RVRLDDDDE) critical for the Cdk5–PP1 interaction [24] and the recent crystal structure of human PP1 bound to a myosin-targeting subunit MYPT1, which led the authors to propose a positively charged surface on which a similar acidic stretch could interact [25]. Interestingly, the mutated control appears to retain some affinity, probably owing to the presence of additional negative charges that have not been mutated to alanine, and indeed a near match to the motif (DxxxExxD) is still present in the mutant. Arbitrary peptides did not show any specific binding (Figure 4B).

Lastly, we tested a variant of the well-known Dynein light chain binding motif. The canonical motif has a consensus sequence (KR)xTQT and mediates interactions important for cell trafficking [26]**.** We found the canonical motif in the fly, but noticed a variant, IQTE, among three partners of Cdlc2 (Dynein light chain 2), which is similar to one present in the protein swallow from *D. pseudoobscura* [27]. We could detect no binding of the Cdlc2 to fluorescently labelled peptides over a range of protein concentrations (5–400 μM). Surprisingly, a true instance of the motif, known to bind Cdlc2 in vivo and in vitro [27], also did not give a signal using this procedure, suggesting that the experimental assay might not be suitable for Dynein light chain interactions (see Protocol S1).

### Other Promising Predicted Motifs

For other predictions, we scrutinized the literature for previous experiments hinting that the motifs could be genuine. For example, among several interesting predictions in the fly was an Elongin C→LxxLCxR motif, which has been described previously only as part of a longer sequence called the SOCS box [28]. Only three of four interacting proteins with the motif contain the full SOCS box. The protein lacking it (CG18171) is not well understood; the interaction has not been reported apart from the genome screen. Deletion and mutagenesis experiments have shown that this region is important for the interaction with Elongin C [29]. Our finding agrees with this and further suggests that the motif could be sufficient on its own for mediating the interaction.

**Figure 5:**
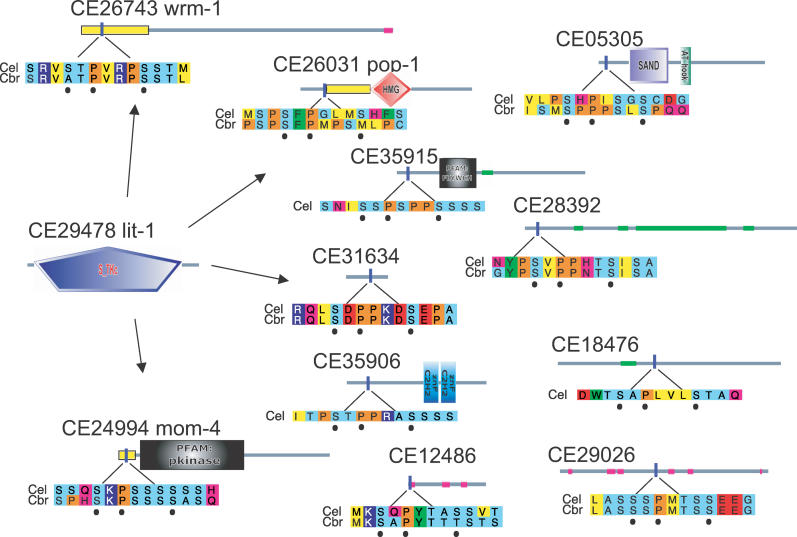
A Lit-1 MAP Kinase SxPxxxS Motif The MAP kinase lit-1 surrounded by its interaction partners containing the SxPxxxS motif. Details are as for Figure 3. Yellow boxes show the location of deletion mutants known to affect the interaction. Cbr, *C. briggsae;* Cel, *C. elegans*.

We found the motif SxPxxxS in 11 of 17 interaction partners of the nematode MAP-kinase lit-1 involved in *wnt* signalling and morphogenesis (Figure 5). These include three well-known regulators/interactors of lit-1: two nuclear proteins (wrm-1 and mom-4) and another morphogenesis protein (pop-1). Deletions have already demonstrated that regions containing the motif are critical for lit-1 binding (yellow boxes in Figure 5): a 148 N-terminal segment in wrm-1 [30], a 21-residue stretch in mom-4 [31], and a 45-residue region just six residues N-terminal to the motif in pop-1 [32] all disrupt lit-1 binding.

Among several compelling new motifs in human was a T(PL)QP motif predicted to bind to the transcription factor PC4 (Positive Cofactor 4). PC4 binds double-stranded DNA and promotes the assembly of the preinitiation complex via a mechanism that is not fully understood [33,34]. The five proteins containing the putative motif all participate in transcription, but share no common globular domain that could mediate binding to PC4. Such a proline-rich motif could be a good candidate to bind one of the several aromatic patches on the surface of the PC4 protein [35].

### Low-Complexity Linear Motifs

In both fly and nematode, several very significant motifs arose from regions of low sequence complexity (i.e., dominated by a few amino acids). These included examples already known to mediate interactions, and others not described previously, including His-, Ser-, Lys-, and Glu/His-rich motifs. We could find no motifs like these in random sets, which suggested that they are not the result of the general prevalence of low-complexity regions within proteins, but just what they mean is an open question. They might well be true, biologically meaningful interactions, and indeed for some sets the proteins show similarities in function. This idea is supported by the fact that many known motifs, including the protein/RNA binding RS/SR motifs [36], the Tudor domain→(RG)_n_ [37], and SH3→poly-proline ligands [38], are themselves low complexity. Alternatively, they could be the result of some artefact of the yeast two-hybrid system. The last possibility is supported by the fact that we found fewer such motifs in the less error-prone human data.

### How Many Protein–Motif Interaction Pairs Are Still to Be Found?

Both our experiments and those done previously suggest that many of our findings are genuine motifs that have not yet been reported. This raises the question as to how many new interaction motifs there are yet to be discovered. An estimate can come by considering what fraction of the previously known motifs we found and extrapolating this to the new discoveries. For example, in fly we predict 26 motifs of which nine are known, from a total number of roughly 60 that are known in this organism [5]. If we assume that *all* the remaining motifs are correct, and assume an equal distribution of motifs in fly proteins not seen in the yeast two-hybrid data (4,683/13,833), we estimate 334 additional motifs (the equivalent number for human is 405). Even the more modest assumption of between 10%–20% of the predicted motifs being correct (roughly the fraction for which we could see direct binding experimentally, which is clearly a lower estimate) gives estimates of 33–67 new motifs in fly (40–80 in human). There are very likely dozens to hundreds of new motifs to be discovered, which will correspond potentially to thousands of individual binding sites. To date we have just scratched the surface of what is likely a sophisticated network of peptide-mediated interactions in the cell.

## Discussion

Many studies continue to highlight the importance of networks mediated by linear motifs [39,40], and each new discovery opens new lines of research into critical aspects of cell function [41]. We have shown here that these very simple features can be detected successfully, even in error-prone data, provided they occur with a sufficient frequency in otherwise unrelated proteins. The approach need not be restricted to protein-protein interactions. It can also be applied in other contexts: Any set of proteins or nucleic acids can be probed for short sequences responsible for a common biological feature (cellular location, modifications, etc.).

Both globular domains and linear motifs are modular in the sense that they are reused in different functional contexts, but they probably differ in how they arise. Domain shuffling involves duplication of part of a gene and its insertion into another. In contrast, the short length of linear motifs makes them likely to arise convergently in proteins by evolutionary drift [42]**.** This suggests that there are probably many near matches to the motifs just waiting for an appropriate point mutation to induce a function. They are, in effect, powerful switches for nature to explore during the evolution of complex functions. In this regard they are highly similar to transcription factor–binding sites [43,44] or microRNA target sequences [45]. In all three cases, molecular recognition is mediated by very short and fast-evolving sequences that are relatively unspecific in isolation, with more than one often being required for function. Identifying the correct sequence is a true needle-in-a-haystack problem, for nature and computational techniques alike.

New motifs are a treasure trove for investigations to deduce the molecular details of protein–protein interactions, particularly to understand those not mediated by domains alone. Given the essential regulatory functions of the motifs already known, we expect our new discoveries to have a profound impact on understanding the complex network of macromolecular interactions that exists in all living cells.

## Materials and Methods

For proteins in all sets, we identified domains using SMART [46], including domains from Pfam-A [47]. We also removed regions showing similarity between members in a set of sequences using BLAST (*E* ≤ 0.001) [48], which removes the redundant measurements. We used TEIRESIAS [9] to detect all non-overlapping motifs of three to eight residues, requiring at least two identical positions. The method essentially detects all motifs of a variable length (i.e., three to eight) in which positions can either be specified as a particular amino acid, or represented by a wildcard (i.e., “x”). We did not allow for conservative substitutions (e.g., D/E), and ignored any motif that occurred in fewer than three sequences in the set.

We assessed the significance of a particular motif occurring a certain number of times within a set of sequences (interaction set) using the binomial distribution:



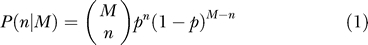



where *p* is the probability of seeing the motif in a background database, *n* is how often the motif was seen in the set of proteins, and *M* the size of the set.

The probability *(p)* was computed as a frequency of the motif in the background database of 15,000 randomly selected proteins. These proteins were taken from the SWISSPROT [49] and were subjected to the same filtering procedure as the test protein sets.

Values agree well with intuition: Motifs that are complex and thus rare need only be observed a few times to be significant, for example, the motif PxVPLR occurring in four out of 21 proteins gives a probability of 10^−11^. More common motifs must be seen more often to reach the same significance; for example, the VxxR (a subset of the first motif) must be seen in 19 out of 21 to reach a similar probability.

True instances of linear motifs are typically conserved across closely related species [42]. It is thus an advantage to use the information from the same (i.e., orthologous) protein in multiple genomes. Information from orthologues can be readily combined into a single value *(S_cons_)*, which is the product of all binomial probabilities from the genomes considered:



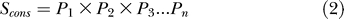



This procedure will decrease the final value (and thus increase the significance) for all conserved motifs, but will have no effect if the motifs (or indeed the orthologues) are missing. The combined value is no longer a true probability, because the motifs from related species are not independent, but rather are a measure of likelihood of a conserved motif to occur at random in the set. To estimate significance we thus compare the values to those generated from random sets of proteins. These combined values greatly improve the sensitivity and specificity of the procedure: More known motifs are recovered and fewer clearly false predictions are made.

To get confidence thresholds for *S_cons_,* we created 50 random sets of sequences of the same number and length as seen in the interaction sets for each organism using the complete proteomes. We then ran the complete procedure for each random set and computed the distribution of *S_cons_,* which gave thresholds (*p-*value < 0.001) for each dataset: 3.0 × 10^−17^ for yeast, 7.5 × 10^−14^ for nematode, 8.0 × 10^−15^ for fly, and 7.0 × 10^−38^ for human. The differences between the thresholds are due largely to differences in the number and similarity of closely related species with complete genomes available: Four substantially similar genomes were available for human but only one for the fly and nematode.

We extracted orthologues from the STRING database [50] and aligned those using MUSCLE [51] with default parameters. We considered only closely related species because known instances of linear motifs are rarely conserved outside of them. We considered orthologues in the four other completely sequenced yeast genomes *(Kluyveromyces lactis, Ashbya gossypii, Debaryomyces hansenii,* and *Candida glabrata)* for yeast *(S. cerevisiae)* motifs, *D. pseudoobscura* for fly *(D. melanogaster), C. briggsae* for nematode *(C. elegans),* and *Mus musculus, Rattus norvegicus, Gallus gallus,* and *Fugu rubripes* for motifs found in human *(H. sapiens)* proteins.

The Linear Motif Discovery (LMD) program and all data related to this paper are available online (http://lmd.embl.de).

## Supporting Information

Protocol S1 Supplementary Information (289 KB PDF).

Figure S1 Schematic of Discovery Process of Linear Motifs Recognized by Protein Domains (77 KB PDF).

Table S1 All Significant Motifs from the Protein Sets and the Rediscovered Motif:Domain Sssociations for Yeast, Fly, Nematode, and Human Interaction Datasets (173 KB PDF).

## Abbreviations

PP1: protein phosphatase 1
*S_cons_*: the product of all binomial probabilities for sets of orthologous proteins from a given set of genomes
