# Supplementary material for: Systematic Discovery of New Recognition Peptides Mediating Protein Interaction Networks
Source: PLoS Biol. 2005 Nov 15;3(12):e405. doi: 10.1371/journal.pbio.0030405 (PMC1283537; doi:10.1371/journal.pbio.0030405)
Supplement: Supplementary file 1 [file pbio.0030405.sd001.pdf]

Systematic discovery of new recognition peptides mediating protein interaction networks

*Victor Neduva(1), Rune Linding(1), Isabelle Su-Angrand (1), Alexander Stark(1), Federico de Masi (1), Toby J. Gibson(1), Joe Lewis (1), Luis Serrano (1) and Robert B. Russell(1,2)**

1.EMBL, Meyerhofstrasse 1, 69117 Heidelberg, Germany

2.EMBL-EBI, Wellcome Trust Genome Campus, Hinxton, CB10 1SD, UK

*Corresponding author:

Tel: +49 6221 387 473; FAX: +49 6221 387 517; Email: russell@embl.de

Keywords: Cell signalling, peptide, protein interaction, Protein phosphatase 1, Translin

Supplementary Information

Interaction data

We searched for new motifs among sets of proteins sharing an interaction partner. We used interactions determined by genome-scale yeast two-hybrid screens or mined from the literature to create sets of four or more proteins sharing an interaction partner. For Yeast (*S. cerevisiae*) we used all data from Uetz et al. [1] and core-data from Ito et al. [2]. For Fly (*D. melanogaster*) we used those with a confidence ≥0.5 from Giot et al. [3], and for Nematode (*C.* elegans) we used the core 1, core 2 and literature data from Li et al. [4]. For Human (*H. sapiens*) we used interaction data extracted from the literature in the Human Proteome Resource Database (HPRD [5]). We did not use data from affinity purifications as they capture fewer direct, transient interactions [6] typical of those involving linear motifs. To find motifs of a more general nature (i.e. core motifs typical for a domain family), we pooled proteins into 'domain' sets (as distinct from 'protein' sets) if their interaction partners shared a common domain (Fig S1).

Domain versus protein sets

We constructed domain sets by pooling the all interaction partners for proteins containing a particular domain (Fig S1). As shown in the Figure S1, this pooling can

sometimes lead to the detection of motifs that might otherwise be missed because of too few interaction partners when considering single proteins. However, this can also lead to motifs being assigned to the wrong domain (e.g. SH3 PxxP motifs can be assigned to SH2 domains since SH3 & SH2 domains often co-occur).

Recombinant DNA constructs

We cloned Dynein light chain (Cdlc2, 10 kDa) and Translin (Translin, 27 kDa) from *Drosophila melanogaster* directly into pET41b+ vector (Novagen); PP1 from *H. sapiens* (PP1, 37 kDa; N.B. the human and yeast proteins are 93% identical), kindly provided by Mathieu Böllen (Katholieke Universiteit Leuven), in a pTacTac vector [7]; and Elongin C from *H. sapiens* (EloC, 15 kDa; N.B. the human and fly proteins are 94% identical), kindly provided by Joan Conaway (Stowers Institute, Kansas City), in a pRSETB vector (Invitrogen).

Protein Expression

We used positive clones to transform *E. coli* BL21(DE3) (Stratagene), and grew cells at 37 ºC in 6 x 1l of Luria-Bertani broth supplemented with either 50 μg/ml Ampicillin (PP1 and EloC) or 30 μg/ml (Cdlc2 and Translin) until absorbance at 600 nm reached 0.4. We then induced over-expression by the addition of 40 μg/ml Isopropyl-ß-D-thiogalactopyranosid (IPTG) and grew cells for 16h at 16ºC.

We harvested the cells and re-suspended them in 80 ml PBS together with one Complete™ protease inhibitor cocktail tablet (Roche) and DNAseA. We then lysed them using a French Press (Emulsiflex C5) at 1000-1500psi. We cleared the lysate by centrifugation at 40000 rpm for 50 min, and loaded the soluble fraction onto various purification columns depending on the construct.

Protein Purification

We purified all proteins using a Biologic HR workstation (Bio-Rad). For Cdlc2 and Translin we used a 5ml GST-Trap column (Pharmacia) equilibrated in PBS and eluted in 50 mM Tris-Hcl pH 8.0, 10 mM Glutathione; we purified PP1 as descried previously [8]; and we used a MonoQ HR 16/10 (Pharmacia) to purify EloC. We also used a previous preparation of human *abl*-SH3 domain [9]. We were unable to purify Elongin C to the concentration required for fluorescence polarization measurements.

Fluorescence Polarization

We obtained FITC N-terminal labeled micro-scale peptides (JBT Peptide Technologies, Berlin) for fluorescence polarization measurements. A preliminary test with *abl*-SH3 binding peptide (GAPTYSPPPPP) demonstrated that we could both observe binding and compute a K_D of 3.0±0.4 μM roughly comparable to that obtained previously by measuring fluorescence of tryptophans in the *abl*-SH3 domain (0.4μM for a peptide lacking the N-terminal Glycine) [10].

For each protein/motif pair we selected peptides from two proteins containing the predicted motif, with an additional two amino acids from the N- and C- termini. For one of these we also obtained a mutant peptide where the positions specifying the motif had been mutated to Alanine. The peptides were as follows: Cdlc2 native ERAIQTENA (CG33213/104-112), mutant ERAAAAENA; PP1 native DPDAEDEDQDS (Scd5/302-312) mutant DPAAEAEDQAS; Translin VSVSPARVYSPV (CG8965/341-352), mutant VSASPAAVAAPV. When binding was observed (PP1, Translin), we tested whether the binding was non-specific by assaying a selection of other peptides arbitrarily chosen from other protein/motif pairs (an example is shown in Fig 3B & Fig 4B; none of the arbitrary peptides bound specifically). For this protein we also tried an additional peptide containing the canonical TQT motif (PFGTQTSPP, from CG32130/215-223). No binding was observed with any of the peptides for Cdlc2, even at protein concentrations as high as 400 μM.

We suspended peptides to working concentrations (10 nM final concentration) in PBS, pH 7.4. We then added increasing concentrations of the recombinant protein to PBS solution and mixed with the FITC-labeled peptides on 384-well Proxiplate (PerkinElmer, USA), and incubated each sample for 5 min at room temperature. We then measured Fluorescence polarization on an EnVision HTS (PerkinElmer, USA) according to the manufacturers' instructions. No increase in the fluorescence polarization was observed upon longer incubations. We plotted saturation binding curves with the PRISM software (Graph-Pad, San Diego, USA), and used it to compute dissociation constants (K_D) via non-linear regression using the results from three individual experiments.

References

1. Uetz, P., et al., *A comprehensive analysis of protein-protein interactions in Saccharomyces cerevisiae.* Nature, 2000. 403(6770): p. 623-7.
2. Ito, T., et al., *A comprehensive two-hybrid analysis to explore the yeast protein interactome.* Proc Natl Acad Sci U S A, 2001. 98(8): p. 4569-74.
3. Giot, L., et al., *A protein interaction map of Drosophila melanogaster.* Science, 2003. 302(5651): p. 1727-36.
4. Li, S., et al., *A map of the interactome network of the metazoan C. elegans.* Science, 2004. 303(5657): p. 540-3.
5. Peri, S., et al., *Development of human protein reference database as an initial platform for approaching systems biology in humans.* Genome Res, 2003. 13(10): p. 2363-71.
6. Aloy, P. and R.B. Russell, *The third dimension for protein interactions and complexes.* Trends Biochem Sci, 2002. 27(12): p. 633-8.
7. Muchmore, D.C., et al., *Expression and nitrogen-15 labeling of proteins for proton and nitrogen-15 nuclear magnetic resonance.* Methods Enzymol, 1989. 177: p. 44-73.
8. Barford, D., et al., *Purification and crystallization of the catalytic domain of human protein tyrosine phosphatase 1B expressed in Escherichia coli.* J Mol Biol, 1994. 239(5): p. 726-30.
9. Pisabarro, M.T., L. Serrano, and M. Wilmanns, *Crystal structure of the abl-SH3 domain complexed with a designed high-affinity peptide ligand: implications for SH3-ligand interactions.* J Mol Biol, 1998. 281(3): p. 513-21.
10. Pisabarro, M.T. and L. Serrano, *Rational design of specific high-affinity peptide ligands for the Abl-SH3 domain.* Biochemistry, 1996. 35(33): p. 10634-40.
