# Supplementary material for: Systematic Discovery of New Recognition Peptides Mediating Protein Interaction Networks
Source: PLoS Biol. 2005 Nov 15;3(12):e405. doi: 10.1371/journal.pbio.0030405 (PMC1283537; doi:10.1371/journal.pbio.0030405)
Supplement: Supplementary file 2 [file pbio.0030405.sg001.pdf]

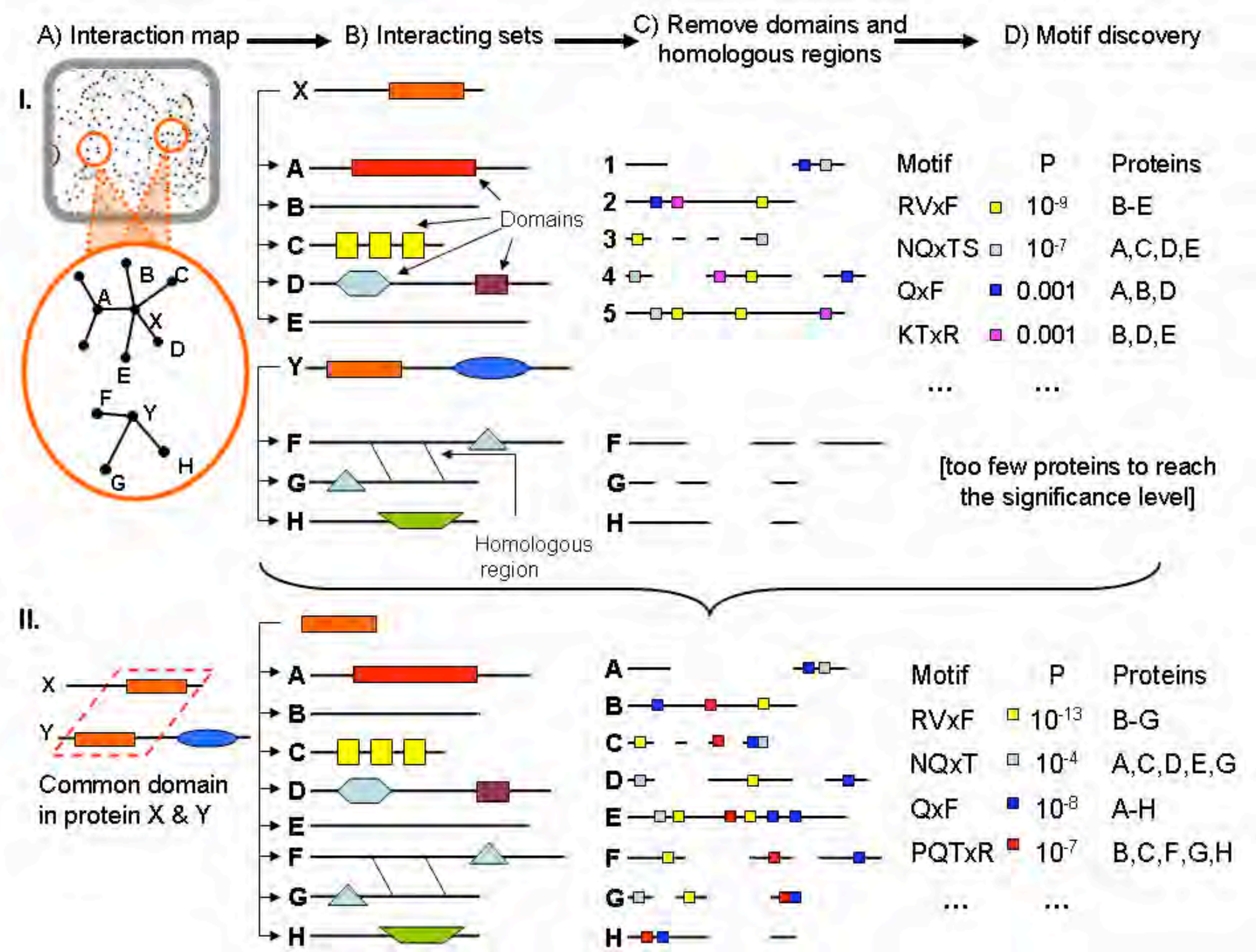

Figure S1

Interaction maps are probed for protein interaction sets (***A***): partners of proteins with multiple interactions are clustered together where there are no known sequence feature present (***B***). Domains and homologous regions are then identified (***B***) and removed prior to running exhaustive pattern discovery (***C***) to produce list of motifs ranked by their probabilities P (***D***). Hypothetical motifs are shown as coloured squares in **C** & **D**. 'Proteins' in ***D*** gives the set of proteins containing at least one copy of the motif.

I. Shows two separate cases of protein sets containing interactions partners for proteins X and Y that are pooled owing to the presence of a common domain to give a domain set shown in (**II**).
