# Supplementary material for: Systematic Discovery of New Recognition Peptides Mediating Protein Interaction Networks
Source: PLoS Biol. 2005 Nov 15;3(12):e405. doi: 10.1371/journal.pbio.0030405 (PMC1283537; doi:10.1371/journal.pbio.0030405)
Supplement: Supplementary file 3 [file pbio.0030405.st001.pdf]

Table S1

The table is divided into five sections. First four dedicated to motifs discovered from sets of proteins sharing a single interactions partner (‘proteins’ set) for each of the organisms: Fly, Yeast, Nematode and Human. For each organism it shows four motifs within confidence threshold (p-value <0.0001). The accession of the shared interaction partner and its domain composition are shaded grey. The last section devoted for the domain sets (created after combining sets of proteins interacting with a common domain) for all species. We show only the correct motif:domain association which we discuss in the text; here the grey shading highlights only the name of the domain shared by the interaction partners.

For each motif we report the combined likelihood (S_{cons}), the total number of proteins containing the motif (n) and the total number in the set (M). Proteins are given in the right-most column and are post-fixed with the sequence region containing the motif. Coloring of the motifs: magenta coloring indicate previously known motifs; yellow denote the motifs which were experimentally tested in this study; green known motifs below 99.9% confidence threshold. An asterix (*) indicate protein/domains discussed in the text. Within the motifs, an 'x' denotes positions occupied by any amino acid.

Further motifs are available at: http://lmd.embl.de

>Protein and putative binding motifs ('Protein' sets)	S_{cons}	Motif count	Total	Proteins with corresponding motif: identifiers/coordinates(first match)
**I. *S.cerevisiae* single proteins set**				
>YDL239C COIL				
PxxxRxLS	7.34E-18	4	16	YAL028W/111-119 YDR176W/399-407 YLR098C/207-215 YPL070W/102-110
>YDL065C Pfam:Pex19				
TxxLF	1.78E-19	4	5	YGR119C/129-134 YGR218W/1046-1051 YLR071C/927-932 YMR047C/202-207
LxxQQ	6.69E-19	4	5	YGR119C/103-108 YGR218W/635-640 YMR047C/276-281 YMR163C/228-233
>YDR328C Skp1				
PxxxIxY	5.02E-20	4	14	YBR202W/127-134 YLR097C/219-226 YLR368W/148-155 YMR094W/296-303
GxxxYxxL	2.92E-17	4	14	YDL132W/82-90 YLR097C/299-307 YLR352W/797-805 YMR094W/279-287
>YER133W PP2Ac*				
DxxDxxxD	2.61E-17	10	12	YAR014C/444-452 YBR045C/356-364 YDL070W/630-638 YDR195W/25-33 YDR412W/122-130 YIR006C/1274-1282 YJL042W/359-367 YNL233W/585-593 YOR315W/298-306 YOR329C/303-311
>YGR120C				
YLxxLxxL	3.93E-18	4	10	YER157W/594-602 YOR353C/716-724 YPR105C/601-609 YPR185W/154-162
>YLR423C Pfam:APG17				
KASxxxQ	1.73E-19	4	35	YGR113W/165-172 YDR052C/493-500 YDR228C/136-143 YMR124W/135-142
LxxLxK	4.38E-19	17	35	YBR270C/298-304 YDL239C/4-10 YDR022C/174-180 YDR052C/217-223 YDR176W/592-598 YDR228C/85-91 YDR473C/149-155 YER127W/3-9 YGL212W/200-206 YGL127C/102-108 YGR120C/116-122 YHL004W/69-75 YHR197W/272-278 YJL061W/323-329 YJL036W/264-270 YKR083C/14-20 YLL049W/168-174 YLR423C/202-208 YOR158W/139-145 YKL002W/224-230
LxDLxK	8.32E-18	6	35	YBR270C/298-304 YDR022C/174-180 YDR176W/592-598 YDR228C/616-622 YDR473C/149-155 YJL036W/264-270 YLR423C/202-208
>YMR308C				
RIxK	1.01E-17	4	9	YGR119C/396-400 YKL135C/6-10 YLL026W/842-846 YPL133C/292-296
>YNL189W Pfam:IBB ARM				
NxVxR	4.29E-25	5	50	YAL054C/135-140 YDR383C/49-54 YGR010W/367-372 YHR216W/306-311 YJR056C/37-42 YLR328W/373-378 YLR377C/159-164 YML042W/555-560 YPL088W/132-137
AxYN	4.26E-19	4	50	YAL054C/132-136 YDR383C/208-212 YGR010W/39-43 YHR216W/349-353
VKxxxW	1.18E-18	4	50	YBR176W/296-302 YGR144W/29-35 YGR192C/305-311 YKL130C/151-157 YMR239C/192-198
>YOL149W Pfam:DCP1				
QxxxQQ	7.56E-18	7	11	YDL160C/434-440 YDR206W/850-856 YEL015W/70-76 YGR218W/279-285 YMR047C/26-32 YNL118C/312-318 YPL204W/394-400
>YOR047C				
LPSN	2.70E-18	4	14	YHR158C/618-622 YLR305C/1589-1593 YMR280C/1112-1116 YNL201C/154-158
>YOR355W				
PNxxN	1.06E-18	7	11	YDR207C/467-472 YGL150C/1095-1100 YJL092W/552-557 YLR305C/28-33 YNL236W/322-327 YNL154C/26-31 YNL091W/805-810
KxFxS	9.65E-18	5	11	YGL150C/1279-1284 YJL092W/762-767 YLR305C/370-375 YNL154C/536-541 YNL091W/201-206
**II. *D.melanogaster* single proteins set**				
>CG5650 17933740 PP2Ac*				
KxVxF	2.3E-19	8	14	CG8316/289-294 CG9933/174-179 CG15415/742-747 CG1553/734-739 CG17360/112-117 CG15705/61-66 CG9238/103-108 CG2930/125-130
KKxVxxxD	4.1E-19	4	14	CG1553/733-741 CG32685/1829-1837 CG17360/438-446 CG9238/102-110
PxKxVxF	5.0E-17	4	14	CG8316/287-294 CG17360/110-117 CG15705/59-66 CG9238/101-108
>CG1839 18859629 Pfam:MSSP*				
HHxxxxHH	3.8E-19	6	10	CG13363/537-545 CG1981/1712-1720 CG15489/100-108 CG12212/76-84 CG15781/96-104 CG15032/247-255
HxxxHxxH	1.7E-18	6	10	CG13363/88-96 CG1981/1379-1387 CG15489/101-109 CG12212/327-335 CG15781/139-147 CG15032/247-255
HxHxxxH	8.1E-18	6	10	CG13363/538-545 CG1981/421-428 CG15489/101-108 CG12212/325-332 CG15781/137-144 CG15032/231-238
>CG14232 19920370 COIL				

VxQxSxxR	1.1E-18	4	4	CG4955/146-154 CG15092/175-183 CG4080/347-355 CG9356/169-177
>CG7818 19920926 Pfam:MT-A70				
TNxK	2.1E-16	5	5	CG5933/39-43 CG5410/591-595 CG8079/96-100 CG7757/412-416 CG4798/513-517
>CG17086 19921122 SET				
KxxxHR	2.1E-16	4	5	CG9638/396-402 CG32486/208-214 CG15288/2206-2212 CG5651/88-94
>CG8942 19921312 SIGNAL Pfam:EMI EGF EGF_like trans-membrane_domain*				
HxHxxxQ	6.7E-18	5	11	CG3056/319-326 CG1391/95-102 CG6619/532-539 CG11711/493-500 CG31122/455-462
HxxxxHHH	9.5E-18	4	11	CG3056/295-303 CG6619/525-533 CG11711/476-484 CG31122/609-617
HxHHxxxQ	2.4E-17	4	11	CG1391/95-103 CG6619/531-539 CG11711/491-499 CG31122/618-626
>CG11761 20129859 Pfam:Translin*				
VxxxRxYS	1.4E-20	4	10	CG1435/72-80 CG5012/14-22 CG8965/342-350 CG5063/30-38
>CG5808 21355677 Pfam:Pro_isomerase RRM*				
RHxxxxKK	4.5E-18	4	6	CG3918/150-158 CG1622/336-344 CG10324/515-523 CG6843/261-269
RxRSxSxS	8.3E-18	4	6	CG3918/199-207 CG1622/352-360 CG10324/421-429 CG6843/343-351
RxRSxxxS	8.6E-15	4	6	CG3918/89-97 CG1622/352-360 CG10324/421-429 CG6843/339-347
>CG1259 21355869 SIGNAL Pfam:Chitin_bind_4*				
HxxxHxHQ	2.9E-15	5	11	CG3056/309-317 CG15370/62-70 CG12726/112-120 CG11061/556-564 CG7605/85-93
>CG5053 21356743 RA COIL				
VxxVAxxV	5.5E-16	4	12	CG1099/124-132 CG32677/900-908 CG15803/157-165 CG18375/669-677
NxxxAxxA	7.0E-16	6	12	CG1099/655-663 CG17002/8-16 CG5675/238-246 CG15803/126-134 CG5859/182-190 CG18375/366-374
QRxxxQ	1.5E-15	6	12	CG11326/174-180 CG32677/785-791 CG5675/594-600 CG5859/769-775 CG7417/228-234 CG18375/238-244
>CG5450 24580844 Pfam:Dynein_light*				
IQTE	1.4E-13	3	12	CG11125/158-162 CG32130/139-143 CG33213/106-110
>CG2843 24581205 COIL*				
RxRxxSRS	9.9E-19	4	6	CG3542/607-615 CG6340/136-144 CG4877/151-159 CG6843/341-349
RxRxxxRS	2.7E-16	4	6	CG3542/607-615 CG6340/136-144 CG4877/151-159 CG6843/339-347
RSxxRxxS	1.3E-15	4	6	CG3542/630-638 CG6340/134-142 CG4877/147-155 CG6843/339-347
>CG11266 24582423 RRM*				
RSRxR	8.6E-24	6	9	CG4602/285-290 CG2843/352-357 CG6340/134-139 CG8273/361-366 CG10324/531-536 CG6843/339-344
SRxRS	6.4E-23	6	9	CG4602/286-291 CG2843/353-358 CG6340/173-178 CG8273/234-239 CG10324/532-537 CG6843/342-347
SRxRSR	3.0E-22	4	9	CG4602/286-292 CG8273/360-366 CG10324/532-538 CG6843/342-348
>CG6866 24584110 DSRM				
AxxxNSG	4.1E-15	4	6	CG4116/83-90 CG14446/603-610 CG8273/681-688 CG4288/490-497
>CG12620 24584776 Pfam:IPP-2*				
PGK	2.3E-15	6	8	CG5650/21-24 CG12396/658-661 CG1553/233-236 CG32685/195-198 CG31291/636-639 CG6477/299-302
KKxVxxxD	5.1E-15	3	8	CG1553/733-741 CG32685/1829-1837 CG9238/102-110
>CG2258 24640501 SH3*				
PPxVP	7.1E-15	4	4	CG31738/184-189 CG2225/769-774 CG6422/177-182 CG15218/343-348
>CG18259 24643110 RRM				
HxHxHxxS	4.1E-17	4	11	CG14625/650-658 CG4276/245-253 CG11905/101-109 CG33144/549-557
>CG7583 24646452 Pfam:2-Hacid_dh Pfam:2-Hacid_dh_C*				
PxDLS	8.0E-24	9	18	CG3758/258-263 CG3956/40-45 CG3340/413-418 CG4717/330-335 CG13651/388-393 CG9653/174-179 CG10318/100-105 CG7383/313-318 CG4761/498-503
PxDxS	6.0E-23	12	18	CG3758/258-263 CG3956/40-45 CG3340/413-418 CG4717/330-335 CG3850/334-339 CG13651/388-393 CG9653/174-179 CG8524/334-339 CG5460/804-809 CG10318/100-105 CG7383/313-318 CG4761/498-503

PxDLSxxS	7.2E-20	5	18	CG3340/463-471 CG13651/388-396 CG9653/174-182 CG10318/100-108 CG7383/313-321
>CG10324 24647508 G_patch COIL*				
RxRxxSRS	2.8E-23	5	9	CG4602/283-291 CG3019/896-904 CG6340/136-144 CG11274/136-144 CG6843/341-349
SxxRSRS	7.1E-23	5	9	CG4602/286-293 CG3019/899-906 CG6340/171-178 CG11274/141-148 CG6843/342-349
RSRxR	4.7E-22	5	9	CG4602/285-290 CG5808/452-457 CG3019/839-844 CG6340/134-139 CG11274/140-145 CG6843/339-344
>CG9291 24655726 Skp1*				
LxxLCxR	2.1E-18	4	6	CG2160/301-308 CG2944/240-247 CG1900/196-203 CG18171/63-70
LxDxCxR	2.2E-16	3	6	CG2160/301-308 CG2944/240-247 CG18171/63-70
DLCxR	6.6E-16	3	6	CG2160/303-308 CG2944/242-247 CG18171/65-70
>CG17666 24663466 *				
HxxxHxHH	5.2E-23	7	16	CG3851/149-157 CG15781/139-147 CG9653/271-279 CG15370/176-184 CG15032/247-255 CG1133/589-597 CG31122/608-616
HxHxxHH	8.0E-23	7	16	CG3851/149-156 CG15781/143-150 CG9653/271-278 CG15370/204-211 CG15032/231-238 CG1133/133-140 CG31122/606-613
HxxHxHxH	1.8E-22	7	16	CG3851/149-157 CG15781/136-144 CG9653/271-279 CG15370/59-67 CG15032/257-265 CG1133/130-138 CG31122/604-612
>CG11274 24663664 PWI				
DxxRSxS	2.1E-16	4	4	CG3918/127-134 CG10324/434-441 CG6843/344-351 CG31211/384-391
>CG1303 24665583 RING Pfam:Sina				
NxxxxQQ	1.6E-17	8	20	CG3736/28-35 CG2147/10-17 CG8301/432-439 CG3265/209-216 CG1965/325-332 CG31160/400-407 CG1856/337-344 CG11799/604-611 CG9715/146-153
>CG6843 24666524 COIL*				
SxKSKxxK	1.0E-29	5	11	CG5808/629-637 CG2843/205-213 CG6066/229-237 CG11274/871-879 CG8833/918-926
SKKSK	1.0E-29	5	11	CG2843/205-210 CG10324/487-492 CG6066/229-234 CG11274/871-876 CG8833/918-923
SKxSKxK	5.0E-29	5	11	CG2843/205-212 CG10324/487-494 CG6066/229-236 CG8833/904-911 CG6843/59-66
>CG7570 24667117				
QxxxxHH	3.1E-18	7	7	CG4818/138-145 CG32139/80-87 CG9653/269-276 CG15753/255-262 CG32514/82-89 CG32043/677-684 CG33141/1321-1328
QQHxQ	7.9E-18	6	7	CG32139/130-135 CG9653/462-467 CG15753/219-224 CG32514/506-511 CG32043/55-60 CG33141/10-15
QQxxQxH	3.2E-17	6	7	CG32139/80-87 CG9653/451-458 CG15753/266-273 CG32514/81-88 CG32043/51-58 CG33141/3-10
>CG3121 28571662 *				
RxRSxSxS	2.2E-36	9	14	CG4602/283-291 CG3918/199-207 CG9346/892-900 CG3019/896-904 CG1622/352-360 CG10324/421-429 CG6066/1-9 CG11274/138-146 CG6843/343-351
RSxxRxR	1.4E-31	8	14	CG4602/285-292 CG9346/858-865 CG3019/898-905 CG1622/352-359 CG10324/531-538 CG6066/1-8 CG11274/140-147 CG6843/339-346
RSxSxS	5.0E-31	10	14	CG4602/285-291 CG3918/130-136 CG9346/894-900 CG5808/176-182 CG3019/839-845 CG1622/352-358 CG10324/400-406 CG6066/1-7 CG11274/140-146 CG6843/345-351
>CG3294 28574034 COIL Pfam:zf-CCCH RRM				
RIR	9.6E-16	5	6	CG5064/60-63 CG2843/498-501 CG17136/75-78 CG1420/238-241 CG4877/83-86 CG7757/396-399
>CG8384 24650241 Pfam:TLE_N WD40*				
WRPW	1.2E-9	3	8	CG6096/174-178 CG8365/175-179 CG5927/145-149 CG8328/169-173 CG8346/220-224
**III. C.*elegans* single proteins set**				
>C03A7.4 SIGNAL Pfam:C_tripleX Pfam:DUF1096				
QxQCxxQ	4.4E-16	5	13	C03A7.4/34-41 C17G10.5/68-75 F29C12.1/462-469 M02G9.1/1117-1124 T01D1.6/143-150
>C12D8.1 Pfam:PH Pfam:Pkinase Pfam:Pkinase_C S_TKc PH S_TK_X Pfam:TROVE TRANS Pfam:7tm_4 SIGNAL Pfam:KH_1 Pfam:KH_2 KH				
QQxxxM	5.9E-16	5	10	AH6.5/131-137 C27B7.4/662-668 D1046.1/257-263 F32A11.6/241-247 Y113G7B.23/763-769
QQxP	1.2E-15	9	10	AH6.5/31-35 C16B8.3/80-84 C27B7.4/1075-1079 D1046.1/257-261 F32A11.6/241-245 Y113G7B.23/709-713 Y63D3A.5/204-208 ZC513.6/34-38 ZK643.5/414-418
QQxxPM	4.5E-15	4	10	AH6.5/131-137 D1046.1/295-301 F32A11.6/241-247 Y113G7B.23/763-769
>C14F5.5 Pfam:SH2 Pfam:SH3_1 Pfam:SH3_2 SH2 SH3				
RQN	2.6E-15	5	8	C11E4.6/735-738 D1037.3/4-7 F54D5.15/530-533 F54D5.5/1-4 T20F5.6/164-167

>C38D4.6 Pfam:Homeobox HOX				
YxxxSxLA	9.7E-20	4	40	C16A3.8/594-602 C26B2.6/55-63 C38D4.9/25-33 F59A6.1/453-461
DFxK	1.0E-16	6	40	B0024.12/30-34 B0218.3/357-361 C16A3.8/376-380 C26B2.6/138-142 F10C5.1/591-595 F59A6.1/101-105 R12B2.4/124-128 T05G5.3/20-24 Y37A1B.13/259-263 Y51H4A.17/272-276
MxxMxD	1.3E-15	5	40	C16A3.8/874-880 C38D4.9/35-41 F59A6.1/630-636 Y110A7A.10/295-301 Y51H4A.17/638-644
>C48D5.1 Pfam:Hormone_recep Pfam:zf-C4 HOLI ZnF_C4				
VxxxDxxI	1.2E-16	5	13	C16A3.8/567-575 F29G9.5/189-197 F33H2.6/274-282 R03G5.2/189-197 T22D1.9/188-196 Y17G9B.5/244-252 Y49E10.1/105-113 ZK945.2/46-54
PVVxS	3.5E-16	4	13	C16A3.8/576-581 C23G10.8/281-286 F33H2.6/272-277 T22D1.9/973-978
>C49A1.4				
QQxxxxxQ	4.8E-27	17	34	B0513.1/22-30 C02B10.5/495-503 C02F12.8/84-92 C09G1.4/178-186 C16B8.3/95-103 C27B7.4/654-662 D1046.1/283-291 F15C11.1/410-418 F56A12.1/16-24 K01B6.1/108-116 K02E10.4/287-295 K09B11.9/238-246 M142.6/582-590 R119.4/475-483 T07D1.4/29-37 Y113G7B.23/655-663 Y63D3A.5/257-265 Y79H2A.1/218-226
QQxxxxP	2.7E-26	16	34	B0513.1/26-33 C02F12.8/83-90 C09G1.4/407-414 C16B8.3/80-87 C53C7.3/64-71 D1046.1/226-233 F15C11.1/550-557 K01B6.1/123-130 K02B9.2/533-540 M142.6/581-588 R119.4/485-492 T07D1.4/13-20 Y113G7B.23/634-641 Y119C1A.1/31-38 Y63D3A.5/260-267 Y79H2A.1/284-291
PPxxxQ	1.5E-25	16	34	B0513.1/19-25 C02B10.5/634-640 C09G1.4/79-85 C16B8.3/83-89 D1046.1/221-227 F15C11.1/545-551 F57G12.2/113-119 K02B9.2/404-410 K02E10.4/333-339 M142.6/521-527 R119.4/502-508 W01A11.4/180-186 W08E12.8/124-130 Y113G7B.23/591-597 Y63D3A.5/200-206 ZK121.2/113-119
>C52B11.2 Pfam:K_tetra BTB				
VxIxxxxT	6.6E-14	5	6	C52B11.2/222-230 F54C9.11/22-30 T04H1.2/521-529 T22D1.9/820-828 Y42H9AR.1/6-14
>F14F3.1 Pfam:Homeobox Pfam:PAX HOX PAX				
PQQ	5.5E-22	12	27	B0513.1/21-24 C02B10.5/494-497 C07G1.5/551-554 C16B8.3/101-104 C27B7.4/685-688 D1046.1/268-271 F26G5.9/535-538 R02F2.5/145-148 R119.4/483-486 T22A3.3/159-162 Y79H2A.1/319-322 ZK121.2/321-324
QxxxxMxM	6.6E-21	6	27	C02B10.5/496-504 C07G1.5/507-515 C27B7.4/662-670 D1046.1/286-294 R119.4/643-651 Y79H2A.1/189-197 ZK121.2/415-423
QQQ	8.4E-20	15	27	B0513.1/22-25 C02B10.5/495-498 C02F12.8/83-86 C07G1.5/532-535 C16B8.3/124-127 C26G2.2/491-494 C27B7.4/31-34 D1046.1/286-289 F26G5.9/397-400 K02B9.2/51-54 K09B11.9/759-762 R02F2.5/146-149 R119.4/484-487 T22A3.3/187-190 Y119C1A.1/31-34 Y79H2A.1/208-211 ZK121.2/516-519
>F38A6.1 Pfam:Fork_head FH				
PxMxQ	3.1E-16	5	8	D1046.1/222-227 F26G5.9/443-448 K08F8.2/152-157 T07D1.4/247-252 Y79H2A.1/302-307
QxxQQxP	2.9E-15	5	8	D1046.1/283-290 F26G5.9/548-555 K08F8.2/257-264 T07D1.4/13-20 Y79H2A.1/330-337
QxQxQP	9.1E-15	5	8	D1046.1/284-290 F26G5.9/579-585 K08F8.2/230-236 T07D1.4/304-310 Y79H2A.1/331-337
>F44B9.6 Pfam:zf-C2H2 ZnF_C2H2				
LLxSxxF	2.2E-14	4	19	F10G8.8/625-632 F33G12.5/57-64 R11E3.6/226-233 Y54E2A.3/71-78
>F44G3.9 Pfam:zf-C4 ZnF_C4				
GFQ	1.2E-14	4	23	H02I12.5/202-205 M04F3.1/179-182 T04H1.2/516-519 T05H4.14/72-75 Y62E10A.14/202-205
>F47G6.1 COIL Pfam:ZZ ZnF_ZZ				
QxxxxxMV	1.5E-15	4	18	F59C12.3/304-312 F59C6.5/94-102 T04H1.2/126-134 Y56A3A.13/232-240
ExLxxxR	1.9E-15	9	18	B0336.6/411-418 C43C3.1/187-194 C50E3.13/232-239 F07A5.7/46-53 F44D12.1/658-665 F45E4.4/979-986 F59C12.3/172-179 F59C6.5/2-9 W07G1.3/9-16 Y56A3A.13/204-211 Y59A8B.22/361-368
RQxxxxxM	3.0E-15	5	18	F45E4.4/1327-1335 F59C6.5/110-118 T04H1.2/569-577 Y56A3A.13/231-239 Y79H2A.1/175-183
>K01G5.4 Pfam:Ras RAN				
ISxxxxF	6.5E-14	5	11	B0019.3/228-235 B0393.1/188-195 D2005.4/433-440 F53G2.6/239-246 T24F1.2/219-226 W02G9.2/284-291
>K09B11.9 COIL Pfam:Uso1_p115_head				
PPxM	7.2E-21	11	31	C06E7.4/146-150 C09G1.4/422-426 C16B8.3/64-68 C18H9.7/573-577 D1046.1/221-225 F10E9.3/175-179 F59E12.9/1361-1365 T01B6.1/2-6 T22A3.3/209-213 Y63D3A.5/267-271 ZK512.5/1133-1137
PPPxM	5.3E-20	8	31	C06E7.4/145-150 C09G1.4/421-426 C16B8.3/63-68 D1046.1/220-225 F59E12.9/1360-1365 T22A3.3/208-213 Y63D3A.5/266-271 ZK512.5/1132-1137
QQxQ	2.0E-19	13	31	C06E7.4/267-271 C09G1.4/262-266 C27B7.4/654-658 D1046.1/283-287 F29C12.1/54-58 F35H8.5/321-325 F59E12.9/919-923 K09B11.9/737-741 T09F5.10/272-276 T23G7.1/482-486 Y38H6C.14/666-670 Y43F8C.6/8-12 Y57A10A.18/1305-1309 Y63D3A.5/257-261
>R02F2.5				
NxxxGxxP	4.3E-14	4	15	C04F12.3/395-403 C18B2.4/250-258 F35H8.5/296-304 K08D10.7/1-9 K08D10.8/1-9
>T01D1.6 SIGNAL Pfam:DUF1096				
QxQCxxQ	4.2E-19	5	17	C03A7.14/34-41 C03A7.4/34-41 C17G10.5/68-75 M02G9.1/1117-1124 R09B5.5/34-41 T01D1.6/143-150 T23F1.6/212-219
PQCxxQ	8.1E-16	4	17	C03A7.14/103-109 C03A7.4/103-109 M02G9.1/427-433 R09B5.5/103-109 T01D1.6/42-48 T23F1.6/221-227

QPxCxQ	8.1E-16	4	17	C03A7.14/102-108 C03A7.4/102-108 M02G9.1/406-412 R09B5.5/102-108 T01D1.6/359-365 T23F1.6/92-98
>T04H1.2 Pfam:GTP_EFTU Pfam:GTP_EFTU_D2				
SxxSS	1.9E-35	39	64	C03C10.4/227-232 C06A5.9/345-350 C06E7.4/216-221 C06G3.6/475-480 C13F10.7/108-113 C16B8.3/7-12 C34E11.3/903-908 C52B11.2/77-82 D1005.3/7-12 D1054.13/47-52 F08F8.10/206-211 F10E9.3/103-108 F14F9.4/174-179 F23H12.5/24-29 F26G5.9/920-925 F32D1.1/418-423 F33A8.1/696-701 F42H10.7/461-466 F44D12.1/448-453 F45E6.2/42-47 F47G6.1/429-434 F52H3.4/207-212 F53G12.4/122-127 F54C9.11/28-33 F57F5.1/105-110 K01A2.10/26-31 K08F8.2/24-29 T07E3.5/62-67 T09A5.12/72-77 T11B7.1/423-428 T16H12.4/244-249 T22A3.3/65-70 T23B12.2/22-27 W03F9.10/409-414 W04A8.6/278-283 Y39B6A.1/21-26 Y42G9A.1/3-8 Y43F11A.3/123-128 Y43H11AL.1/204-209 Y46G5A.20/38-43 ZK1098.4/4-9 ZK112.2/78-83
SSxxS	1.5E-33	38	64	C03C10.4/227-232 C06A5.9/104-109 C06E7.4/239-244 C06G3.6/475-480 C13F10.7/108-113 C16B8.3/7-12 C34E11.3/1527-1532 C39E9.11/135-140 C47E8.4/115-120 C52B11.2/77-82 D1005.3/10-15 D1054.13/167-172 F08F8.10/206-211 F10E9.3/253-258 F14F9.4/17-22 F23H12.5/27-32 F32D1.1/235-240 F33A8.1/1-6 F42H10.7/465-470 F44D12.1/580-585 F45E6.2/68-73 F47G6.1/13-18 F52H3.4/206-211 F53G12.4/125-130 F54C9.11/31-36 F57F5.1/168-173 K01A2.10/28-33 K01C8.5/320-325 K08F8.2/10-15 T07E3.5/121-126 T11B7.1/422-427 T16H12.4/243-248 T22A3.3/145-150 T23B12.2/22-27 W03F9.10/584-589 W04A8.6/657-662 Y39B6A.1/106-111 Y43F11A.3/123-128 Y43H11AL.1/207-212 Y46G5A.20/38-43 Y55F3C.6/1074-1079 ZC395.8/439-444 ZK112.2/77-82
SSxS	1.5E-33	39	64	C03C10.4/227-231 C06A5.9/345-349 C06E7.4/239-243 C06G3.6/475-479 C13F10.7/108-112 C16B8.3/7-11 C34E11.3/1283-1287 C52B11.2/77-81 D1054.13/397-401 F08F8.10/206-210 F10E9.3/253-257 F23H12.5/28-32 F26G5.9/923-927 F32D1.1/236-240 F33A8.1/2-6 F42H10.7/128-132 F44D12.1/451-455 F44G3.9/271-275 F45E6.2/44-48 F47G6.1/515-519 F52H3.4/140-144 F53G12.4/125-129 F54C9.11/32-36 K01A2.10/28-32 K01C8.5/48-52 K08F8.2/24-28 T04H1.2/368-372 T07E3.5/119-123 T09A5.12/376-380 T11B7.1/426-430 T22A3.3/153-157 T23B12.2/22-26 W03F9.10/584-588 W04A8.6/657-661 Y39B6A.1/106-110 Y42G9A.1/6-10 Y43F11A.3/123-127 Y43H11AL.1/349-353 Y46G5A.20/38-42 Y55F3C.6/369-373 ZC395.8/162-166 ZK1053.3/566-570 ZK1098.4/61-65 ZK112.2/180-184
>T05C12.6 Pfam:DEP Pfam:DIX Pfam:PDZ DAX DEP PDZ				
SxxSxxxS	5.0E-17	19	31	C06G3.6/451-459 C17E7.4/293-301 C27A2.6/185-193 C37A5.9/62-70 F08F8.10/271-279 F09E5.7/77-85 F14F9.4/18-26 F36G3.1/520-528 F37A4.9/14-22 F44B9.6/34-42 F54D5.15/29-37 H15N14.2/233-241 T09A5.2/137-145 T26C12.3/65-73 W10G6.3/5-13 Y40C5A.1/130-138 Y43H11AL.1/204-212 Y79H2A.11/88-96 ZK112.2/75-83 ZK512.5/178-186
SxxxSxxS	7.2E-16	18	31	C06A5.9/341-349 C17E7.4/293-301 C27A2.6/202-210 C37A5.9/158-166 F08F8.10/264-272 F09E5.7/91-99 F36G3.1/10-18 F37A4.9/17-25 F44B9.6/478-486 F54D5.15/268-276 T09A5.2/547-555 T26C12.3/61-69 W04A8.6/465-473 W10G6.3/5-13 Y43H11AL.1/204-212 Y54E2A.3/249-257 Y79H2A.11/88-96 ZK112.2/180-188 ZK512.5/982-990
SSxxxS	1.3E-15	19	31	C06A5.9/103-109 C06G3.6/53-59 C17E7.4/292-298 C27A2.6/9-15 C37A5.9/157-163 F08F8.10/236-242 F09E5.7/90-96 F14F9.4/2-8 F36G3.1/522-528 F37A4.9/196-202 F44B9.6/716-722 F53B3.3/403-409 F54D5.15/377-383 T26C12.3/68-74 W10G6.3/45-51 Y43H11AL.1/207-213 Y79H2A.11/90-96 ZK112.2/77-83 ZK512.5/45-51
>T05H4.14 Pfam:WD40 WD40				
FxDD	8.5E-15	6	10	C06A5.9/289-293 C25G4.4/298-302 F23B12.5/164-168 F54D10.7/108-112 T08G5.5/544-548 Y66D12A.16/43-47
>T17H7.4				
SSxxxS	7.6E-24	29	53	B0336.6/170-176 C02F12.8/275-281 C06E7.4/220-226 C17E7.4/292-298 C27A2.6/9-15 C34D10.2/428-434 C43C3.1/642-648 C52B11.2/23-29 F01G10.5/189-195 F33G12.5/112-118 F36G3.1/522-528 F39H12.1/91-97 F44D12.1/580-586 F53C11.5/17-23 F54D5.15/377-383 F56F12.1/59-65 F59C12.3/510-516 F59F5.6/708-714 K02B9.2/232-238 R11E3.6/19-25 T28C6.7/95-101 W09B7.2/105-111 W10G6.3/45-51 Y119C1A.1/53-59 Y42G9A.1/6-12 Y53H1A.1/319-325 Y71H2AM.15/540-546 Y82E9BR.13/59-65 ZK1248.10/125-131
SxxSS	4.7E-21	27	53	AH6.5/13-18 B0336.6/287-292 C02F12.8/276-281 C06E7.4/216-221 C17E7.4/293-298 C27A2.6/188-193 C33G3.6/401-406 C34D10.2/360-365 C43C3.1/396-401 C52B11.2/77-82 F29G6.2/339-344 F33G12.5/109-114 F36G3.1/23-28 F44D12.1/448-453 F53C11.5/6-11 F56F12.1/99-104 F59C12.3/511-516 F59F5.6/705-710 K02B9.2/230-235 K09B11.9/414-419 R11G11.6/89-94 T28C6.7/195-200 W10G6.3/5-10 Y119C1A.1/54-59 Y42G9A.1/3-8 Y45F10D.13/2-7 Y46G5A.31/661-666 Y53H1A.1/447-452 Y71H2AM.15/263-268 Y82E9BR.13/60-65 ZK1248.10/406-411
ExxRxxxE	8.3E-21	15	53	C06A8.5/171-179 C33G3.6/253-261 C34D10.2/565-573 C43C3.1/82-90 F01G10.5/419-427 F07A5.7/146-154 F20C5.6/90-98 F28D1.2/51-59 F33G12.5/377-385 F36G3.1/162-170 F39H12.1/324-332 F42A6.9/587-595 F44D12.1/13-21 F52B10.1/504-512 F53C11.5/335-343 F54D5.15/232-240 F59C12.3/312-320 F59F5.6/182-190 K09B11.9/70-78 K12F2.1/329-337 R11E3.6/221-229 T28C6.7/50-58 W09B7.2/146-154 W10G6.3/161-169 Y42G9A.1/161-169 Y45F10D.13/25-33 Y46G5A.31/40-48 Y53H1A.1/303-311 Y54E2A.3/185-193 Y82E9BR.13/142-150 ZK1248.10/335-343
>W02G9.2 Pfam:BTB Pfam:Kelch_1 Pfam:Kelch_2 BTB Kelch				
EYA	8.9E-15	4	32	C25D7.8/186-189 C36B1.4/20-23 CD4.6/22-25 D1054.2/21-24 F25H2.9/24-27 Y106G6H.14/208-211 Y17G7B.11/407-410 ZK945.2/24-27
ExxxxAV	1.4E-14	5	32	C02F5.9/157-164 C18H9.7/229-236 C36B1.4/20-27 C40H1.6/35-42 CD4.6/224-231 D1054.2/21-28 F25H2.9/24-31 F42H10.6/5-12 Y102E9.2/51-58 Y105C5B.13/31-38 ZK945.2/24-31
DVxxD	1.5E-14	6	32	B0218.3/338-343 C40H1.6/79-84 F23F1.5/70-75 F42G10.2/246-251 F46C5.9/42-47 R53.5/126-131 Y106G6H.14/184-189
>W04D2.1 COIL Pfam:CH Pfam:Spectrin CH EFh SPEC				
KxRxA	1.3E-15	9	27	C27B7.4/215-220 C45G3.1/455-460 F22B7.13/320-325 F32D1.1/198-203 F38A6.1/341-346 F44B9.6/598-603 W07E6.4/233-238 Y106G6D.7/628-633 ZC395.8/426-431
RxxxQxxK	2.6E-15	7	27	C27B7.4/39-47 F22B7.13/96-104 F38A6.1/56-64 F47G6.1/62-70 W07E6.4/335-343 Y106G6D.7/505-513 ZK418.4/228-236
FxExA	4.7E-15	6	27	C26B2.6/315-320 F32D1.1/557-562 F42H10.7/182-187 F47G6.1/209-214 F54D5.15/309-314 K05C4.1/50-55
>W06F12.1 Pfam:Pkinase S_TKc *				
PLxIxT	7.4E-14	5	17	C06E7.4/460-466 F42A6.9/82-88 R02D5.1/163-169 Y11D7A.12/346-352 Y15E3A.5/45-51
SxPxxxS	1.1E-11	11	17	B0336.1/28-35 C06E7.4/220-227 C25G4.4/13-20 F42A6.9/26-33 F52F12.3/6-13 R02D5.1/2-9 T09F3.1/63-70 W10C8.2/71-78 Y11D7A.12/11-18 Y15E3A.5/65-72 ZK1055.7/261-268
>W07B3.2 COIL TRANS				

SSxS	2.6E-31	45	87	B0454.1/79-83 C02F12.8/279-283 C03C10.4/227-231 C06A5.9/345-349 C16B8.3/7-11 C16C4.4/281-285 C17E4.5/56-60 C17E7.4/97-101 C26G2.2/479-483 C27A2.6/206-210 C48D5.1/24-28 C50E3.13/364-368 C52B11.2/77-81 F01G12.6/206-210 F08F8.10/206-210 F08G2.7/38-42 F11C3.3/1944-1948 F13E6.4/278-282 F33G12.5/144-148 F36G3.1/229-233 F39H12.1/43-47 F40F8.5/496-500 F44G3.9/271-275 F53B3.3/299-303 F53C11.5/255-259 F59E12.9/93-97 H02I12.1/854-858 K05B2.3/440-444 K08F8.2/24-28 K09B11.9/96-100 M01E11.2/11-15 M18.7/99-103 R04B5.4/187-191 R06C1.3/42-46 T04F8.6/98-102 T05H4.2/17-21 T06G6.3/349-353 T09A5.2/137-141 T09B9.4/23-27 T09F3.1/69-73 W04A8.6/657-661 W04A8.7/44-48 W07G4.5/259-263 W10D9.4/331-335 Y119C1A.1/57-61 Y39B6A.46/220-224 Y42G9A.1/6-10 Y42H9AR.1/2-6 Y43H11AL.1/349-353 Y46G5A.31/503-507 Y53H1A.1/465-469 ZC455.1/160-164 ZK512.5/884-888
SxSxxxxR	6.7E-25	25	87	B0454.1/265-273 C14H10.2/9-17 C16B8.3/14-22 C17E7.4/83-91 C25A1.4/18-26 C27A12.2/603-611 C27A2.6/207-215 C48D5.1/341-349 C52B11.2/77-85 F08G2.7/39-47 F11C3.3/758-766 F13E6.4/198-206 F16B12.6/73-81 F33G12.5/881-889 F42A6.9/32-40 F53B3.3/224-232 F59A2.3/8-16 F59E12.9/93-101 H02I12.1/817-825 R06C1.3/183-191 T06G6.3/140-148 T09A5.2/441-449 T09B9.4/49-57 T28A8.3/120-128 W04A8.6/658-666 W04A8.7/1425-1433 W07G4.5/310-318 Y43H11AL.1/350-358 Y53H1A.1/572-580 ZC455.1/74-82 ZK512.5/686-694
SxxSS	1.0E-24	39	87	AH6.5/13-18 C02F12.8/276-281 C03C10.4/227-232 C04C3.5/322-327 C06A5.9/345-350 C14H10.2/509-514 C16B8.3/7-12 C17E7.4/293-298 C26G2.2/530-535 C27A2.6/188-193 C48D5.1/21-26 C52B11.2/77-82 D1005.3/7-12 F08F8.10/206-211 F08G2.7/122-127 F13E6.4/343-348 F14F9.4/174-179 F29D10.5/195-200 F33G12.5/109-114 F36G3.1/23-28 F40F8.5/363-368 F53C11.5/6-11 F59E12.9/1330-1335 H02I12.1/275-280 K05B2.3/8-13 K08F8.2/24-29 K09B11.9/414-419 M01E11.2/11-16 R08E3.4/31-36 R74.3/211-216 T09A5.2/134-139 T09B9.4/41-46 T09F3.1/69-74 T10B11.3/319-324 T21B6.3/167-172 W04A8.6/278-283 W04A8.7/1404-1409 W07G4.5/262-267 Y119C1A.1/54-59 Y42G9A.1/3-8 Y42H9AR.1/246-251 Y43H11AL.1/204-209 Y46G5A.31/661-666 Y53H1A.1/447-452 ZC266.1/11-16 ZC455.1/160-165 ZK512.5/33-38
>W09H1.6 Pfam:Gal-bind_lectin GLECT				
DxxNxE	7.0E-14	8	21	C07E3.3/7-13 C34F6.9/144-150 C56C10.9/201-207 F09B9.4/38-44 F55G1.8/436-442 F59C6.5/207-213 R31.1/27-33 Y39G10AL.1/135-141
>Y79H2A.1 TRANS Pfam:DCX Pfam:Pkinase DCX S_TKc				
PxDY	7.0E-16	5	13	C49A1.4/134-138 F35H8.5/280-284 R12B2.1/26-30 Y59E9AR.5/279-283 ZK418.4/82-86
>ZK945.2 COIL Pfam:Proteasome				
SSIG	4.0E-14	4	11	T08A11.1/912-916 T28C6.7/173-177 Y42H9AR.1/465-469 ZK945.2/1-5
**IV. *H.sapiens* single proteins set**				
>HPRD_00023 BBOX Pfam:zf-B_box RING				
GSxxxGxP	1.23E-44	5	21	HPRD_00524/293-301 HPRD_00673/166-174 HPRD_00744/127-135 HPRD_02910/1205-1213 HPRD_02911/1054-1062
SxxxSxxS	3.21E-44	15	21	HPRD_00673/147-155 HPRD_00744/78-86 HPRD_01241/356-364 HPRD_01271/493-501 HPRD_01272/149-157 HPRD_01796/40-48 HPRD_02487/147-155 HPRD_02534/1679-1687 HPRD_02910/745-753 HPRD_02911/696-704 HPRD_03143/112-120 HPRD_03938/300-308 HPRD_04556/444-452 HPRD_06829/52-60 HPRD_07185/464-472 HPRD_09678/220-228
SxxSS	1.71E-43	18	21	HPRD_00524/673-678 HPRD_00673/191-196 HPRD_00744/78-83 HPRD_01241/532-537 HPRD_01271/26-31 HPRD_01272/397-402 HPRD_01796/41-46 HPRD_02487/147-152 HPRD_02534/2417-2422 HPRD_02910/1572-1577 HPRD_02911/1449-1454 HPRD_03143/405-410 HPRD_03938/287-292 HPRD_04424/494-499 HPRD_04556/367-372 HPRD_06829/52-57 HPRD_07002/100-105 HPRD_07185/468-473 HPRD_09678/928-933
>HPRD_00150 SH2 SH3				
PxxPPxP	0.00E+00	36	123	HPRD_00541/282-289 HPRD_00546/861-868 HPRD_00587/442-449 HPRD_00610/44-51 HPRD_00745/134-141 HPRD_00883/40-47 HPRD_00896/470-477 HPRD_00943/1198-1205 HPRD_01140/134-141 HPRD_01267/68-75 HPRD_01320/819-826 HPRD_01381/304-311 HPRD_01681/1147-1154 HPRD_01809/896-903 HPRD_02262/477-484 HPRD_02301/336-343 HPRD_02314/311-318 HPRD_02407/168-175 HPRD_02433/27-34 HPRD_02627/284-291 HPRD_02635/105-112 HPRD_02780/43-50 HPRD_02859/894-901 HPRD_02878/857-864 HPRD_03149/1176-1183 HPRD_03583/394-401 HPRD_03657/201-208 HPRD_03926/294-301 HPRD_03991/226-233 HPRD_04404/299-306 HPRD_04620/627-634 HPRD_04633/696-703 HPRD_05026/337-344 HPRD_05116/341-348 HPRD_05136/818-825 HPRD_05153/161-168 HPRD_05434/274-281 HPRD_05448/286-293 HPRD_05480/125-132 HPRD_05674/762-769 HPRD_05796/318-325 HPRD_05866/351-358 HPRD_06571/307-314
QxxxxPxP	0.00E+00	30	123	HPRD_00114/325-333 HPRD_00171/160-168 HPRD_00540/239-247 HPRD_00546/312-320 HPRD_00610/2-10 HPRD_00737/352-360 HPRD_00883/35-43 HPRD_00896/338-346 HPRD_00943/1199-1207 HPRD_00946/1624-1632 HPRD_01044/1250-1258 HPRD_01267/204-212 HPRD_01269/447-455 HPRD_01280/1321-1329 HPRD_01281/1027-1035 HPRD_01320/489-497 HPRD_01423/1066-1074 HPRD_02314/157-165 HPRD_02407/167-175 HPRD_02433/25-33 HPRD_02513/329-337 HPRD_02545/612-620 HPRD_02790/838-846 HPRD_02859/883-891 HPRD_02878/1121-1129 HPRD_03149/562-570 HPRD_03832/74-82 HPRD_03926/352-360 HPRD_03991/515-523 HPRD_04092/845-853 HPRD_04160/111-119 HPRD_04248/150-158 HPRD_04620/623-631 HPRD_05136/539-547 HPRD_05153/301-309 HPRD_05269/218-226 HPRD_05434/363-371 HPRD_05448/274-282 HPRD_05674/760-768 HPRD_06100/687-695 HPRD_06104/718-726 HPRD_06377/435-443 HPRD_06571/444-452 HPRD_09304/212-220
PxxxPRxP	0.00E+00	13	123	HPRD_00546/448-456 HPRD_00610/32-40 HPRD_01381/84-92 HPRD_01681/1020-1028 HPRD_02301/398-406 HPRD_02314/351-359 HPRD_02635/77-85 HPRD_03149/1172-1180 HPRD_03311/23-31 HPRD_03657/207-215 HPRD_03991/272-280 HPRD_04404/85-93 HPRD_05116/500-508 HPRD_05434/189-197 HPRD_06104/735-743 HPRD_06372/146-154
>HPRD_00215 14_3_3				
HxxxxxLL	1.03E-48	9	28	HPRD_00307/398-406 HPRD_00589/500-508 HPRD_00679/725-733 HPRD_01069/308-316 HPRD_02534/66-74 HPRD_03570/627-635 HPRD_03927/79-87 HPRD_04070/686-694 HPRD_04409/37-45 HPRD_05055/641-649 HPRD_05622/382-390 HPRD_07185/395-403 HPRD_07254/224-232 HPRD_09066/686-694
RxxSxP	1.98E-48	12	28	HPRD_00307/133-139 HPRD_00679/130-136 HPRD_01069/1133-1139 HPRD_01264/360-366 HPRD_01265/255-261 HPRD_01835/193-199 HPRD_01846/256-262 HPRD_01857/561-567 HPRD_02409/308-314 HPRD_02534/377-383 HPRD_03570/958-964 HPRD_03927/997-1003 HPRD_04409/71-77 HPRD_05055/499-505 HPRD_05556/545-551 HPRD_09066/990-996 HPRD_09436/139-145
SxxSL	3.32E-47	15	28	HPRD_00307/80-85 HPRD_00589/574-579 HPRD_00679/2-7 HPRD_01069/205-210 HPRD_01264/122-127 HPRD_01265/246-251 HPRD_01819/344-349 HPRD_01835/8-13 HPRD_02534/128-133 HPRD_03212/186-191 HPRD_03390/5-10 HPRD_03570/528-533 HPRD_03927/164-169 HPRD_04070/464-469 HPRD_05055/1250-1255 HPRD_05556/392-397 HPRD_05622/594-599 HPRD_09066/667-672 HPRD_09436/176-181
>HPRD_00218 BRCT RING				

LxxAxxxQ	8.32E-55	11	59	HPRD_01238/775-783 HPRD_01793/197-205 HPRD_01796/231-239 HPRD_01938/1409-1417 HPRD_02077/928-936 HPRD_02437/184-192 HPRD_02534/360-368 HPRD_02554/2483-2491 HPRD_02668/326-334 HPRD_03013/11-19 HPRD_03143/372-380 HPRD_03354/693-701 HPRD_04078/344-352 HPRD_04386/351-359 HPRD_05521/373-381 HPRD_05797/56-64 HPRD_06347/1938-1946
PxEL	5.64E-54	11	59	HPRD_00024/943-947 HPRD_00026/26-30 HPRD_01241/73-77 HPRD_01304/178-182 HPRD_01346/133-137 HPRD_01909/312-316 HPRD_01938/46-50 HPRD_01968/961-965 HPRD_02534/40-44 HPRD_02554/1453-1457 HPRD_02777/689-693 HPRD_02806/348-352 HPRD_03143/324-328 HPRD_03301/101-105 HPRD_04078/43-47 HPRD_04231/361-365 HPRD_04232/362-366 HPRD_04366/628-632 HPRD_04386/631-635 HPRD_05405/80-84 HPRD_05521/325-329 HPRD_08916/27-31
PxSP	1.60E-52	17	59	HPRD_00024/153-157 HPRD_00026/724-728 HPRD_00993/520-524 HPRD_01238/904-908 HPRD_01303/256-260 HPRD_01304/256-260 HPRD_01818/59-63 HPRD_01909/444-448 HPRD_01938/378-382 HPRD_02407/321-325 HPRD_02534/2076-2080 HPRD_02554/3281-3285 HPRD_02668/219-223 HPRD_02777/724-728 HPRD_02806/241-245 HPRD_04078/2060-2064 HPRD_04366/518-522 HPRD_04386/330-334 HPRD_04459/108-112 HPRD_04990/160-164 HPRD_05405/114-118 HPRD_05913/439-443 HPRD_07202/11-15 HPRD_08916/1568-1572
>HPRD_00286 ARM				
PxxPP	2.76E-69	16	52	HPRD_00225/680-685 HPRD_00226/846-851 HPRD_00227/768-773 HPRD_00296/38-43 HPRD_00475/469-474 HPRD_00800/304-309 HPRD_01281/1206-1211 HPRD_01381/304-309 HPRD_01425/349-354 HPRD_01439/1368-1373 HPRD_01474/1447-1452 HPRD_01477/308-313 HPRD_01552/402-407 HPRD_01702/128-133 HPRD_01885/758-763 HPRD_02437/373-378 HPRD_02995/291-296 HPRD_03203/31-36 HPRD_03220/517-522 HPRD_03221/5-10 HPRD_03222/655-660 HPRD_03751/189-194 HPRD_03908/287-292 HPRD_03968/293-298 HPRD_04306/145-150 HPRD_04380/5-10 HPRD_04402/744-749 HPRD_04459/217-222 HPRD_04819/692-697 HPRD_04935/682-687 HPRD_04972/20-25 HPRD_06064/46-51 HPRD_06874/203-208 HPRD_08960/137-142 HPRD_09802/1219-1224
PPxxP	1.13E-68	18	52	HPRD_00296/21-26 HPRD_00475/469-474 HPRD_00579/384-389 HPRD_00800/288-293 HPRD_00918/176-181 HPRD_01381/307-312 HPRD_01439/1438-1443 HPRD_01474/1447-1452 HPRD_01477/307-312 HPRD_01552/120-125 HPRD_01702/128-133 HPRD_02437/373-378 HPRD_02955/10-15 HPRD_02995/291-296 HPRD_03007/169-174 HPRD_03203/1-6 HPRD_03220/520-525 HPRD_03222/541-546 HPRD_03751/193-198 HPRD_03780/176-181 HPRD_03908/693-698 HPRD_03968/68-73 HPRD_04402/566-571 HPRD_04459/107-112 HPRD_04596/591-596 HPRD_04935/33-38 HPRD_04972/900-905 HPRD_06064/56-61 HPRD_06874/459-464 HPRD_08960/264-269 HPRD_09802/1213-1218
PxxPxP	1.04E-64	17	52	HPRD_00225/680-686 HPRD_00226/312-318 HPRD_00296/45-51 HPRD_00475/469-475 HPRD_00579/1113-1119 HPRD_00800/289-295 HPRD_01075/247-253 HPRD_01281/1115-1121 HPRD_01381/83-89 HPRD_01439/1774-1780 HPRD_01474/1447-1453 HPRD_01477/306-312 HPRD_01481/2187-2193 HPRD_01552/1090-1096 HPRD_01702/128-134 HPRD_02437/373-379 HPRD_02995/292-298 HPRD_03007/176-182 HPRD_03203/251-257 HPRD_03220/502-508 HPRD_03221/217-223 HPRD_03222/540-546 HPRD_03751/189-195 HPRD_04380/176-182 HPRD_04402/565-571 HPRD_04417/2-8 HPRD_04459/217-223 HPRD_04819/20-26 HPRD_04935/21-27 HPRD_06874/221-227 HPRD_09802/1214-1220
>HPRD_00309 RHO				
PxxxxPP	2.11E-73	20	39	HPRD_01284/656-663 HPRD_01381/123-130 HPRD_01991/221-228 HPRD_02130/734-741 HPRD_02314/311-318 HPRD_02373/21-28 HPRD_02502/684-691 HPRD_02533/653-660 HPRD_03105/416-423 HPRD_03886/24-31 HPRD_03898/150-157 HPRD_04108/229-236 HPRD_05142/435-442 HPRD_05434/274-281 HPRD_05448/281-288 HPRD_05675/280-287 HPRD_05686/320-327 HPRD_05796/303-310 HPRD_05994/405-412 HPRD_06104/673-680 HPRD_06316/123-130 HPRD_06423/641-648 HPRD_06426/202-209 HPRD_09363/121-128 HPRD_09364/27-34 HPRD_09406/322-329
PPxP	2.33E-71	21	39	HPRD_01284/606-610 HPRD_01381/83-87 HPRD_01991/227-231 HPRD_02130/573-577 HPRD_02314/159-163 HPRD_02373/161-165 HPRD_02502/612-616 HPRD_02533/625-629 HPRD_03105/6-10 HPRD_03886/24-28 HPRD_03995/11-15 HPRD_04108/231-235 HPRD_05142/432-436 HPRD_05428/10-14 HPRD_05434/277-281 HPRD_05448/7-11 HPRD_05675/104-108 HPRD_05796/246-250 HPRD_05994/695-699 HPRD_06104/629-633 HPRD_06423/104-108 HPRD_09363/126-130 HPRD_09406/483-487 HPRD_09667/389-393 HPRD_10138/278-282
PxxxxPxP	8.06E-67	23	39	HPRD_01381/83-91 HPRD_01991/221-229 HPRD_02060/1050-1058 HPRD_02130/739-747 HPRD_02314/311-319 HPRD_02373/149-157 HPRD_02502/677-685 HPRD_02533/638-646 HPRD_02661/146-154 HPRD_03105/406-414 HPRD_03886/24-32 HPRD_03898/162-170 HPRD_03995/186-194 HPRD_04108/227-235 HPRD_05428/179-187 HPRD_05434/274-282 HPRD_05448/281-289 HPRD_05675/182-190 HPRD_05796/296-304 HPRD_05994/1132-1140 HPRD_06104/607-615 HPRD_06316/265-273 HPRD_06426/273-281 HPRD_09406/479-487 HPRD_09667/30-38
>HPRD_00350 CLH Pfam:Clathrin_propel				
LxDL	8.68E-42	9	15	HPRD_00146/334-338 HPRD_00350/283-287 HPRD_01287/765-769 HPRD_01900/444-448 HPRD_02358/331-335 HPRD_02533/793-797 HPRD_02541/164-168 HPRD_02687/350-354 HPRD_03461/268-272 HPRD_03631/1013-1017 HPRD_04637/356-360 HPRD_05085/91-95 HPRD_05765/211-215 HPRD_06270/231-235 HPRD_06273/325-329
>HPRD_00496 SH2 SH3 TyrKc				
PxxPP	1.65E-39	10	19	HPRD_00745/134-139 HPRD_00932/261-266 HPRD_00975/517-522 HPRD_01320/493-498 HPRD_01425/349-354 HPRD_01719/10-15 HPRD_02513/333-338 HPRD_02534/699-704 HPRD_02780/43-48 HPRD_02834/288-293 HPRD_02859/666-671 HPRD_03937/46-51 HPRD_05772/133-138
PPxP	2.65E-39	10	19	HPRD_00146/87-91 HPRD_00745/134-138 HPRD_01320/480-484 HPRD_02513/333-337 HPRD_02534/8-12 HPRD_02780/4-8 HPRD_02834/288-292 HPRD_02859/733-737 HPRD_03857/327-331 HPRD_03937/46-50 HPRD_05772/257-261
PPxPxR	1.56E-38	5	19	HPRD_01320/493-499 HPRD_02513/333-339 HPRD_02534/8-14 HPRD_02780/413-419 HPRD_02834/290-296 HPRD_02859/733-739
>HPRD_00574 EGF_CA EGF_like G2F LY NIDO SIGNAL TY				
CPxG	4.58E-47	7	15	HPRD_00362/94-98 HPRD_00383/701-705 HPRD_00574/1204-1208 HPRD_00619/63-67 HPRD_00620/105-109 HPRD_00629/318-322 HPRD_00630/99-103 HPRD_00804/506-510 HPRD_01030/691-695 HPRD_01428/712-716 HPRD_02796/560-564
>HPRD_00579 FU Pfam:Recep_L_domain SIGNAL TyrKc transmembrane_domain				
PxxxxPxP	2.68E-65	23	76	HPRD_00627/371-379 HPRD_00745/42-50 HPRD_00802/300-308 HPRD_00883/40-48 HPRD_01267/72-80 HPRD_01281/1129-1137 HPRD_01423/1089-1097 HPRD_01681/1017-1025 HPRD_01801/48-56 HPRD_01820/1080-1088 HPRD_02563/210-218 HPRD_02780/29-37 HPRD_02859/893-901 HPRD_02900/155-163 HPRD_03002/379-387 HPRD_03149/1142-1150 HPRD_03180/125-133 HPRD_03311/13-21 HPRD_03312/129-137 HPRD_03313/75-83 HPRD_03583/388-396 HPRD_04669/35-43 HPRD_05006/107-115 HPRD_05116/337-345 HPRD_05136/542-550 HPRD_05230/244-252 HPRD_05283/245-253 HPRD_05411/249-257 HPRD_05706/656-664 HPRD_06104/607-615 HPRD_08968/777-785
PxPxxP	2.33E-60	28	76	HPRD_00745/135-141 HPRD_00883/40-46 HPRD_01267/66-72 HPRD_01281/1146-1152 HPRD_01423/902-908 HPRD_01681/1022-1028 HPRD_01786/83-89 HPRD_01801/12-18 HPRD_01820/1208-1214 HPRD_01885/24-30 HPRD_02563/210-216 HPRD_02780/411-417 HPRD_02859/734-740 HPRD_02900/160-166 HPRD_03002/1098-1104 HPRD_03131/714-720 HPRD_03149/1145-1151 HPRD_03180/1192-1198 HPRD_03311/18-24 HPRD_03583/393-399 HPRD_03857/488-494 HPRD_04160/155-161 HPRD_04669/40-46 HPRD_04877/19-25 HPRD_05006/128-134 HPRD_05116/340-346 HPRD_05136/542-548 HPRD_05230/163-169 HPRD_05283/68-74 HPRD_05411/264-270 HPRD_05706/656-662 HPRD_06104/102-108 HPRD_08968/780-786 HPRD_09334/13-19

PxxPxxxP	2.88E-60	24	76	HPRD_00745/137-145 HPRD_00802/300-308 HPRD_00883/40-48 HPRD_01267/71-79 HPRD_01281/561-569 HPRD_01423/1062-1070 HPRD_01681/1017-1025 HPRD_01801/100-108 HPRD_01820/1141-1149 HPRD_02563/212-220 HPRD_02780/4-12 HPRD_02859/663-671 HPRD_02900/155-163 HPRD_03002/379-387 HPRD_03131/716-724 HPRD_03149/1085-1093 HPRD_03171/15-23 HPRD_03180/4536-4544 HPRD_03311/20-28 HPRD_03312/129-137 HPRD_03313/75-83 HPRD_03583/307-315 HPRD_03857/144-152 HPRD_04669/35-43 HPRD_05006/147-155 HPRD_05116/337-345 HPRD_05136/546-554 HPRD_05230/694-702 HPRD_05283/209-217 HPRD_05411/249-257 HPRD_06104/576-584 HPRD_08968/777-785 HPRD_09334/15-23
>HPRD_00589 HOLI Pfam:Oest_recep ZnF_C4				
SPxxxxS	0.00E+00	28	59	HPRD_00218/1496-1503 HPRD_00307/341-348 HPRD_00583/33-40 HPRD_00645/40-47 HPRD_01302/242-249 HPRD_01346/218-225 HPRD_01801/230-237 HPRD_01908/345-352 HPRD_02070/267-274 HPRD_02437/425-432 HPRD_02483/15-22 HPRD_02596/726-733 HPRD_02780/53-60 HPRD_02910/214-221 HPRD_02911/96-103 HPRD_03120/780-787 HPRD_03221/457-464 HPRD_03390/5-12 HPRD_03570/492-499 HPRD_03927/578-585 HPRD_04070/516-523 HPRD_04078/19-26 HPRD_04205/343-350 HPRD_04252/279-286 HPRD_04459/16-23 HPRD_04556/109-116 HPRD_04905/507-514 HPRD_05055/1020-1027 HPRD_05155/312-319 HPRD_05253/351-358 HPRD_05475/866-873 HPRD_06426/247-254 HPRD_09066/486-493 HPRD_09455/751-758
SPxxxS	5.05E-82	30	59	HPRD_00218/1124-1130 HPRD_00220/45-51 HPRD_00645/297-303 HPRD_01908/46-52 HPRD_02483/46-52 HPRD_02596/591-597 HPRD_02780/35-41 HPRD_02910/1017-1023 HPRD_02911/171-177 HPRD_03120/1073-1079 HPRD_03221/244-250 HPRD_03253/86-92 HPRD_03318/508-514 HPRD_03390/512-518 HPRD_03570/474-480 HPRD_03927/563-569 HPRD_04078/1094-1100 HPRD_04205/383-389 HPRD_04252/274-280 HPRD_04459/45-51 HPRD_04556/109-115 HPRD_04905/563-569 HPRD_05055/847-853 HPRD_05140/476-482 HPRD_05155/312-318 HPRD_05253/351-357 HPRD_05459/436-442 HPRD_05745/98-104 HPRD_06107/872-878 HPRD_06426/201-207 HPRD_09066/468-474 HPRD_09455/947-953 HPRD_09994/380-386 HPRD_10144/567-573
SxxxSP	1.64E-71	25	59	HPRD_00218/627-633 HPRD_00579/1148-1154 HPRD_00645/36-42 HPRD_01381/264-270 HPRD_01801/226-232 HPRD_02070/263-269 HPRD_02437/646-652 HPRD_02483/121-127 HPRD_02596/587-593 HPRD_02650/337-343 HPRD_02910/1474-1480 HPRD_02911/1258-1264 HPRD_03120/160-166 HPRD_03253/14-20 HPRD_03390/7-13 HPRD_03570/503-509 HPRD_03927/251-257 HPRD_04070/439-445 HPRD_04078/119-125 HPRD_04459/30-36 HPRD_04556/585-591 HPRD_05055/670-676 HPRD_05155/440-446 HPRD_05219/157-163 HPRD_05253/785-791 HPRD_06107/958-964 HPRD_06426/323-329 HPRD_09066/549-555 HPRD_09455/130-136 HPRD_10144/476-482
>HPRD_00628 EGF_like INB PSI SIGNAL VWA transmembrane_domain				
GxSC	1.83E-38	6	34	HPRD_00636/869-873 HPRD_00804/740-744 HPRD_01033/297-301 HPRD_01726/2192-2196 HPRD_01765/270-274 HPRD_02271/38-42 HPRD_02796/492-496 HPRD_04060/32-36 HPRD_04910/108-112 HPRD_05235/960-964
>HPRD_00655 SH2 SH3 TyrKc				
PxxPxxxP	0.00E+00	27	76	HPRD_00037/11-19 HPRD_00179/100-108 HPRD_00204/12-20 HPRD_00500/5-13 HPRD_00610/44-52 HPRD_01320/543-551 HPRD_01381/83-91 HPRD_01423/1062-1070 HPRD_01472/56-64 HPRD_01495/301-309 HPRD_01681/1017-1025 HPRD_01719/129-137 HPRD_02314/311-319 HPRD_02514/273-281 HPRD_02641/12-20 HPRD_02859/663-671 HPRD_03131/716-724 HPRD_03312/129-137 HPRD_03502/5-13 HPRD_03857/144-152 HPRD_03926/355-363 HPRD_03937/316-324 HPRD_04094/1049-1057 HPRD_04107/47-55 HPRD_04248/70-78 HPRD_04404/89-97 HPRD_04669/35-43 HPRD_05026/340-348 HPRD_05434/186-194 HPRD_05667/134-142 HPRD_05674/798-806 HPRD_05796/296-304 HPRD_06104/576-584 HPRD_07189/136-144 HPRD_09181/217-225
PxxxxxPP	0.00E+00	25	76	HPRD_00037/6-14 HPRD_00179/403-411 HPRD_00610/39-47 HPRD_00698/1125-1133 HPRD_01160/63-71 HPRD_01320/536-544 HPRD_01381/84-92 HPRD_01470/560-568 HPRD_01472/57-65 HPRD_01490/304-312 HPRD_01493/2-10 HPRD_01681/1082-1090 HPRD_01719/129-137 HPRD_01750/275-283 HPRD_02314/311-319 HPRD_02570/877-885 HPRD_02641/12-20 HPRD_02859/663-671 HPRD_03131/644-652 HPRD_03502/5-13 HPRD_03857/882-890 HPRD_03937/45-53 HPRD_04107/73-81 HPRD_04199/17-25 HPRD_04248/344-352 HPRD_04404/251-259 HPRD_05026/335-343 HPRD_05434/274-282 HPRD_05674/156-164 HPRD_05772/251-259 HPRD_05796/311-319 HPRD_06104/579-587 HPRD_06571/429-437 HPRD_09181/215-223
PxPP	0.00E+00	26	76	HPRD_00037/11-15 HPRD_00113/98-102 HPRD_00179/61-65 HPRD_00204/279-283 HPRD_00500/225-229 HPRD_00609/61-65 HPRD_00610/44-48 HPRD_01269/199-203 HPRD_01284/606-610 HPRD_01320/44-48 HPRD_01381/88-92 HPRD_01470/558-562 HPRD_01472/56-60 HPRD_01681/1147-1151 HPRD_01719/171-175 HPRD_01746/155-159 HPRD_01750/281-285 HPRD_02314/159-163 HPRD_02570/1049-1053 HPRD_02641/15-19 HPRD_02859/663-667 HPRD_03502/5-9 HPRD_03926/294-298 HPRD_03937/47-51 HPRD_04094/239-243 HPRD_04107/59-63 HPRD_04248/91-95 HPRD_04404/257-261 HPRD_04927/28-32 HPRD_05026/340-344 HPRD_05434/277-281 HPRD_05667/139-143 HPRD_05674/745-749 HPRD_05796/246-250 HPRD_06104/583-587 HPRD_06571/308-312 HPRD_07189/136-140 HPRD_09181/217-221
>HPRD_00679 HOLI Pfam:GCR ZnF_C4				
SPxxxxS	0.00E+00	23	52	HPRD_00026/726-733 HPRD_00296/20-27 HPRD_00583/33-40 HPRD_00679/403-410 HPRD_00855/89-96 HPRD_01238/892-899 HPRD_01250/363-370 HPRD_01251/262-269 HPRD_01265/243-250 HPRD_01302/242-249 HPRD_01801/230-237 HPRD_02172/45-52 HPRD_02437/425-432 HPRD_02483/15-22 HPRD_02534/1916-1923 HPRD_02910/214-221 HPRD_02911/96-103 HPRD_02991/195-202 HPRD_03185/185-192 HPRD_03301/192-199 HPRD_03445/593-600 HPRD_03927/578-585 HPRD_04070/516-523 HPRD_04380/415-422 HPRD_04556/109-116 HPRD_05002/30-37 HPRD_05037/192-199 HPRD_05155/312-319
PxxxLxxQ	2.91E-59	15	52	HPRD_00583/164-172 HPRD_00679/675-683 HPRD_01250/240-248 HPRD_01251/73-81 HPRD_01302/206-214 HPRD_02172/581-589 HPRD_02437/485-493 HPRD_02483/310-318 HPRD_02534/495-503 HPRD_02910/2501-2509 HPRD_02911/2423-2431 HPRD_02991/881-889 HPRD_03229/319-327 HPRD_03292/202-210 HPRD_03301/337-345 HPRD_03364/115-123 HPRD_03445/817-825 HPRD_04070/1099-1107 HPRD_05002/251-259 HPRD_05037/187-195 HPRD_05155/552-560
PxxPxP	4.43E-59	20	52	HPRD_00296/45-51 HPRD_00894/983-989 HPRD_01241/68-74 HPRD_01251/73-79 HPRD_01265/307-313 HPRD_01801/12-18 HPRD_01859/76-82 HPRD_02172/24-30 HPRD_02437/373-379 HPRD_02483/257-263 HPRD_02511/136-142 HPRD_02534/682-688 HPRD_02910/212-218 HPRD_02911/90-96 HPRD_02991/693-699 HPRD_03229/185-191 HPRD_03445/425-431 HPRD_03831/13-19 HPRD_03927/690-696 HPRD_04185/463-469 HPRD_04380/176-182 HPRD_04382/9-15 HPRD_04556/102-108 HPRD_05002/107-113 HPRD_10144/591-597
>HPRD_00745 C2 PH RasGAP SH2 SH3				
PxxxPxP	4.85E-45	18	36	HPRD_00104/229-236 HPRD_00796/48-55 HPRD_00883/40-47 HPRD_00932/322-329 HPRD_00975/329-336 HPRD_01050/934-941 HPRD_01078/140-147 HPRD_01285/17-24 HPRD_01381/84-91 HPRD_01423/720-727 HPRD_02433/27-34 HPRD_02756/743-750 HPRD_03131/719-726 HPRD_03502/5-12 HPRD_03926/58-65 HPRD_03937/45-52 HPRD_04060/1037-1044 HPRD_04108/223-230 HPRD_04228/418-425 HPRD_04352/31-38 HPRD_05006/150-157 HPRD_05411/260-267 HPRD_05772/378-385 HPRD_06569/189-196
PPxxP	1.25E-40	14	36	HPRD_00104/35-40 HPRD_00579/384-389 HPRD_00883/40-45 HPRD_00932/632-637 HPRD_00975/643-648 HPRD_01050/934-939 HPRD_01381/307-312 HPRD_01495/560-565 HPRD_01819/486-491 HPRD_02433/29-34 HPRD_02756/785-790 HPRD_03131/712-717 HPRD_03502/5-10 HPRD_03926/63-68 HPRD_03937/45-50 HPRD_04060/988-993 HPRD_04108/231-236 HPRD_05006/129-134 HPRD_05235/696-701 HPRD_05411/278-283 HPRD_05772/256-261 HPRD_06569/292-297
PxxxxPP	4.02E-38	10	36	HPRD_00104/1291-1298 HPRD_00883/40-47 HPRD_01078/134-141 HPRD_01381/123-130 HPRD_01814/436-443 HPRD_02433/27-34 HPRD_03131/719-726 HPRD_03502/6-13 HPRD_03926/58-65 HPRD_03937/45-52 HPRD_04060/1474-1481 HPRD_04108/229-236 HPRD_05006/133-140 HPRD_05411/273-280 HPRD_05772/251-258 HPRD_06569/265-272

>HPRD_00804 EGF_CA EGF_Lam IG IGc2 IGv LDLa LamB LamG SEA SIGNAL ZnF_NFX				
CPxG	3.45E-38	4	16	HPRD_00383/701-705 HPRD_00574/1204-1208 HPRD_00628/722-726 HPRD_00629/318-322 HPRD_00630/99-103 HPRD_01033/710-714 HPRD_05654/1370-1374
>HPRD_00886 14_3_3				
PxxxRxxS	3.23E-38	9	14	HPRD_01264/420-428 HPRD_01288/37-45 HPRD_01320/545-553 HPRD_01383/458-466 HPRD_02730/247-255 HPRD_03410/274-282 HPRD_03623/277-285 HPRD_04409/89-97 HPRD_06060/2283-2291 HPRD_09096/256-264 HPRD_09424/119-127
>HPRD_00918 COIL HLH				
LxxxEEE	1.02E-42	4	22	HPRD_00918/617-624 HPRD_01753/242-249 HPRD_02534/1555-1562 HPRD_02725/645-652 HPRD_03374/16-23 HPRD_03780/632-639 HPRD_04078/1519-1526 HPRD_06780/349-356
KRR	1.38E-41	6	22	HPRD_00918/618-621 HPRD_01046/129-132 HPRD_01167/90-93 HPRD_01753/167-170 HPRD_02534/667-670 HPRD_02725/646-649 HPRD_03374/37-40 HPRD_03678/254-257 HPRD_03780/633-636 HPRD_03872/98-101 HPRD_04078/646-649 HPRD_10266/190-193
>HPRD_00920 Pfam:TIG				
PVxxLxxP	1.33E-42	4	8	HPRD_01827/2199-2207 HPRD_02606/2149-2157 HPRD_02607/2100-2108 HPRD_02910/2171-2179 HPRD_10230/2663-2671
>HPRD_00975 FN3 FU Pfam:Recep_L_domain SIGNAL TyrKc ZnF_NFX transmembrane_domain				
PxxPP	1.37E-38	13	28	HPRD_00727/274-279 HPRD_00745/134-139 HPRD_00943/555-560 HPRD_01477/308-313 HPRD_01552/402-407 HPRD_02780/43-48 HPRD_02878/857-862 HPRD_03221/5-10 HPRD_03311/20-25 HPRD_05006/156-161 HPRD_05587/213-218 HPRD_05831/34-39 HPRD_06372/352-357 HPRD_06734/436-441
>HPRD_00992 B41 COIL SH2 STYKc TyrKc				
WSxWS	5.41E-48	5	32	HPRD_00737/317-322 HPRD_00896/219-224 HPRD_00990/211-216 HPRD_00998/321-326 HPRD_02052/244-249 HPRD_02824/309-314 HPRD_03446/128-133
WxxWS	4.13E-44	5	32	HPRD_00737/317-322 HPRD_00896/219-224 HPRD_00990/211-216 HPRD_00998/321-326 HPRD_02052/244-249 HPRD_02824/309-314 HPRD_03446/128-133
PxxPxP	1.61E-43	13	32	HPRD_00737/450-456 HPRD_00990/641-647 HPRD_00998/372-378 HPRD_01265/307-313 HPRD_01381/83-89 HPRD_01470/561-567 HPRD_01490/299-305 HPRD_01809/896-902 HPRD_01861/265-271 HPRD_02248/186-192 HPRD_02635/48-54 HPRD_02786/167-173 HPRD_02824/652-658 HPRD_02878/388-394 HPRD_02971/298-304 HPRD_03446/771-777 HPRD_04669/35-41
>HPRD_00993 B41 SH2 STYKc TyrKc				
PxxPxP	1.47E-47	15	49	HPRD_00587/27-33 HPRD_00737/450-456 HPRD_00998/372-378 HPRD_01265/307-313 HPRD_01281/1115-1121 HPRD_01287/30-36 HPRD_01381/83-89 HPRD_01457/268-274 HPRD_01470/561-567 HPRD_01861/265-271 HPRD_02513/355-361 HPRD_02780/411-417 HPRD_02786/167-173 HPRD_02859/739-745 HPRD_02971/298-304 HPRD_03001/260-266 HPRD_03312/129-135 HPRD_03383/698-704 HPRD_03446/771-777 HPRD_03912/2-8 HPRD_04669/35-41 HPRD_05006/133-139
PxPK	1.47E-38	8	49	HPRD_00587/30-34 HPRD_00737/808-812 HPRD_00998/375-379 HPRD_01287/154-158 HPRD_01381/84-88 HPRD_01457/271-275 HPRD_02513/332-336 HPRD_02780/5-9 HPRD_02971/301-305 HPRD_03001/875-879 HPRD_03131/712-716 HPRD_03912/484-488
PPxP	2.08E-38	11	49	HPRD_00587/445-449 HPRD_00737/450-454 HPRD_00956/22-26 HPRD_00998/372-376 HPRD_01044/268-272 HPRD_01281/1148-1152 HPRD_01284/606-610 HPRD_01381/83-87 HPRD_01457/268-272 HPRD_01861/447-451 HPRD_02513/333-337 HPRD_02780/4-8 HPRD_02786/157-161 HPRD_02859/733-737 HPRD_02971/298-302 HPRD_03131/713-717 HPRD_03383/749-753 HPRD_03542/526-530 HPRD_07205/464-468
>HPRD_01080 SH2 SH3 TyrKc				
PxxxPxP	3.88E-46	20	46	HPRD_00171/415-422 HPRD_00610/3-10 HPRD_00896/470-477 HPRD_01050/934-941 HPRD_01078/140-147 HPRD_01320/559-566 HPRD_01381/84-91 HPRD_01750/292-299 HPRD_01827/2216-2223 HPRD_02547/30-37 HPRD_03007/192-199 HPRD_03121/22-29 HPRD_03131/719-726 HPRD_03926/58-65 HPRD_03937/45-52 HPRD_04927/42-49 HPRD_05411/260-267 HPRD_05480/103-110 HPRD_05667/137-144 HPRD_05674/365-372 HPRD_05772/378-385 HPRD_08923/179-186 HPRD_09181/215-222
PPxP	2.43E-45	18	46	HPRD_00171/164-168 HPRD_00610/44-48 HPRD_00896/473-477 HPRD_01320/480-484 HPRD_01381/83-87 HPRD_01382/1-5 HPRD_01750/281-285 HPRD_01827/1728-1732 HPRD_02571/638-642 HPRD_03131/713-717 HPRD_03857/327-331 HPRD_03926/37-41 HPRD_03937/46-50 HPRD_04248/167-171 HPRD_05411/279-283 HPRD_05480/125-129 HPRD_05667/497-501 HPRD_05674/765-769 HPRD_05772/257-261 HPRD_08923/180-184 HPRD_09181/219-223
PPxxP	4.88E-45	19	46	HPRD_00171/277-282 HPRD_00474/204-209 HPRD_00500/38-43 HPRD_00610/44-49 HPRD_01050/934-939 HPRD_01320/493-498 HPRD_01381/307-312 HPRD_01382/590-595 HPRD_01495/560-565 HPRD_01750/281-286 HPRD_01827/1227-1232 HPRD_02547/1062-1067 HPRD_03007/169-174 HPRD_03131/712-717 HPRD_03926/63-68 HPRD_03937/45-50 HPRD_04248/77-82 HPRD_05411/278-283 HPRD_05480/125-130 HPRD_05674/191-196 HPRD_05772/256-261 HPRD_08923/179-184 HPRD_09181/219-224
>HPRD_01166 BASIC HLH				
PxxxxPxS	1.18E-42	9	26	HPRD_00139/10-18 HPRD_00306/98-106 HPRD_00918/180-188 HPRD_01302/235-243 HPRD_01574/780-788 HPRD_02534/472-480 HPRD_02608/31-39 HPRD_02910/207-215 HPRD_03374/23-31 HPRD_03871/25-33 HPRD_04078/499-507 HPRD_04260/226-234 HPRD_05713/49-57
SPxxxS	6.68E-42	9	26	HPRD_00918/133-139 HPRD_01574/810-816 HPRD_01577/90-96 HPRD_01796/72-78 HPRD_02534/744-750 HPRD_02788/310-316 HPRD_02807/149-155 HPRD_02910/1017-1023 HPRD_03374/10-16 HPRD_03780/26-32 HPRD_04078/1094-1100 HPRD_04260/39-45
QQQQ	8.81E-40	6	26	HPRD_00139/70-74 HPRD_01302/201-205 HPRD_01796/394-398 HPRD_02534/1966-1970 HPRD_02807/419-423 HPRD_02896/198-202 HPRD_02910/493-497 HPRD_04078/2065-2069
>HPRD_01238 ANK DEATH IPT Pfam:RHD				
PDxxxxxS	1.03E-38	10	37	HPRD_00179/362-370 HPRD_00679/75-83 HPRD_00962/325-333 HPRD_01228/31-39 HPRD_01275/289-297 HPRD_01435/534-542 HPRD_01827/571-579 HPRD_02910/113-121 HPRD_03143/80-88 HPRD_04070/708-716 HPRD_04212/234-242 HPRD_05418/47-55
>HPRD_01241 IPT Pfam:RHD				
GxxxQxxP	4.38E-52	11	42	HPRD_00549/229-237 HPRD_00660/64-72 HPRD_01250/24-32 HPRD_01754/178-186 HPRD_01796/52-60 HPRD_02436/646-654 HPRD_02534/99-107 HPRD_02556/86-94 HPRD_02910/1352-1360 HPRD_03143/377-385 HPRD_04078/443-451 HPRD_05140/108-116 HPRD_05521/378-386 HPRD_07077/466-474
QxxxGxxG	1.45E-51	12	42	HPRD_00549/222-230 HPRD_00660/196-204 HPRD_00679/167-175 HPRD_01250/209-217 HPRD_01796/98-106 HPRD_01827/438-446 HPRD_02436/598-606 HPRD_02437/87-95 HPRD_02511/108-116 HPRD_02534/206-214 HPRD_02596/248-256 HPRD_02818/517-525 HPRD_02910/350-358 HPRD_03374/27-35 HPRD_04078/189-197 HPRD_05140/53-61

PSP	1.12E-50	19	42	HPRD_00023/34-37 HPRD_00024/1046-1049 HPRD_00218/1189-1192 HPRD_00277/360-363 HPRD_00297/254-257 HPRD_00549/174-177 HPRD_00564/64-67 HPRD_01239/457-460 HPRD_01250/454-457 HPRD_01286/388-391 HPRD_01796/57-60 HPRD_01801/235-238 HPRD_01827/223-226 HPRD_02437/514-517 HPRD_02511/139-142 HPRD_02534/1882-1885 HPRD_02596/725-728 HPRD_02910/119-122 HPRD_04078/831-834 HPRD_05140/475-478 HPRD_07077/18-21
>HPRD_01261 PH S_TK_X S_TKc				
RSxSxxS	5.67E-45	7	29	HPRD_00943/266-273 HPRD_01264/724-731 HPRD_01265/617-624 HPRD_01850/1334-1341 HPRD_02070/254-261 HPRD_03312/418-425 HPRD_05223/469-476 HPRD_05866/441-448
RSxxxxSL	3.75E-41	5	29	HPRD_00943/442-450 HPRD_01264/724-732 HPRD_01265/617-625 HPRD_01850/987-995 HPRD_02811/663-671 HPRD_03312/418-426
RxRSxS	9.59E-40	5	29	HPRD_00943/264-270 HPRD_01264/358-364 HPRD_01265/253-259 HPRD_01850/933-939 HPRD_02070/252-258 HPRD_05223/467-473
>HPRD_01267 SH2 SH3				
PPxxPP	2.38E-51	10	23	HPRD_01495/560-566 HPRD_01681/1149-1155 HPRD_02433/29-35 HPRD_02627/300-306 HPRD_03131/854-860 HPRD_03240/1825-1831 HPRD_03583/393-399 HPRD_03937/45-51 HPRD_04620/138-144 HPRD_05116/340-346 HPRD_05374/388-394 HPRD_08968/767-773 HPRD_10227/539-545
PxLPxK	7.79E-50	7	23	HPRD_01259/572-578 HPRD_01681/1302-1308 HPRD_02627/301-307 HPRD_03240/1670-1676 HPRD_03583/394-400 HPRD_04620/829-835 HPRD_05374/389-395 HPRD_08968/768-774
PPxxP	1.40E-47	13	23	HPRD_00579/384-389 HPRD_01259/983-988 HPRD_01495/560-565 HPRD_01555/324-329 HPRD_01681/923-928 HPRD_02433/29-34 HPRD_02627/284-289 HPRD_02635/51-56 HPRD_03100/333-338 HPRD_03131/712-717 HPRD_03240/1768-1773 HPRD_03583/310-315 HPRD_03937/45-50 HPRD_04620/138-143 HPRD_05116/340-345 HPRD_05374/388-393 HPRD_08968/767-772 HPRD_10227/506-511
>HPRD_01275 BRLZ				
SxxxSP	2.72E-52	17	29	HPRD_00179/369-375 HPRD_00443/441-447 HPRD_00529/74-80 HPRD_00738/51-57 HPRD_01043/43-49 HPRD_01138/175-181 HPRD_01238/902-908 HPRD_01304/250-256 HPRD_01518/65-71 HPRD_01574/607-613 HPRD_02407/195-201 HPRD_02566/9-15 HPRD_02668/150-156 HPRD_02730/179-185 HPRD_02910/1474-1480 HPRD_04011/454-460 HPRD_04070/439-445 HPRD_04677/342-348 HPRD_09820/10-16
PxSxxxM	4.57E-50	7	29	HPRD_00277/344-351 HPRD_01138/46-53 HPRD_01260/38-45 HPRD_01302/240-247 HPRD_01303/256-263 HPRD_01304/256-263 HPRD_01518/12-19 HPRD_03438/102-109 HPRD_04011/513-520 HPRD_04070/406-413
LSP	5.70E-49	12	29	HPRD_00443/110-113 HPRD_00800/91-94 HPRD_01043/46-49 HPRD_01138/483-486 HPRD_01238/849-852 HPRD_01302/241-244 HPRD_01304/257-260 HPRD_01574/228-231 HPRD_01908/66-69 HPRD_02407/322-325 HPRD_02730/108-111 HPRD_02910/52-55 HPRD_04070/370-373 HPRD_04266/178-181
>HPRD_01281 FU Pfam:Recep_L_domain SIGNAL TyrKc transmembrane_domain				
PPxxxPR	1.31E-40	6	30	HPRD_00946/1392-1399 HPRD_01381/83-90 HPRD_01681/1149-1156 HPRD_02859/893-900 HPRD_03131/854-861 HPRD_03995/186-193 HPRD_06090/1005-1012
>HPRD_01284 C1 CH PH RhoGEF SH2 SH3				
PPxP	2.81E-64	14	38	HPRD_00309/178-182 HPRD_00587/445-449 HPRD_00993/499-503 HPRD_01044/268-272 HPRD_01320/480-484 HPRD_01381/83-87 HPRD_01457/268-272 HPRD_02248/188-192 HPRD_02635/52-56 HPRD_02786/157-161 HPRD_02834/288-292 HPRD_03131/713-717 HPRD_03362/185-189 HPRD_03592/46-50 HPRD_03657/204-208 HPRD_03737/19-23 HPRD_03832/140-144 HPRD_04770/306-310 HPRD_05136/504-508 HPRD_05153/164-168 HPRD_05402/193-197 HPRD_05772/257-261
PxxPxP	6.66E-57	16	38	HPRD_00113/536-542 HPRD_00579/1113-1119 HPRD_00587/27-33 HPRD_00975/1125-1131 HPRD_01050/935-941 HPRD_01265/307-313 HPRD_01320/542-548 HPRD_01381/83-89 HPRD_01457/268-274 HPRD_01490/299-305 HPRD_01495/301-307 HPRD_02248/186-192 HPRD_02635/48-54 HPRD_02710/330-336 HPRD_02786/167-173 HPRD_02824/652-658 HPRD_02834/288-294 HPRD_03362/161-167 HPRD_03592/71-77 HPRD_03657/185-191 HPRD_03832/81-87 HPRD_04669/35-41 HPRD_04770/304-310 HPRD_05136/544-550 HPRD_05153/238-244
KxxPxxP	1.14E-54	11	38	HPRD_00309/132-139 HPRD_00579/188-195 HPRD_00587/280-287 HPRD_00975/309-316 HPRD_00993/496-503 HPRD_01320/530-537 HPRD_01381/80-87 HPRD_01475/154-161 HPRD_02248/185-192 HPRD_02635/103-110 HPRD_02786/154-161 HPRD_02824/218-225 HPRD_03342/348-355 HPRD_03362/347-354 HPRD_03592/34-41 HPRD_03657/254-261 HPRD_03737/16-23 HPRD_05136/541-548 HPRD_05153/41-48
>HPRD_01301 SH2 SH3 TyrKc				
PPxP	1.45E-43	16	50	HPRD_00587/445-449 HPRD_00737/450-454 HPRD_00739/132-136 HPRD_00993/499-503 HPRD_01187/10-14 HPRD_01320/480-484 HPRD_02248/188-192 HPRD_02378/595-599 HPRD_02527/81-85 HPRD_02780/4-8 HPRD_03131/713-717 HPRD_03135/309-313 HPRD_04094/238-242 HPRD_04248/167-171 HPRD_05667/497-501 HPRD_05772/257-261 HPRD_05866/351-355 HPRD_05926/35-39 HPRD_06571/307-311 HPRD_06572/517-521 HPRD_09819/508-512
PPxxP	4.23E-40	20	50	HPRD_00113/170-175 HPRD_00474/204-209 HPRD_00587/26-31 HPRD_00737/121-126 HPRD_00739/547-552 HPRD_01187/10-15 HPRD_01320/493-498 HPRD_02378/595-600 HPRD_02527/81-86 HPRD_02710/329-334 HPRD_02780/410-415 HPRD_02941/1729-1734 HPRD_03131/712-717 HPRD_03199/340-345 HPRD_04094/238-243 HPRD_04248/77-82 HPRD_05772/256-261 HPRD_05866/350-355 HPRD_05926/178-183 HPRD_06571/306-311 HPRD_06572/328-333 HPRD_09819/508-513
PPKP	1.20E-38	6	50	HPRD_00993/499-503 HPRD_01320/822-826 HPRD_02780/4-8 HPRD_03131/713-717 HPRD_05866/354-358 HPRD_05926/35-39 HPRD_06571/310-314
>HPRD_01302 BRLZ Pfam:Jun				
PxxxxPG	0.00E+00	20	65	HPRD_00179/398-405 HPRD_00180/167-174 HPRD_00443/206-213 HPRD_00643/74-81 HPRD_00673/21-28 HPRD_01043/344-351 HPRD_01166/180-187 HPRD_01271/81-88 HPRD_01518/194-201 HPRD_02436/157-164 HPRD_02437/499-506 HPRD_02534/682-689 HPRD_02995/292-299 HPRD_03221/217-224 HPRD_03343/80-87 HPRD_03438/34-41 HPRD_03927/690-697 HPRD_04023/157-164 HPRD_04070/1303-1310 HPRD_04378/652-659 HPRD_04380/176-183 HPRD_04395/20-27 HPRD_06007/92-99 HPRD_06320/86-93 HPRD_06817/161-168 HPRD_09896/183-190
LxSP	0.00E+00	21	65	HPRD_00026/665-669 HPRD_00179/414-418 HPRD_00180/327-331 HPRD_00277/291-295 HPRD_00440/119-123 HPRD_00443/87-91 HPRD_00536/1210-1214 HPRD_00549/9-13 HPRD_00643/262-266 HPRD_00673/89-93 HPRD_00679/223-227 HPRD_01166/2-6 HPRD_01223/295-299 HPRD_01271/498-502 HPRD_01275/371-375 HPRD_01302/60-64 HPRD_01574/808-812 HPRD_02511/13-17 HPRD_02534/16-20 HPRD_03343/317-321 HPRD_03479/260-264 HPRD_03927/668-672 HPRD_04070/514-518 HPRD_04266/165-169 HPRD_04378/510-514 HPRD_04517/435-439 HPRD_05349/208-212 HPRD_05668/329-333 HPRD_06007/135-139 HPRD_06817/15-19 HPRD_07455/301-305

SPxxxS	6.85E-84	26	65	HPRD_00179/373-379 HPRD_00180/360-366 HPRD_00549/11-17 HPRD_00673/191-197 HPRD_00679/403-409 HPRD_01043/47-53 HPRD_01223/378-384 HPRD_01271/500-506 HPRD_01275/20-26 HPRD_01298/80-86 HPRD_01305/118-124 HPRD_01518/76-82 HPRD_01574/810-816 HPRD_01796/72-78 HPRD_02436/544-550 HPRD_02534/744-750 HPRD_02566/13-19 HPRD_03221/244-250 HPRD_03343/229-235 HPRD_03927/563-569 HPRD_04023/156-162 HPRD_04378/248-254 HPRD_04380/207-213 HPRD_04517/499-505 HPRD_05140/476-482 HPRD_05668/331-337 HPRD_05895/88-94 HPRD_06320/780-786 HPRD_06817/17-23 HPRD_07455/33-39 HPRD_09310/254-260 HPRD_09896/136-142
>HPRD_01320 Pfam:Cbl_N Pfam:Cbl_N2 Pfam:Cbl_N3 RING UBA				
KxxxPPxP	4.62E-52	5	39	HPRD_01381/79-87 HPRD_01746/153-161 HPRD_01809/895-903 HPRD_02248/184-192 HPRD_02301/545-553 HPRD_02780/407-415 HPRD_03362/280-288 HPRD_05026/336-344
KPxxP	1.57E-46	8	39	HPRD_00796/244-249 HPRD_01080/227-232 HPRD_01301/229-234 HPRD_01381/87-92 HPRD_01746/154-159 HPRD_01809/758-763 HPRD_02248/185-190 HPRD_02301/338-343 HPRD_02900/137-142 HPRD_03131/715-720 HPRD_03362/84-89 HPRD_05026/239-244 HPRD_05587/314-319 HPRD_06100/793-798
PxPP	2.33E-46	11	39	HPRD_00113/98-102 HPRD_01269/199-203 HPRD_01284/606-610 HPRD_01288/477-481 HPRD_01381/88-92 HPRD_01746/155-159 HPRD_01809/611-615 HPRD_01819/484-488 HPRD_01866/36-40 HPRD_02248/186-190 HPRD_02301/339-343 HPRD_02780/410-414 HPRD_02900/122-126 HPRD_03362/186-190 HPRD_03596/105-109 HPRD_04133/455-459 HPRD_04404/257-261 HPRD_05026/340-344 HPRD_05587/210-214 HPRD_06100/692-696 HPRD_09411/798-802
>HPRD_01346 CYCLIN Pfam:Cyclin_C				
KRK	1.31E-43	9	27	HPRD_00024/184-187 HPRD_00218/502-505 HPRD_00298/160-163 HPRD_02436/281-284 HPRD_02437/845-848 HPRD_03247/180-183 HPRD_03570/16-19 HPRD_04070/17-20 HPRD_04078/333-336 HPRD_05258/303-306 HPRD_06780/427-430 HPRD_07185/24-27
>HPRD_01369 HOLI ZnF_C4				
SPxD	2.09E-41	10	15	HPRD_01577/259-263 HPRD_02911/96-100 HPRD_03646/70-74 HPRD_03927/806-810 HPRD_04070/21-25 HPRD_04078/1094-1098 HPRD_05055/226-230 HPRD_05155/265-269 HPRD_05253/351-355 HPRD_06107/872-876
GSxxQxxP	1.87E-39	4	15	HPRD_02911/1054-1062 HPRD_04078/2269-2277 HPRD_05055/1252-1260 HPRD_06107/940-948
LSE	1.41E-38	11	15	HPRD_00777/458-461 HPRD_01241/266-269 HPRD_02911/71-74 HPRD_03165/118-121 HPRD_03927/745-748 HPRD_04070/968-971 HPRD_04078/80-83 HPRD_05055/444-447 HPRD_05155/37-40 HPRD_05253/1649-1652 HPRD_06107/372-375 HPRD_07211/168-171
>HPRD_01381 COIL RhoGAP SH2 SH3				
PPxP	0.00E+00	26	71	HPRD_00309/178-182 HPRD_00475/469-473 HPRD_00587/445-449 HPRD_00610/44-48 HPRD_00745/134-138 HPRD_00896/473-477 HPRD_00943/154-158 HPRD_00993/499-503 HPRD_01281/1148-1152 HPRD_01284/606-610 HPRD_01320/480-484 HPRD_01382/1-5 HPRD_01423/36-40 HPRD_01820/1207-1211 HPRD_02301/339-343 HPRD_02314/159-163 HPRD_02381/356-360 HPRD_02433/29-33 HPRD_02518/183-187 HPRD_02545/4-8 HPRD_02571/638-642 HPRD_02572/72-76 HPRD_02635/52-56 HPRD_02756/48-52 HPRD_02780/4-8 HPRD_02859/733-737 HPRD_02878/8-12 HPRD_02971/298-302 HPRD_03131/713-717 HPRD_04108/231-235 HPRD_04161/268-272 HPRD_04248/167-171 HPRD_04620/627-631 HPRD_05026/340-344 HPRD_05266/421-425 HPRD_05687/488-492 HPRD_05866/351-355 HPRD_08923/180-184
PxxxPxP	8.48E-72	27	71	HPRD_00475/469-476 HPRD_00587/26-33 HPRD_00610/3-10 HPRD_00745/134-141 HPRD_00896/470-477 HPRD_00932/322-329 HPRD_00943/616-623 HPRD_01078/140-147 HPRD_01266/1069-1076 HPRD_01281/1101-1108 HPRD_01320/559-566 HPRD_01423/720-727 HPRD_01470/560-567 HPRD_01820/983-990 HPRD_02301/336-343 HPRD_02314/215-222 HPRD_02405/193-200 HPRD_02433/27-34 HPRD_02545/403-410 HPRD_02572/640-647 HPRD_02635/77-84 HPRD_02756/743-750 HPRD_02780/30-37 HPRD_02859/884-891 HPRD_02878/460-467 HPRD_03131/719-726 HPRD_04092/538-545 HPRD_04108/223-230 HPRD_04161/263-270 HPRD_04620/627-634 HPRD_05026/337-344 HPRD_05085/289-296 HPRD_05687/437-444 HPRD_05866/351-358 HPRD_08923/179-186
PxxPxP	4.26E-70	25	71	HPRD_00113/536-542 HPRD_00475/469-475 HPRD_00587/27-33 HPRD_00610/44-50 HPRD_00745/134-140 HPRD_00943/826-832 HPRD_01281/1115-1121 HPRD_01287/30-36 HPRD_01320/542-548 HPRD_01470/561-567 HPRD_01490/299-305 HPRD_01727/143-149 HPRD_01814/392-398 HPRD_01820/256-262 HPRD_02301/336-342 HPRD_02314/159-165 HPRD_02405/181-187 HPRD_02433/27-33 HPRD_02518/183-189 HPRD_02545/614-620 HPRD_02572/568-574 HPRD_02635/48-54 HPRD_02756/785-791 HPRD_02780/411-417 HPRD_02859/739-745 HPRD_02878/388-394 HPRD_02971/298-304 HPRD_04092/831-837 HPRD_04108/229-235 HPRD_04248/81-87 HPRD_04620/627-633 HPRD_05026/186-192 HPRD_05085/347-353 HPRD_05706/658-664 HPRD_05866/352-358 HPRD_08923/180-186
>HPRD_01398 C2 PH PLCXc PLCYc SH2 SH3				
PPxP	1.83E-63	20	49	HPRD_00104/1441-1445 HPRD_01281/1148-1152 HPRD_01284/606-610 HPRD_01320/480-484 HPRD_01423/36-40 HPRD_01681/1150-1154 HPRD_01746/157-161 HPRD_02314/159-163 HPRD_02545/4-8 HPRD_02635/52-56 HPRD_02678/380-384 HPRD_02859/733-737 HPRD_02878/8-12 HPRD_03149/754-758 HPRD_03260/142-146 HPRD_03362/185-189 HPRD_03657/204-208 HPRD_03832/140-144 HPRD_03857/327-331 HPRD_03926/37-41 HPRD_03972/574-578 HPRD_03995/11-15 HPRD_04320/636-640 HPRD_04860/354-358 HPRD_05116/309-313 HPRD_05668/8-12 HPRD_06391/368-372 HPRD_06577/473-477
PxPxxP	5.00E-60	22	49	HPRD_00104/1026-1032 HPRD_01266/950-956 HPRD_01281/1146-1152 HPRD_01287/868-874 HPRD_01301/228-234 HPRD_01320/531-537 HPRD_01423/902-908 HPRD_01492/703-709 HPRD_01681/1022-1028 HPRD_01727/141-147 HPRD_01746/155-161 HPRD_02314/159-165 HPRD_02545/108-114 HPRD_02635/106-112 HPRD_02678/381-387 HPRD_02859/734-740 HPRD_02878/459-465 HPRD_03121/289-295 HPRD_03149/1145-1151 HPRD_03260/140-146 HPRD_03362/348-354 HPRD_03657/204-210 HPRD_03832/79-85 HPRD_03857/488-494 HPRD_03926/62-68 HPRD_03972/573-579 HPRD_03995/39-45 HPRD_04320/637-643 HPRD_04860/324-330 HPRD_05116/340-346 HPRD_05668/9-15 HPRD_05706/656-662 HPRD_06391/366-372 HPRD_06577/476-482
PxPxxxP	2.03E-58	21	49	HPRD_00579/1116-1123 HPRD_00655/33-40 HPRD_00796/476-483 HPRD_01266/950-957 HPRD_01281/1149-1156 HPRD_01320/544-551 HPRD_01423/1023-1030 HPRD_01429/1020-1027 HPRD_01681/1147-1154 HPRD_01819/484-491 HPRD_02314/215-222 HPRD_02545/108-115 HPRD_02570/633-640 HPRD_02678/381-388 HPRD_02859/663-670 HPRD_02878/391-398 HPRD_03149/1180-1187 HPRD_03260/477-484 HPRD_03362/186-193 HPRD_03657/205-212 HPRD_03832/58-65 HPRD_03926/62-69 HPRD_03972/538-545 HPRD_03995/185-192 HPRD_04320/472-479 HPRD_04860/367-374 HPRD_05668/34-41 HPRD_06391/516-523 HPRD_06577/474-481 HPRD_09304/217-224
>HPRD_01452 PROF				
PxPxPP	4.75E-40	6	8	HPRD_01164/1348-1354 HPRD_01381/86-92 HPRD_03415/171-177 HPRD_03852/782-788 HPRD_04893/186-192 HPRD_05434/277-283 HPRD_05448/276-282
>HPRD_01470 PTPc SH2				
WSxWS	4.85E-77	4	53	HPRD_00587/232-237 HPRD_00737/317-322 HPRD_00739/424-429 HPRD_00990/211-216 HPRD_01048/234-239 HPRD_01457/214-219 HPRD_02824/309-314 HPRD_03001/318-323
WxxWS	4.24E-63	4	53	HPRD_00587/232-237 HPRD_00737/317-322 HPRD_00739/424-429 HPRD_00990/211-216 HPRD_01048/234-239 HPRD_01457/147-152 HPRD_02824/309-314 HPRD_03001/318-323

WSxW	2.03E-61	4	53	HPRD_00587/232-236 HPRD_00737/317-321 HPRD_00739/424-428 HPRD_00990/211-215 HPRD_01048/234-238 HPRD_01457/214-218 HPRD_02824/309-313 HPRD_03001/318-322
>HPRD_01475 PTPc SH2				
PPxP	3.17E-46	19	51	HPRD_00587/445-449 HPRD_00739/132-136 HPRD_00845/115-119 HPRD_00990/691-695 HPRD_00993/499-503 HPRD_01044/268-272 HPRD_01192/13-17 HPRD_01226/1107-1111 HPRD_01284/606-610 HPRD_01295/180-184 HPRD_01320/480-484 HPRD_01381/83-87 HPRD_01861/447-451 HPRD_02971/298-302 HPRD_03026/357-361 HPRD_03131/713-717 HPRD_03362/185-189 HPRD_04228/80-84 HPRD_04620/627-631 HPRD_05153/164-168 HPRD_05772/257-261 HPRD_05866/351-355 HPRD_09405/169-173
PxxPxxL	1.08E-42	12	51	HPRD_00579/385-392 HPRD_00587/445-452 HPRD_00739/132-139 HPRD_00975/873-880 HPRD_00990/122-129 HPRD_01048/135-142 HPRD_01050/559-566 HPRD_01078/152-159 HPRD_01192/64-71 HPRD_01226/1107-1114 HPRD_01295/557-564 HPRD_01320/493-500 HPRD_01381/88-95 HPRD_01495/270-277 HPRD_01861/447-454 HPRD_02824/125-132 HPRD_02971/148-155 HPRD_03131/855-862 HPRD_04228/281-288 HPRD_04620/995-1002 HPRD_05153/164-171 HPRD_05631/7-14 HPRD_05772/257-264 HPRD_06196/298-305 HPRD_09405/196-203
PPxxxxL	1.56E-39	14	51	HPRD_00191/29-36 HPRD_00587/445-452 HPRD_00739/132-139 HPRD_00845/178-185 HPRD_00975/520-527 HPRD_01078/455-462 HPRD_01165/173-180 HPRD_01226/1107-1114 HPRD_01320/80-87 HPRD_01381/128-135 HPRD_01490/310-317 HPRD_01495/564-571 HPRD_01861/447-454 HPRD_03131/650-657 HPRD_03362/240-247 HPRD_04228/131-138 HPRD_04620/1067-1074 HPRD_05153/129-136 HPRD_05631/282-289 HPRD_05772/257-264 HPRD_05866/510-517
>HPRD_01495 SH2 TyrKc				
PPVP	1.54E-50	6	27	HPRD_01267/68-72 HPRD_01320/493-497 HPRD_01457/268-272 HPRD_01681/1150-1154 HPRD_03657/206-210 HPRD_05136/504-508 HPRD_05772/257-261 HPRD_08923/180-184
PPPxP	5.61E-48	10	27	HPRD_01320/542-547 HPRD_01681/1149-1154 HPRD_01809/898-903 HPRD_02635/51-56 HPRD_02780/410-415 HPRD_03131/712-717 HPRD_03657/205-210 HPRD_05136/553-558 HPRD_05772/256-261 HPRD_05866/350-355 HPRD_08923/179-184
PPxP	4.66E-43	13	27	HPRD_00745/134-138 HPRD_01267/68-72 HPRD_01284/606-610 HPRD_01320/480-484 HPRD_01457/268-272 HPRD_01681/1150-1154 HPRD_01809/898-902 HPRD_02635/52-56 HPRD_02780/4-8 HPRD_03131/713-717 HPRD_03657/204-208 HPRD_05136/504-508 HPRD_05772/257-261 HPRD_05866/351-355 HPRD_08923/180-184
>HPRD_01503 WD40				
EFxExE	4.52E-38	4	32	HPRD_00697/1100-1106 HPRD_01574/47-53 HPRD_01852/406-412 HPRD_03587/628-634 HPRD_03851/377-383
>HPRD_01574 CYCLIN Pfam:RB_A				
PxxPxP	1.91E-56	24	61	HPRD_00023/20-26 HPRD_00295/1101-1107 HPRD_00443/206-212 HPRD_00532/709-715 HPRD_01035/652-658 HPRD_01166/234-240 HPRD_01265/307-313 HPRD_01801/12-18 HPRD_01806/68-74 HPRD_01809/896-902 HPRD_02416/216-222 HPRD_02436/156-162 HPRD_02437/373-379 HPRD_02806/5-11 HPRD_02814/296-302 HPRD_02826/414-420 HPRD_03120/969-975 HPRD_03624/220-226 HPRD_04015/22-28 HPRD_04231/156-162 HPRD_04232/157-163 HPRD_04357/158-164 HPRD_04459/217-223 HPRD_04573/111-117 HPRD_04839/664-670 HPRD_05143/1873-1879 HPRD_05831/38-44 HPRD_10144/591-597
SxxxExxD	1.66E-45	18	61	HPRD_00023/569-577 HPRD_00295/1086-1094 HPRD_00532/191-199 HPRD_01272/165-173 HPRD_01420/340-348 HPRD_01575/158-166 HPRD_01806/402-410 HPRD_01808/304-312 HPRD_01809/1118-1126 HPRD_02416/243-251 HPRD_02436/194-202 HPRD_02806/30-38 HPRD_02814/712-720 HPRD_02826/387-395 HPRD_03120/1669-1677 HPRD_03364/93-101 HPRD_04357/464-472 HPRD_04459/1078-1086 HPRD_05143/463-471 HPRD_05529/198-206 HPRD_05831/264-272 HPRD_06007/137-145 HPRD_10144/370-378
SDS	3.67E-45	17	61	HPRD_00295/1097-1100 HPRD_00443/178-181 HPRD_00532/519-522 HPRD_01272/115-118 HPRD_01275/368-371 HPRD_01575/692-695 HPRD_01818/219-222 HPRD_02416/10-13 HPRD_02436/206-209 HPRD_02814/758-761 HPRD_02826/137-140 HPRD_03120/778-781 HPRD_03143/262-265 HPRD_03483/157-160 HPRD_03624/158-161 HPRD_04459/654-657 HPRD_04990/308-311 HPRD_05143/1373-1376 HPRD_06007/143-146 HPRD_09498/312-315 HPRD_10144/781-784
>HPRD_01577 HOLI ZnF_C4				
SxxxSP	3.51E-53	17	39	HPRD_00179/369-375 HPRD_01166/196-202 HPRD_01238/902-908 HPRD_01250/359-365 HPRD_01369/72-78 HPRD_02660/179-185 HPRD_02788/306-312 HPRD_03493/176-182 HPRD_03570/503-509 HPRD_03927/251-257 HPRD_04070/439-445 HPRD_04556/585-591 HPRD_05055/670-676 HPRD_05141/834-840 HPRD_05155/440-446 HPRD_05219/157-163 HPRD_06107/958-964 HPRD_07185/464-470 HPRD_09267/41-47 HPRD_09455/130-136
QxxSxxS	2.71E-50	16	39	HPRD_00179/296-303 HPRD_00660/22-29 HPRD_00899/197-204 HPRD_01250/81-88 HPRD_01435/270-277 HPRD_02788/289-296 HPRD_03493/12-19 HPRD_03570/1125-1132 HPRD_03584/4-11 HPRD_03927/483-490 HPRD_04070/458-465 HPRD_04346/382-389 HPRD_04556/445-452 HPRD_05055/668-675 HPRD_05140/216-223 HPRD_05141/1153-1160 HPRD_06107/70-77 HPRD_07185/431-438 HPRD_09994/13-20
SxSS	4.32E-50	21	39	HPRD_00179/299-303 HPRD_00584/408-412 HPRD_00660/53-57 HPRD_00899/152-156 HPRD_01250/357-361 HPRD_01369/76-80 HPRD_02511/155-159 HPRD_02660/190-194 HPRD_02788/252-256 HPRD_03570/459-463 HPRD_03584/7-11 HPRD_03927/138-142 HPRD_04070/99-103 HPRD_04556/112-116 HPRD_04827/71-75 HPRD_05055/286-290 HPRD_05141/97-101 HPRD_05142/295-299 HPRD_05155/123-127 HPRD_06107/73-77 HPRD_07185/468-472 HPRD_07260/311-315 HPRD_09455/298-302
>HPRD_01617 COIL RRM				
SRSxSxxR	9.46E-59	5	9	HPRD_02887/204-212 HPRD_02888/127-135 HPRD_03599/248-256 HPRD_04720/591-599 HPRD_09185/222-230
SRSxSRxR	2.40E-57	5	9	HPRD_02887/204-212 HPRD_02888/127-135 HPRD_03599/248-256 HPRD_04720/591-599 HPRD_09185/222-230
SRSxSxSR	1.97E-56	5	9	HPRD_02887/204-212 HPRD_02888/127-135 HPRD_03599/248-256 HPRD_04720/617-625 HPRD_09185/222-230
>HPRD_01796 ZnF_C2H2				
PxxxPxP	2.12E-63	21	40	HPRD_00023/4-11 HPRD_00180/218-225 HPRD_00584/161-168 HPRD_00866/142-149 HPRD_00883/40-47 HPRD_01241/67-74 HPRD_01250/521-528 HPRD_01574/19-26 HPRD_01701/89-96 HPRD_01702/124-131 HPRD_01753/96-103 HPRD_01801/8-15 HPRD_01808/372-379 HPRD_02061/127-134 HPRD_02692/312-319 HPRD_02726/128-135 HPRD_02780/30-37 HPRD_02810/247-254 HPRD_02829/47-54 HPRD_02995/291-298 HPRD_03632/165-172 HPRD_03769/204-211 HPRD_06903/9-16 HPRD_10196/75-82
PxxPP	5.99E-54	19	40	HPRD_00023/20-25 HPRD_00866/237-242 HPRD_00883/40-45 HPRD_01241/310-315 HPRD_01574/19-24 HPRD_01701/95-100 HPRD_01702/128-133 HPRD_01753/214-219 HPRD_01801/12-17 HPRD_01806/334-339 HPRD_02061/420-425 HPRD_02596/270-275 HPRD_02721/125-130 HPRD_02726/466-471 HPRD_02780/43-48 HPRD_02809/393-398 HPRD_02810/396-401 HPRD_02995/291-296 HPRD_03221/5-10 HPRD_03527/290-295 HPRD_03769/348-353 HPRD_06903/9-14 HPRD_10196/43-48

QQxxxQ	3.13E-53	10	40	HPRD_00883/17-23 HPRD_01241/461-467 HPRD_01250/88-94 HPRD_01302/212-218 HPRD_01702/106-112 HPRD_02061/826-832 HPRD_02596/621-627 HPRD_02692/206-212 HPRD_02693/407-413 HPRD_02721/296-302 HPRD_02764/404-410 HPRD_02810/181-187 HPRD_03483/403-409 HPRD_06903/119-125 HPRD_10196/44-50
>HPRD_01801 BRLZ				
PxxPxP	2.28E-56	19	35	HPRD_00296/45-51 HPRD_00297/17-23 HPRD_00443/206-212 HPRD_00549/206-212 HPRD_00579/1113-1119 HPRD_00645/119-125 HPRD_00778/360-366 HPRD_01043/239-245 HPRD_01241/68-74 HPRD_01305/4-10 HPRD_01574/19-25 HPRD_01810/463-469 HPRD_02407/171-177 HPRD_02566/248-254 HPRD_02834/288-294 HPRD_02995/292-298 HPRD_03435/328-334 HPRD_03445/425-431 HPRD_03864/295-301 HPRD_04078/819-825 HPRD_04380/176-182 HPRD_04459/217-223
PxxxPxP	6.03E-51	18	35	HPRD_00296/38-45 HPRD_00297/156-163 HPRD_00443/209-216 HPRD_00645/11-18 HPRD_00778/238-245 HPRD_01043/242-249 HPRD_01241/67-74 HPRD_01305/92-99 HPRD_01574/19-26 HPRD_01818/230-237 HPRD_02407/168-175 HPRD_02566/247-254 HPRD_02829/47-54 HPRD_02834/284-291 HPRD_02995/291-298 HPRD_03302/734-741 HPRD_03435/991-998 HPRD_03445/424-431 HPRD_03864/294-301 HPRD_04078/550-557 HPRD_04380/204-211 HPRD_04459/11-18
PPxxxP	4.87E-49	17	35	HPRD_00296/181-187 HPRD_00297/162-168 HPRD_00549/235-241 HPRD_00589/113-119 HPRD_00645/121-127 HPRD_00778/360-366 HPRD_01043/424-430 HPRD_01241/241-247 HPRD_01574/19-25 HPRD_02407/171-177 HPRD_02788/224-230 HPRD_02834/288-294 HPRD_03302/791-797 HPRD_03435/330-336 HPRD_03445/752-758 HPRD_03864/293-299 HPRD_04078/865-871 HPRD_04380/135-141 HPRD_04459/275-281 HPRD_05436/942-948
>HPRD_01809 SH2 SH3 TyrKc				
PPxP	3.11E-63	19	37	HPRD_00780/71-75 HPRD_01044/268-272 HPRD_01259/983-987 HPRD_01272/312-316 HPRD_01295/180-184 HPRD_01320/480-484 HPRD_01574/19-23 HPRD_02476/28-32 HPRD_02756/48-52 HPRD_02900/138-142 HPRD_03587/167-171 HPRD_03937/46-50 HPRD_05374/389-393 HPRD_05428/10-14 HPRD_05587/146-150 HPRD_05772/257-261 HPRD_05813/262-266 HPRD_05891/277-281 HPRD_07338/180-184 HPRD_09181/219-223 HPRD_10227/458-462
PxxxPP	2.80E-51	17	37	HPRD_00780/34-40 HPRD_01044/15-21 HPRD_01259/506-512 HPRD_01295/670-676 HPRD_01320/531-537 HPRD_01495/560-566 HPRD_01574/19-25 HPRD_01859/7-13 HPRD_01869/742-748 HPRD_02476/13-19 HPRD_02756/785-791 HPRD_02900/934-940 HPRD_02941/1153-1159 HPRD_03109/179-185 HPRD_03937/45-51 HPRD_04788/328-334 HPRD_05374/388-394 HPRD_05428/71-77 HPRD_05587/206-212 HPRD_05813/258-264 HPRD_06347/1373-1379 HPRD_07338/171-177 HPRD_09181/215-221 HPRD_10227/492-498
PPPxxxxP	3.60E-51	12	37	HPRD_00780/32-40 HPRD_01259/1041-1049 HPRD_01320/543-551 HPRD_01574/25-33 HPRD_02476/30-38 HPRD_02900/937-945 HPRD_03587/483-491 HPRD_03937/45-53 HPRD_05374/388-396 HPRD_05587/210-218 HPRD_05813/263-271 HPRD_07338/403-411 HPRD_09181/221-229 HPRD_10227/539-547
>HPRD_01813 RAS				
LxxCR	9.81E-42	7	30	HPRD_01164/756-761 HPRD_01203/120-125 HPRD_01284/10-15 HPRD_01382/765-770 HPRD_01681/689-694 HPRD_03100/422-427 HPRD_03260/769-774 HPRD_04144/93-98 HPRD_06090/10-15
LxPxP	7.91E-41	14	30	HPRD_00305/2-7 HPRD_00745/136-141 HPRD_00975/331-336 HPRD_01045/85-90 HPRD_01164/1347-1352 HPRD_01203/2062-2067 HPRD_01284/605-610 HPRD_01381/306-311 HPRD_01382/444-449 HPRD_01469/10-15 HPRD_01681/1086-1091 HPRD_02235/159-164 HPRD_03260/91-96 HPRD_03371/260-265 HPRD_05813/304-309 HPRD_05869/427-432 HPRD_09540/436-441
>HPRD_01818 HLH Pfam:Myc_N				
LxQxGS	4.59E-44	4	37	HPRD_00218/1417-1423 HPRD_03221/452-458 HPRD_03360/28-34 HPRD_03938/363-369 HPRD_04380/410-416
GxxxExGE	2.81E-40	5	37	HPRD_01851/409-417 HPRD_03221/223-231 HPRD_03245/460-468 HPRD_03958/409-417 HPRD_03959/409-417 HPRD_03960/409-417 HPRD_04380/182-190 HPRD_04968/253-261 HPRD_05763/409-417
MExxxF	1.83E-39	6	37	HPRD_00296/0-6 HPRD_01801/23-29 HPRD_01851/412-418 HPRD_02820/159-165 HPRD_03587/582-588 HPRD_03958/412-418 HPRD_03959/412-418 HPRD_03960/412-418 HPRD_04310/1308-1314 HPRD_04380/105-111 HPRD_05763/412-418
>HPRD_01819 SH2 SH3 TyrKc				
PxPxP	0.00E+00	30	79	HPRD_00610/44-49 HPRD_00645/15-20 HPRD_00675/42-47 HPRD_00745/135-140 HPRD_00800/288-293 HPRD_01000/417-422 HPRD_01281/1032-1037 HPRD_01320/531-536 HPRD_01381/84-89 HPRD_02314/160-165 HPRD_02433/27-32 HPRD_02545/860-865 HPRD_02563/215-220 HPRD_02635/51-56 HPRD_02641/13-18 HPRD_02756/786-791 HPRD_02952/971-976 HPRD_03131/712-717 HPRD_03139/265-270 HPRD_03203/250-255 HPRD_03301/303-308 HPRD_03926/80-85 HPRD_03937/45-50 HPRD_04108/227-232 HPRD_04242/27-32 HPRD_04382/8-13 HPRD_04468/331-336 HPRD_05026/347-352 HPRD_05599/134-139 HPRD_05667/137-142 HPRD_05674/365-370 HPRD_05866/350-355 HPRD_06571/306-311 HPRD_06577/474-479 HPRD_10227/430-435 HPRD_10228/329-334
PxPxxxP	0.00E+00	31	79	HPRD_00171/275-282 HPRD_00182/466-473 HPRD_00579/1116-1123 HPRD_00610/30-37 HPRD_00645/119-126 HPRD_00698/1147-1154 HPRD_00745/137-144 HPRD_00800/288-295 HPRD_01000/419-426 HPRD_01266/950-957 HPRD_01281/1149-1156 HPRD_01320/544-551 HPRD_01381/84-91 HPRD_01868/814-821 HPRD_02314/215-222 HPRD_02433/27-34 HPRD_02545/108-115 HPRD_02563/209-216 HPRD_02641/13-20 HPRD_02859/663-670 HPRD_02952/376-383 HPRD_02997/845-852 HPRD_03131/644-651 HPRD_03139/287-294 HPRD_03203/250-257 HPRD_03301/724-731 HPRD_03926/62-69 HPRD_03937/45-52 HPRD_04092/850-857 HPRD_04108/229-236 HPRD_04242/11-18 HPRD_04382/8-15 HPRD_04468/333-340 HPRD_05026/335-342 HPRD_05599/136-143 HPRD_05667/96-103 HPRD_05674/365-372 HPRD_05866/623-630 HPRD_06577/474-481 HPRD_10227/424-431 HPRD_10228/247-254
PPxxxxP	0.00E+00	28	79	HPRD_00171/841-848 HPRD_00182/141-148 HPRD_00188/245-252 HPRD_00610/24-31 HPRD_00645/121-128 HPRD_00745/134-141 HPRD_00800/288-295 HPRD_01281/1208-1215 HPRD_01320/544-551 HPRD_01381/123-130 HPRD_02314/174-181 HPRD_02433/33-40 HPRD_02563/209-216 HPRD_02635/105-112 HPRD_02641/12-19 HPRD_02859/733-740 HPRD_02952/972-979 HPRD_02997/845-852 HPRD_03131/713-720 HPRD_03139/221-228 HPRD_03203/34-41 HPRD_03926/62-69 HPRD_03937/45-52 HPRD_04092/756-763 HPRD_04094/241-248 HPRD_04108/231-238 HPRD_04242/7-14 HPRD_04382/8-15 HPRD_04468/564-571 HPRD_05026/341-348 HPRD_05599/513-520 HPRD_05674/765-772 HPRD_05866/351-358 HPRD_06571/307-314 HPRD_06577/720-727 HPRD_10227/458-465 HPRD_10228/753-760
>HPRD_01859 Pfam:P53				
VxxCxxI	1.92E-64	8	110	HPRD_00530/487-494 HPRD_00596/613-620 HPRD_00883/101-108 HPRD_02534/1839-1846 HPRD_02941/626-633 HPRD_03404/1714-1721 HPRD_04078/1802-1809 HPRD_04517/220-227 HPRD_04816/300-307 HPRD_05055/497-504 HPRD_05211/1026-1033 HPRD_05221/54-61 HPRD_06347/1160-1167
SxLE	4.49E-61	26	110	HPRD_00218/783-787 HPRD_00399/540-544 HPRD_00536/1376-1380 HPRD_00537/1338-1342 HPRD_00679/533-537 HPRD_00774/311-315 HPRD_00883/882-886 HPRD_00969/268-272 HPRD_01435/231-235 HPRD_01505/1-5 HPRD_01557/25-29 HPRD_01807/494-498 HPRD_02416/481-485 HPRD_02436/1407-1411 HPRD_02511/49-53 HPRD_02554/537-541 HPRD_02770/252-256 HPRD_02811/668-672 HPRD_02941/313-317 HPRD_03100/413-417 HPRD_03183/98-102 HPRD_03229/299-303 HPRD_03364/251-255 HPRD_03404/1400-1404 HPRD_03479/109-113 HPRD_03540/375-379 HPRD_04114/148-152 HPRD_04207/450-454 HPRD_04395/59-63 HPRD_04517/580-584 HPRD_04633/138-142 HPRD_04816/248-252 HPRD_05055/17-21 HPRD_05211/777-781 HPRD_05212/272-276 HPRD_05361/29-33 HPRD_05364/710-714 HPRD_05395/393-397 HPRD_05569/1712-1716 HPRD_06163/135-139 HPRD_06317/1584-1588 HPRD_06347/469-473 HPRD_06418/112-116

VxxxLK	5.26E-58	19	110	HPRD_00023/610-616 HPRD_00218/82-88 HPRD_00536/270-276 HPRD_00537/1030-1036 HPRD_00561/420-426 HPRD_00593/165-171 HPRD_00596/843-849 HPRD_00777/185-191 HPRD_00883/1313-1319 HPRD_01235/232-238 HPRD_01809/279-285 HPRD_02416/711-717 HPRD_02436/656-662 HPRD_02534/428-434 HPRD_02554/2651-2657 HPRD_02599/297-303 HPRD_02811/290-296 HPRD_02941/568-574 HPRD_03431/215-221 HPRD_03950/592-598 HPRD_04078/412-418 HPRD_04252/236-242 HPRD_04395/123-129 HPRD_05055/1305-1311 HPRD_05212/280-286 HPRD_05362/129-135 HPRD_05363/95-101 HPRD_05395/414-420 HPRD_06317/980-986 HPRD_06347/681-687 HPRD_09875/569-575
>HPRD_01871 RRM ZnF_C3H1				
SRSRxR	2.37E-53	5	6	HPRD_01872/29-35 HPRD_02068/419-425 HPRD_02887/204-210 HPRD_02888/127-133 HPRD_09185/222-228
SxxRSRSR	7.38E-52	5	6	HPRD_01872/27-35 HPRD_02068/438-446 HPRD_02887/200-208 HPRD_02888/127-135 HPRD_09185/220-228
SxSRxRSR	1.24E-51	5	6	HPRD_01872/27-35 HPRD_02068/438-446 HPRD_02887/204-212 HPRD_02888/127-135 HPRD_09185/220-228
>HPRD_02301 COIL SH3				
PxPxP	2.06E-43	10	15	HPRD_01320/531-536 HPRD_01381/84-89 HPRD_01423/1023-1028 HPRD_01681/650-655 HPRD_01750/281-286 HPRD_03786/536-541 HPRD_05136/491-496 HPRD_05153/241-246 HPRD_05428/200-205 HPRD_06369/294-299 HPRD_07338/273-278
PPxxxPR	1.00E-41	5	15	HPRD_01320/822-829 HPRD_01381/83-90 HPRD_01681/1149-1156 HPRD_01750/325-332 HPRD_05136/904-911 HPRD_05428/179-186
PxxxPR	2.82E-41	8	15	HPRD_01320/493-499 HPRD_01381/84-90 HPRD_01681/1020-1026 HPRD_01750/326-332 HPRD_03786/1204-1210 HPRD_05136/504-510 HPRD_05153/43-49 HPRD_05428/180-186 HPRD_06369/294-300 HPRD_07338/444-450
>HPRD_02314 PBD WH1 WH2				
PPxP	4.98E-45	7	16	HPRD_00309/178-182 HPRD_01381/83-87 HPRD_01746/157-161 HPRD_02248/188-192 HPRD_02786/157-161 HPRD_03362/185-189 HPRD_03415/117-121 HPRD_03835/3-7 HPRD_04107/354-358 HPRD_05142/432-436 HPRD_05891/277-281
LPPxP	1.23E-42	4	16	HPRD_01381/306-311 HPRD_01746/156-161 HPRD_02248/187-192 HPRD_02786/156-161 HPRD_03362/196-201 HPRD_03835/251-256 HPRD_04107/391-396
PxPPxP	1.28E-40	4	16	HPRD_01381/88-94 HPRD_01746/155-161 HPRD_02248/186-192 HPRD_02786/167-173 HPRD_03362/405-411 HPRD_03415/170-176 HPRD_03835/1-7 HPRD_04107/354-360
>HPRD_02436 BROMO COIL SIGNAL				
YxSP	3.40E-38	5	23	HPRD_00312/512-516 HPRD_01275/29-33 HPRD_01574/605-609 HPRD_01576/669-673 HPRD_02511/138-142 HPRD_06254/56-60 HPRD_07060/551-555
>HPRD_02437 HOLI Pfam:Androgen_recep ZnF_C4				
SPxxxxP	1.02E-61	17	64	HPRD_00307/100-107 HPRD_00645/431-438 HPRD_01265/295-302 HPRD_01503/156-163 HPRD_01574/779-786 HPRD_02060/1337-1344 HPRD_02172/1060-1067 HPRD_02534/850-857 HPRD_02911/157-164 HPRD_03253/237-244 HPRD_03318/214-221 HPRD_03570/474-481 HPRD_03780/371-378 HPRD_03801/138-145 HPRD_03831/50-57 HPRD_04070/998-1005 HPRD_04380/207-214 HPRD_04556/522-529 HPRD_04992/260-267 HPRD_05055/847-854 HPRD_05349/1231-1238 HPRD_06426/201-208 HPRD_09455/64-71 HPRD_10144/416-423
KxxxSP	7.87E-61	13	64	HPRD_00218/109-115 HPRD_00286/241-247 HPRD_00455/237-243 HPRD_00645/244-250 HPRD_01574/790-796 HPRD_02534/1202-1208 HPRD_02911/1467-1473 HPRD_03143/341-347 HPRD_03253/233-239 HPRD_03318/210-216 HPRD_03570/556-562 HPRD_03801/229-235 HPRD_04070/17-23 HPRD_04556/204-210 HPRD_04992/1220-1226 HPRD_05055/222-228 HPRD_06910/157-163
SPxxxxS	4.62E-60	22	64	HPRD_00026/726-733 HPRD_00218/1496-1503 HPRD_00307/341-348 HPRD_00455/102-109 HPRD_00645/40-47 HPRD_00679/403-410 HPRD_01238/892-899 HPRD_01250/363-370 HPRD_01265/243-250 HPRD_01302/242-249 HPRD_01346/218-225 HPRD_01574/611-618 HPRD_02060/965-972 HPRD_02172/45-52 HPRD_02534/1916-1923 HPRD_02911/96-103 HPRD_02995/137-144 HPRD_03570/492-499 HPRD_03780/91-98 HPRD_04070/516-523 HPRD_04380/415-422 HPRD_04556/109-116 HPRD_04835/237-244 HPRD_04992/49-56 HPRD_05055/1020-1027 HPRD_05349/1307-1314 HPRD_06426/247-254 HPRD_09455/751-758
>HPRD_02511 COIL Pfam:TBP				
LLQ	1.53E-49	15	48	HPRD_00179/286-289 HPRD_00303/254-257 HPRD_00679/594-597 HPRD_00883/325-328 HPRD_01241/426-429 HPRD_01250/121-124 HPRD_01251/105-108 HPRD_02436/759-762 HPRD_02596/117-120 HPRD_02747/36-39 HPRD_03570/116-119 HPRD_04070/692-695 HPRD_04146/203-206 HPRD_05140/51-54 HPRD_05361/154-157 HPRD_05362/184-187 HPRD_05364/149-152 HPRD_05464/34-37 HPRD_05546/110-113 HPRD_06115/299-302 HPRD_08915/1069-1072 HPRD_09696/757-760
SxxxxSxS	7.89E-49	19	48	HPRD_00179/301-309 HPRD_00679/46-54 HPRD_00778/243-251 HPRD_00883/458-466 HPRD_01250/66-74 HPRD_01251/373-381 HPRD_01275/13-21 HPRD_01286/266-274 HPRD_01302/148-156 HPRD_01799/353-361 HPRD_01800/25-33 HPRD_01859/370-378 HPRD_02259/15-23 HPRD_02436/203-211 HPRD_02596/67-75 HPRD_02737/9-17 HPRD_02814/744-752 HPRD_03137/64-72 HPRD_03570/406-414 HPRD_04070/1-9 HPRD_04146/27-35 HPRD_04318/383-391 HPRD_05140/286-294 HPRD_05546/1471-1479 HPRD_06167/296-304 HPRD_08915/1402-1410 HPRD_09696/202-210 HPRD_09897/107-115
QxxxxLQ	1.47E-47	10	48	HPRD_00589/115-122 HPRD_01105/87-94 HPRD_01250/117-124 HPRD_01251/103-110 HPRD_02436/125-132 HPRD_02596/821-828 HPRD_03570/971-978 HPRD_04070/1052-1059 HPRD_05140/478-485 HPRD_05464/30-37 HPRD_05546/647-654 HPRD_06167/218-225 HPRD_09696/124-131
>HPRD_02534 BROMO COIL Pfam:DUF906 Pfam:KIX ZnF_TAZ ZnF_ZZ				
SPxxxxP	0.00E+00	24	65	HPRD_00023/39-46 HPRD_00179/32-39 HPRD_00295/520-527 HPRD_00307/100-107 HPRD_00645/431-438 HPRD_00800/303-310 HPRD_00866/141-148 HPRD_00918/133-140 HPRD_01162/692-699 HPRD_01256/465-472 HPRD_01310/337-344 HPRD_01518/69-76 HPRD_01810/462-469 HPRD_02146/70-77 HPRD_02534/850-857 HPRD_02622/59-66 HPRD_02726/100-107 HPRD_02730/147-154 HPRD_02732/350-357 HPRD_02777/531-538 HPRD_02778/545-552 HPRD_03012/493-500 HPRD_03769/125-132 HPRD_03926/57-64 HPRD_04070/998-1005 HPRD_04212/302-309 HPRD_04517/509-516 HPRD_04769/172-179 HPRD_05665/473-480 HPRD_07567/762-769 HPRD_10144/416-423
PxxPxxxG	0.00E+00	23	65	HPRD_00179/47-55 HPRD_00215/166-174 HPRD_00295/1221-1229 HPRD_00549/237-245 HPRD_00592/335-343 HPRD_00645/121-129 HPRD_00800/415-423 HPRD_00866/102-110 HPRD_00883/71-79 HPRD_01162/9-17 HPRD_01166/182-190 HPRD_01810/354-362 HPRD_01859/218-226 HPRD_02146/111-119 HPRD_02534/769-777 HPRD_02612/464-472 HPRD_02622/34-42 HPRD_03012/650-658 HPRD_03402/363-371 HPRD_03430/43-51 HPRD_03769/205-213 HPRD_03926/295-303 HPRD_04070/250-258 HPRD_04212/69-77 HPRD_04232/157-165 HPRD_05002/46-54 HPRD_09398/233-241 HPRD_10144/843-851

PxxxGP	0.00E+00	22	65	HPRD_00023/519-525 HPRD_00179/58-64 HPRD_00277/360-366 HPRD_00592/338-344 HPRD_00645/124-130 HPRD_00800/289-295 HPRD_00883/74-80 HPRD_00918/249-255 HPRD_01162/1307-1313 HPRD_01302/19-25 HPRD_02070/327-333 HPRD_02092/616-622 HPRD_02146/114-120 HPRD_02622/141-147 HPRD_02730/88-94 HPRD_03012/436-442 HPRD_03402/612-618 HPRD_03430/170-176 HPRD_03769/126-132 HPRD_03926/68-74 HPRD_04129/255-261 HPRD_04212/72-78 HPRD_04647/135-141 HPRD_05665/474-480 HPRD_06039/1144-1150 HPRD_07567/763-769 HPRD_10144/832-838
>HPRD_02612 HOLI ZnF_C4				
QxRLQxxQ	3.49E-46	4	13	HPRD_02534/1855-1863 HPRD_04070/1052-1060 HPRD_04556/466-474 HPRD_09066/1193-1201
QxxxxxSP	6.17E-45	8	13	HPRD_00645/280-288 HPRD_00800/480-488 HPRD_02534/441-449 HPRD_03221/238-246 HPRD_03253/67-75 HPRD_04070/437-445 HPRD_05055/668-676 HPRD_09066/480-488
MxPxQ	3.54E-43	6	13	HPRD_00584/160-165 HPRD_00800/476-481 HPRD_02172/0-5 HPRD_02534/876-881 HPRD_04070/1037-1042 HPRD_09066/476-481
>HPRD_02646 TUDOR				
RGxGG	2.52E-44	5	19	HPRD_01655/131-136 HPRD_04386/1239-1244 HPRD_06228/448-453 HPRD_06284/87-92 HPRD_06734/445-450 HPRD_07569/12-17
GxxGxxRG	9.96E-44	6	19	HPRD_01655/125-133 HPRD_02605/410-418 HPRD_04386/1210-1218 HPRD_06284/102-110 HPRD_06734/526-534 HPRD_07569/6-14 HPRD_07600/16-24
GRGxxG	2.48E-41	5	19	HPRD_02605/348-354 HPRD_04386/1249-1255 HPRD_06228/447-453 HPRD_06284/86-92 HPRD_06734/444-450 HPRD_07569/44-50
>HPRD_02737 Pfam:PC4*				
TPQP	6.09E-38	4	9	HPRD_02436/1663-1667 HPRD_02511/38-42 HPRD_02612/461-465 HPRD_04078/1867-1871
>HPRD_02740 SH2 SH3				
PPxPP	4.30E-73	13	36	HPRD_00610/44-49 HPRD_00745/134-139 HPRD_01299/198-203 HPRD_01681/1150-1155 HPRD_01809/898-903 HPRD_02314/313-318 HPRD_03362/185-190 HPRD_03835/5-10 HPRD_03926/294-299 HPRD_03937/46-51 HPRD_03972/574-579 HPRD_03995/11-16 HPRD_04336/180-185 HPRD_05428/10-15 HPRD_05448/276-281
PPxxP	6.71E-72	18	36	HPRD_00391/746-751 HPRD_00541/369-374 HPRD_00579/384-389 HPRD_00610/44-49 HPRD_00745/134-139 HPRD_01299/198-203 HPRD_01681/923-928 HPRD_01809/898-903 HPRD_02314/160-165 HPRD_03362/185-190 HPRD_03835/4-9 HPRD_03851/788-793 HPRD_03926/63-68 HPRD_03937/45-50 HPRD_03972/573-578 HPRD_03995/11-16 HPRD_04248/77-82 HPRD_04336/180-185 HPRD_05153/43-48 HPRD_05235/696-701 HPRD_05411/278-283 HPRD_05428/10-15 HPRD_05448/150-155 HPRD_06104/603-608
PxxPP	1.48E-71	17	36	HPRD_00391/621-626 HPRD_00541/282-287 HPRD_00610/21-26 HPRD_00745/134-139 HPRD_01299/20-25 HPRD_01681/1085-1090 HPRD_01809/896-901 HPRD_02314/311-316 HPRD_03362/182-187 HPRD_03835/1-6 HPRD_03851/752-757 HPRD_03926/64-69 HPRD_03937/46-51 HPRD_03972/571-576 HPRD_03995/11-16 HPRD_04248/352-357 HPRD_04336/180-185 HPRD_05153/161-166 HPRD_05235/245-250 HPRD_05428/10-15 HPRD_05448/276-281 HPRD_06104/489-494
>HPRD_02780 PTB SH2				
PxxPxxP	0.00E+00	25	68	HPRD_00104/36-43 HPRD_00138/3286-3293 HPRD_00179/58-65 HPRD_00546/861-868 HPRD_00587/442-449 HPRD_00634/801-808 HPRD_00737/656-663 HPRD_00896/470-477 HPRD_00943/886-893 HPRD_00946/1326-1333 HPRD_00975/621-628 HPRD_00990/468-475 HPRD_01044/773-780 HPRD_01281/1070-1077 HPRD_01320/544-551 HPRD_01381/304-311 HPRD_01470/558-565 HPRD_01477/306-313 HPRD_01495/267-274 HPRD_01820/1141-1148 HPRD_01868/73-80 HPRD_02513/768-775 HPRD_02563/206-213 HPRD_02627/232-239 HPRD_02678/380-387 HPRD_02859/663-670 HPRD_02878/461-468 HPRD_02900/152-159 HPRD_03016/939-946 HPRD_03131/18-25 HPRD_03583/394-401 HPRD_03596/102-109 HPRD_04404/257-264 HPRD_05136/555-562 HPRD_06256/667-674
PPxP	0.00E+00	27	68	HPRD_00100/483-487 HPRD_00104/1441-1445 HPRD_00179/437-441 HPRD_00546/303-307 HPRD_00587/445-449 HPRD_00737/450-454 HPRD_00739/132-136 HPRD_00896/473-477 HPRD_00943/154-158 HPRD_00990/691-695 HPRD_00993/499-503 HPRD_01044/268-272 HPRD_01052/359-363 HPRD_01280/1299-1303 HPRD_01281/1148-1152 HPRD_01320/480-484 HPRD_01381/83-87 HPRD_01423/36-40 HPRD_01477/306-310 HPRD_01820/1207-1211 HPRD_02513/333-337 HPRD_02545/4-8 HPRD_02563/209-213 HPRD_02627/284-288 HPRD_02678/380-384 HPRD_02859/733-737 HPRD_02878/8-12 HPRD_02900/138-142 HPRD_02971/298-302 HPRD_03016/602-606 HPRD_03075/672-676 HPRD_03131/713-717 HPRD_03583/394-398 HPRD_04404/257-261 HPRD_05136/504-508 HPRD_05772/257-261 HPRD_06256/269-273 HPRD_08923/180-184
PPxxP	2.12E-71	26	68	HPRD_00104/35-40 HPRD_00138/1146-1151 HPRD_00179/398-403 HPRD_00546/827-832 HPRD_00579/384-389 HPRD_00587/26-31 HPRD_00737/121-126 HPRD_00739/547-552 HPRD_00932/632-637 HPRD_00943/1200-1205 HPRD_00975/643-648 HPRD_00990/470-475 HPRD_01044/268-273 HPRD_01320/493-498 HPRD_01381/307-312 HPRD_01470/560-565 HPRD_01477/307-312 HPRD_01494/327-332 HPRD_01495/560-565 HPRD_01501/617-622 HPRD_01820/1207-1212 HPRD_02513/332-337 HPRD_02545/860-865 HPRD_02563/211-216 HPRD_02627/284-289 HPRD_02678/386-391 HPRD_02859/665-670 HPRD_02878/460-465 HPRD_02900/138-143 HPRD_03016/667-672 HPRD_03131/712-717 HPRD_03583/310-315 HPRD_04404/210-215 HPRD_05136/504-509 HPRD_05156/189-194 HPRD_05772/256-261 HPRD_06256/268-273 HPRD_08923/179-184
>HPRD_02807 MADS				
QxxxxPQ	5.14E-38	6	12	HPRD_00011/14-21 HPRD_02807/429-436 HPRD_02810/364-371 HPRD_02995/238-245 HPRD_04078/478-485 HPRD_05610/465-472 HPRD_05944/452-459 HPRD_07185/433-440
>HPRD_02859 B41 Pfam:Focal_AT TyrKc				
GQxxC	2.11E-50	5	42	HPRD_00628/533-538 HPRD_01428/516-521 HPRD_01439/1093-1098 HPRD_01494/257-262 HPRD_01726/1358-1363 HPRD_01903/180-185 HPRD_02506/514-519 HPRD_03937/414-419
VxPxxS	3.39E-47	13	42	HPRD_00579/1199-1205 HPRD_00628/191-197 HPRD_00655/137-143 HPRD_00943/618-624 HPRD_01281/603-609 HPRD_01288/132-138 HPRD_01381/96-102 HPRD_01439/829-835 HPRD_01726/423-429 HPRD_01740/385-391 HPRD_01819/139-145 HPRD_01903/755-761 HPRD_03105/1596-1602 HPRD_03203/2898-2904 HPRD_03431/364-370 HPRD_03937/48-54 HPRD_04248/307-313 HPRD_05809/38-44 HPRD_06577/98-104
VAPxxS	1.03E-43	4	42	HPRD_00579/1199-1205 HPRD_00655/137-143 HPRD_00943/618-624 HPRD_01288/132-138 HPRD_01381/96-102 HPRD_01726/722-728 HPRD_01819/139-145
>HPRD_02887 RRM				
SRSxxRxR	5.52E-50	6	12	HPRD_01617/256-264 HPRD_01871/194-202 HPRD_02068/419-427 HPRD_03599/248-256 HPRD_04720/591-599 HPRD_09087/185-193
RSRxxxR	1.32E-46	6	12	HPRD_01617/255-262 HPRD_01871/193-200 HPRD_02068/420-427 HPRD_03599/249-256 HPRD_04720/618-625 HPRD_09087/305-312

SRSxxR	1.56E-46	6	12	HPRD_01617/256-262 HPRD_01871/194-200 HPRD_02068/419-425 HPRD_03599/248-254 HPRD_04720/548-554 HPRD_09087/185-191
>HPRD_02888 RRM				
SRSxxRxR	6.30E-51	6	8	HPRD_01617/256-264 HPRD_01871/194-202 HPRD_02068/419-427 HPRD_03599/248-256 HPRD_04720/591-599 HPRD_09185/222-230
RxxSRSR	1.85E-50	6	8	HPRD_01617/253-260 HPRD_01871/193-200 HPRD_02068/416-423 HPRD_03599/245-252 HPRD_04720/614-621 HPRD_09185/223-230
SRSRxxxR	7.79E-49	6	8	HPRD_01617/256-264 HPRD_01871/194-202 HPRD_02068/419-427 HPRD_03599/248-256 HPRD_04720/617-625 HPRD_09185/222-230
>HPRD_02910 COIL SANT				
SxxxSxxS	1.26E-43	16	24	HPRD_00180/342-350 HPRD_00590/97-105 HPRD_00679/389-397 HPRD_00744/78-86 HPRD_00920/457-465 HPRD_01166/196-204 HPRD_01238/902-910 HPRD_01241/356-364 HPRD_01250/73-81 HPRD_01275/13-21 HPRD_02679/45-53 HPRD_02788/308-316 HPRD_03247/430-438 HPRD_03570/526-534 HPRD_04340/28-36 HPRD_04721/404-412 HPRD_05568/256-264 HPRD_05610/214-222 HPRD_10230/239-247
SxxxxxSP	3.82E-42	11	24	HPRD_00023/29-37 HPRD_00180/336-344 HPRD_00590/270-278 HPRD_00744/88-96 HPRD_01166/293-301 HPRD_01250/357-365 HPRD_01275/14-22 HPRD_03301/773-781 HPRD_03570/473-481 HPRD_04721/69-77 HPRD_05568/358-366 HPRD_05610/208-216 HPRD_10230/247-255
SPxxxxS	4.91E-42	11	24	HPRD_00180/249-256 HPRD_00590/91-98 HPRD_00679/403-410 HPRD_00744/133-140 HPRD_01166/200-207 HPRD_01238/892-899 HPRD_01250/363-370 HPRD_01275/31-38 HPRD_02788/310-317 HPRD_03247/420-427 HPRD_03301/192-199 HPRD_03570/492-499 HPRD_04721/84-91 HPRD_05037/192-199 HPRD_05568/364-371 HPRD_10230/243-250
>HPRD_02911 COIL SANT				
RxGRxxK	5.75E-40	4	19	HPRD_00589/259-266 HPRD_02050/249-256 HPRD_03809/174-181 HPRD_03873/203-210 HPRD_09830/317-324 HPRD_10230/1948-1955
>HPRD_02995 DWA DWB				
LSP	1.20E-59	16	35	HPRD_00312/638-641 HPRD_00443/110-113 HPRD_00645/327-330 HPRD_01075/130-133 HPRD_01271/392-395 HPRD_01302/241-244 HPRD_01304/257-260 HPRD_01313/221-224 HPRD_02070/266-269 HPRD_02146/135-138 HPRD_02437/256-259 HPRD_02439/540-543 HPRD_02807/179-182 HPRD_03221/248-251 HPRD_03672/180-183 HPRD_04061/10-13 HPRD_04380/206-209 HPRD_06090/855-858 HPRD_06113/313-316 HPRD_06207/282-285 HPRD_06999/33-36 HPRD_09273/266-269
SxxxSP	6.09E-57	16	35	HPRD_00312/744-750 HPRD_00443/441-447 HPRD_00645/36-42 HPRD_01271/496-502 HPRD_01304/250-256 HPRD_01313/205-211 HPRD_01796/68-74 HPRD_01801/226-232 HPRD_02070/263-269 HPRD_02146/62-68 HPRD_02437/646-652 HPRD_02439/241-247 HPRD_02807/160-166 HPRD_03356/190-196 HPRD_04061/279-285 HPRD_04380/203-209 HPRD_05189/989-995 HPRD_06090/655-661 HPRD_06113/175-181 HPRD_06207/343-349 HPRD_09273/147-153
SSxS	4.55E-53	20	35	HPRD_00443/177-181 HPRD_00645/151-155 HPRD_00856/132-136 HPRD_01075/108-112 HPRD_01113/140-144 HPRD_01271/26-30 HPRD_01303/185-189 HPRD_01313/208-212 HPRD_01319/95-99 HPRD_01796/44-48 HPRD_01801/219-223 HPRD_02070/260-264 HPRD_02146/63-67 HPRD_02437/174-178 HPRD_02439/106-110 HPRD_02807/458-462 HPRD_03221/463-467 HPRD_03356/197-201 HPRD_04061/349-353 HPRD_04380/421-425 HPRD_04596/359-363 HPRD_05189/44-48 HPRD_06090/260-264 HPRD_06113/521-525 HPRD_06207/1027-1031 HPRD_06888/250-254 HPRD_06948/851-855 HPRD_09273/146-150
>HPRD_03100 S_TKc				
RxxSxES	1.19E-42	7	26	HPRD_00443/303-310 HPRD_01139/537-544 HPRD_03213/76-83 HPRD_04011/371-378 HPRD_04309/53-60 HPRD_05223/469-476 HPRD_09674/585-592
RxxSxE	3.36E-38	11	26	HPRD_00443/303-309 HPRD_00943/257-263 HPRD_01071/473-479 HPRD_01139/537-543 HPRD_02941/1183-1189 HPRD_03213/76-82 HPRD_04011/371-377 HPRD_04309/53-59 HPRD_05223/469-475 HPRD_09247/274-280 HPRD_09525/504-510 HPRD_09674/242-248
>HPRD_03109 RAN				
LxxxLQ	3.67E-44	10	19	HPRD_01809/24-30 HPRD_03111/2146-2152 HPRD_03217/506-512 HPRD_03597/235-241 HPRD_03975/896-902 HPRD_04114/448-454 HPRD_04128/1170-1176 HPRD_04210/11-17 HPRD_04418/82-88 HPRD_04820/272-278 HPRD_06385/192-198 HPRD_10003/477-483
QxxxxLL	8.73E-42	10	19	HPRD_01559/217-224 HPRD_01809/1111-1118 HPRD_02437/875-882 HPRD_03111/252-259 HPRD_03217/39-46 HPRD_03597/25-32 HPRD_03975/894-901 HPRD_04128/252-259 HPRD_04210/12-19 HPRD_04418/416-423 HPRD_04820/196-203 HPRD_04835/198-205 HPRD_06385/1049-1056 HPRD_06407/131-138 HPRD_10003/689-696
LxxxLC	2.25E-39	7	19	HPRD_03217/388-394 HPRD_03597/278-284 HPRD_03975/321-327 HPRD_04114/579-585 HPRD_04210/128-134 HPRD_04418/262-268 HPRD_04835/375-381 HPRD_06385/249-255 HPRD_09280/145-151
>HPRD_03131 B41 Pfam:Focal_AT TyrKc				
PPxxxxxP	7.13E-49	15	42	HPRD_00579/264-272 HPRD_00592/72-80 HPRD_00745/137-145 HPRD_00993/719-727 HPRD_01267/71-79 HPRD_01281/1148-1156 HPRD_01284/607-615 HPRD_01320/496-504 HPRD_01381/83-91 HPRD_01428/194-202 HPRD_01470/560-568 HPRD_01726/2164-2172 HPRD_02496/36-44 HPRD_02513/355-363 HPRD_02780/4-12 HPRD_03831/17-25 HPRD_03937/45-53 HPRD_04199/16-24 HPRD_04248/77-85 HPRD_04820/771-779 HPRD_07497/293-301 HPRD_07498/9-17 HPRD_08335/887-895 HPRD_09932/103-111
RPLP	1.00E-44	6	42	HPRD_01320/562-566 HPRD_01381/92-96 HPRD_01443/137-141 HPRD_04248/634-638 HPRD_07498/335-339 HPRD_09932/304-308
PxPP	3.65E-43	14	42	HPRD_00579/933-937 HPRD_00592/554-558 HPRD_00745/135-139 HPRD_00992/1102-1106 HPRD_01267/66-70 HPRD_01281/941-945 HPRD_01284/606-610 HPRD_01320/44-48 HPRD_01381/88-92 HPRD_01470/558-562 HPRD_01726/2162-2166 HPRD_01819/484-488 HPRD_02177/356-360 HPRD_02513/334-338 HPRD_02547/34-38 HPRD_02780/410-414 HPRD_03831/175-179 HPRD_03937/47-51 HPRD_04248/91-95 HPRD_04820/785-789 HPRD_07497/480-484 HPRD_07498/931-935 HPRD_08335/476-480 HPRD_09932/118-122
>HPRD_03143 Pfam:Hist_deacetyl				
SxxxxxSS	0.00E+00	32	66	HPRD_00023/213-221 HPRD_00024/295-303 HPRD_00139/368-376 HPRD_00218/281-289 HPRD_00312/642-650 HPRD_00532/763-771 HPRD_01166/193-201 HPRD_01203/875-883 HPRD_01241/514-522 HPRD_01272/394-402 HPRD_01283/335-343 HPRD_01310/321-329 HPRD_01574/787-795 HPRD_01575/268-276 HPRD_01576/964-972 HPRD_01796/39-47 HPRD_02326/31-39 HPRD_02437/425-433 HPRD_02487/49-57 HPRD_02806/300-308 HPRD_02886/205-213 HPRD_03221/457-465 HPRD_03477/267-275 HPRD_03483/159-167 HPRD_03647/116-124 HPRD_03668/27-35 HPRD_03742/144-152 HPRD_03927/134-142 HPRD_04141/6-14 HPRD_04256/46-54 HPRD_04424/580-588 HPRD_04633/554-562 HPRD_04788/36-44 HPRD_04826/398-406 HPRD_04847/148-156 HPRD_05243/138-146 HPRD_05944/272-280 HPRD_05964/307-315 HPRD_06458/764-772 HPRD_06828/109-117 HPRD_09071/1521-1529 HPRD_09691/392-400 HPRD_10230/271-279

PxxPxP	8.95E-81	31	66	HPRD_00023/20-26 HPRD_00139/26-32 HPRD_00312/961-967 HPRD_00532/709-715 HPRD_01166/234-240 HPRD_01203/2757-2763 HPRD_01241/68-74 HPRD_01283/57-63 HPRD_01310/564-570 HPRD_01574/19-25 HPRD_01576/8-14 HPRD_01753/95-101 HPRD_02437/373-379 HPRD_02806/5-11 HPRD_03221/217-223 HPRD_03245/188-194 HPRD_03647/608-614 HPRD_03742/793-799 HPRD_03808/179-185 HPRD_03927/690-696 HPRD_04015/22-28 HPRD_04232/157-163 HPRD_04424/23-29 HPRD_04488/367-373 HPRD_04633/544-550 HPRD_04787/148-154 HPRD_04788/333-339 HPRD_04803/264-270 HPRD_04826/191-197 HPRD_04847/77-83 HPRD_06293/141-147 HPRD_06458/333-339 HPRD_06828/456-462 HPRD_09071/239-245 HPRD_09417/273-279 HPRD_09426/37-43 HPRD_09691/756-762 HPRD_10230/2428-2434
PPxxxP	3.06E-66	28	66	HPRD_00023/23-29 HPRD_00024/84-90 HPRD_00139/26-32 HPRD_00532/878-884 HPRD_01203/1547-1553 HPRD_01241/241-247 HPRD_01283/57-63 HPRD_01310/716-722 HPRD_01574/19-25 HPRD_01575/991-997 HPRD_01576/8-14 HPRD_01753/95-101 HPRD_02437/275-281 HPRD_02806/9-15 HPRD_03221/175-181 HPRD_03245/24-30 HPRD_03342/213-219 HPRD_03647/466-472 HPRD_03742/88-94 HPRD_03808/115-121 HPRD_03927/607-613 HPRD_04015/411-417 HPRD_04141/451-457 HPRD_04256/138-144 HPRD_04633/546-552 HPRD_04826/197-203 HPRD_04847/76-82 HPRD_06293/182-188 HPRD_06458/333-339 HPRD_06828/948-954 HPRD_09071/238-244 HPRD_09691/756-762 HPRD_10230/1633-1639
>HPRD_03183 14_3_3				
SxxxxxSP	8.87E-60	18	44	HPRD_00086/317-325 HPRD_00305/32-40 HPRD_00307/35-43 HPRD_00645/34-42 HPRD_00729/106-114 HPRD_00739/500-508 HPRD_00932/2-10 HPRD_00943/412-420 HPRD_01044/349-357 HPRD_01142/231-239 HPRD_01264/742-750 HPRD_01265/282-290 HPRD_01850/1210-1218 HPRD_01859/120-128 HPRD_02409/88-96 HPRD_02730/229-237 HPRD_02878/431-439 HPRD_03210/295-303 HPRD_03720/290-298 HPRD_03904/979-987 HPRD_04061/348-356 HPRD_04342/405-413 HPRD_05594/288-296 HPRD_05610/208-216 HPRD_05813/118-126 HPRD_09096/206-214 HPRD_10068/215-223
RxRxxS	4.82E-59	13	44	HPRD_00085/239-245 HPRD_00645/250-256 HPRD_00932/959-965 HPRD_00943/264-270 HPRD_01142/525-531 HPRD_01146/445-451 HPRD_01261/240-246 HPRD_01264/358-364 HPRD_01265/253-259 HPRD_01383/460-466 HPRD_01850/933-939 HPRD_02409/147-153 HPRD_02730/270-276 HPRD_02878/300-306 HPRD_03720/832-838 HPRD_03904/77-83 HPRD_03965/160-166 HPRD_04061/247-253 HPRD_04409/69-75
SxSxP	1.71E-57	19	44	HPRD_00307/341-346 HPRD_00645/467-472 HPRD_00739/504-509 HPRD_00932/237-242 HPRD_00943/371-376 HPRD_01044/92-97 HPRD_01146/14-19 HPRD_01264/312-317 HPRD_01265/256-261 HPRD_01850/537-542 HPRD_01857/551-556 HPRD_01859/93-98 HPRD_02409/477-482 HPRD_02878/163-168 HPRD_03239/31-36 HPRD_03904/963-968 HPRD_03965/354-359 HPRD_04061/356-361 HPRD_04248/491-496 HPRD_04342/411-416 HPRD_04409/96-101 HPRD_05594/1070-1075 HPRD_05610/2-7 HPRD_10068/204-209
>HPRD_03184 14_3_3				
SxxxxxSP	4.83E-44	15	28	HPRD_00305/32-40 HPRD_00307/35-43 HPRD_00628/208-216 HPRD_00932/2-10 HPRD_00943/412-420 HPRD_01142/231-239 HPRD_01264/742-750 HPRD_01265/282-290 HPRD_01819/68-76 HPRD_01835/34-42 HPRD_01907/120-128 HPRD_02902/728-736 HPRD_03402/356-364 HPRD_03470/500-508 HPRD_04342/405-413 HPRD_05298/525-533 HPRD_05622/92-100 HPRD_05813/118-126 HPRD_06060/3652-3660 HPRD_06767/537-545 HPRD_09246/497-505
RRxS	1.61E-43	12	28	HPRD_00305/288-292 HPRD_00307/77-81 HPRD_00943/409-413 HPRD_01264/441-445 HPRD_01265/39-43 HPRD_01819/13-17 HPRD_01835/193-197 HPRD_01907/74-78 HPRD_03184/56-60 HPRD_03626/69-73 HPRD_04342/1029-1033 HPRD_04409/114-118 HPRD_05622/416-420 HPRD_05813/287-291 HPRD_06060/2623-2627 HPRD_09246/275-279
RxSS	1.97E-43	12	28	HPRD_00305/187-191 HPRD_00307/247-251 HPRD_00943/326-330 HPRD_01264/360-364 HPRD_01265/335-339 HPRD_01907/208-212 HPRD_02902/722-726 HPRD_03184/56-60 HPRD_03470/36-40 HPRD_03626/276-280 HPRD_04409/71-75 HPRD_05298/342-346 HPRD_05622/431-435 HPRD_05813/288-292 HPRD_06060/2055-2059 HPRD_06767/391-395 HPRD_09246/275-279
>HPRD_03221 DWA DWB				
SxxxSxxS	3.24E-67	23	45	HPRD_00258/934-942 HPRD_00312/564-572 HPRD_01271/493-501 HPRD_01313/71-79 HPRD_01319/107-115 HPRD_01796/40-48 HPRD_01798/277-285 HPRD_01818/202-210 HPRD_02566/9-17 HPRD_02806/306-314 HPRD_02807/452-460 HPRD_02809/105-113 HPRD_03139/305-313 HPRD_03143/112-120 HPRD_03220/205-213 HPRD_04023/117-125 HPRD_04061/310-318 HPRD_04078/544-552 HPRD_04340/28-36 HPRD_04380/417-425 HPRD_04689/163-171 HPRD_04819/320-328 HPRD_04899/184-192 HPRD_05780/319-327 HPRD_05994/153-161 HPRD_06039/168-176 HPRD_06090/674-682 HPRD_09273/142-150
SxxSS	5.61E-67	24	45	HPRD_00258/519-524 HPRD_00286/347-352 HPRD_00304/802-807 HPRD_00975/631-636 HPRD_01271/26-31 HPRD_01313/159-164 HPRD_01319/95-100 HPRD_01796/41-46 HPRD_01798/284-289 HPRD_01818/209-214 HPRD_01919/294-299 HPRD_02566/13-18 HPRD_02806/306-311 HPRD_02807/456-461 HPRD_02809/424-429 HPRD_03139/305-310 HPRD_03143/405-410 HPRD_03220/200-205 HPRD_04023/117-122 HPRD_04061/424-429 HPRD_04340/32-37 HPRD_04587/125-130 HPRD_04782/165-170 HPRD_04819/221-226 HPRD_04899/184-189 HPRD_05780/326-331 HPRD_05994/157-162 HPRD_06039/796-801 HPRD_06090/655-660 HPRD_06901/192-197 HPRD_09273/146-151
SSxS	1.07E-66	25	45	HPRD_00258/539-543 HPRD_00304/218-222 HPRD_00589/574-578 HPRD_00975/783-787 HPRD_01075/108-112 HPRD_01271/26-30 HPRD_01313/208-212 HPRD_01319/95-99 HPRD_01796/44-48 HPRD_01798/311-315 HPRD_01818/218-222 HPRD_02566/414-418 HPRD_02806/306-310 HPRD_02807/458-462 HPRD_02809/330-334 HPRD_03139/323-327 HPRD_03220/234-238 HPRD_04061/349-353 HPRD_04078/796-800 HPRD_04380/421-425 HPRD_04782/130-134 HPRD_04819/224-228 HPRD_04899/496-500 HPRD_05633/371-375 HPRD_05780/788-792 HPRD_05994/219-223 HPRD_06039/98-102 HPRD_06090/260-264 HPRD_06948/851-855 HPRD_09273/146-150
>HPRD_03356 DWA DWB				
YxLxPxxV	2.75E-39	4	20	HPRD_01075/196-204 HPRD_01283/399-407 HPRD_03975/656-664 HPRD_06039/1184-1192
SPxxxxS	1.73E-38	11	20	HPRD_01075/165-172 HPRD_01313/296-303 HPRD_02995/137-144 HPRD_03430/193-200 HPRD_03710/128-135 HPRD_03975/390-397 HPRD_04078/19-26 HPRD_04240/273-280 HPRD_05780/352-359 HPRD_06039/15-22 HPRD_06901/196-203 HPRD_06999/51-58 HPRD_09273/153-160
SxxSSP	2.37E-38	7	20	HPRD_01313/205-211 HPRD_03430/196-202 HPRD_05189/989-995 HPRD_05780/355-361 HPRD_06090/655-661 HPRD_06901/192-198 HPRD_09273/147-153
>HPRD_03538 COIL MATH Pfam:zf-TRAF RING				
PxxExG	7.77E-42	12	47	HPRD_00144/146-152 HPRD_01074/336-342 HPRD_01723/236-242 HPRD_01863/461-467 HPRD_02060/583-589 HPRD_02988/311-317 HPRD_03028/16-22 HPRD_03767/176-182 HPRD_03904/879-885 HPRD_04609/606-612 HPRD_04625/174-180 HPRD_04879/213-219 HPRD_05230/198-204 HPRD_05365/284-290 HPRD_05801/112-118 HPRD_06427/257-263 HPRD_09442/1667-1673
HLxxxxxS	4.60E-38	10	47	HPRD_01862/317-325 HPRD_03419/353-361 HPRD_03426/339-347 HPRD_03538/242-250 HPRD_03737/186-194 HPRD_04625/112-120 HPRD_04671/282-290 HPRD_05230/316-324 HPRD_05322/624-632 HPRD_05365/80-88 HPRD_05463/601-609 HPRD_05811/29-37 HPRD_06048/1081-1089 HPRD_06427/128-136 HPRD_09442/1256-1264
>HPRD_03596 SH2 SH3				
PPxxPP	4.98E-58	8	27	HPRD_01320/542-548 HPRD_01681/1149-1155 HPRD_02314/313-319 HPRD_02627/300-306 HPRD_03583/393-399 HPRD_03937/45-51 HPRD_04248/351-357 HPRD_04404/259-265 HPRD_04620/138-144 HPRD_05116/340-346 HPRD_05136/553-559 HPRD_05374/388-394 HPRD_05866/350-356

PxxPPK	1.88E-57	6	27	HPRD_01320/819-825 HPRD_01681/1302-1308 HPRD_02627/301-307 HPRD_03583/394-400 HPRD_03937/337-343 HPRD_04404/299-305 HPRD_05116/341-347 HPRD_05136/901-907 HPRD_05153/161-167 HPRD_05374/389-395 HPRD_05866/351-357
PxxPPxP	4.24E-57	12	27	HPRD_00587/442-449 HPRD_01267/68-75 HPRD_01320/819-826 HPRD_01681/1147-1154 HPRD_01809/896-903 HPRD_02314/311-318 HPRD_02627/284-291 HPRD_02780/43-50 HPRD_03583/394-401 HPRD_03937/46-53 HPRD_04404/299-306 HPRD_04620/627-634 HPRD_05116/341-348 HPRD_05136/818-825 HPRD_05153/161-168 HPRD_05374/389-396 HPRD_05866/351-358
>HPRD_03599 RRM				
RxRSRxR	1.08E-39	4	5	HPRD_01617/241-248 HPRD_01872/30-37 HPRD_02887/203-210 HPRD_02888/124-131
SRSRxR	1.35E-39	4	5	HPRD_01617/256-262 HPRD_01872/29-35 HPRD_02887/204-210 HPRD_02888/127-133
RxxSRSR	7.80E-39	4	5	HPRD_01617/253-260 HPRD_01872/26-33 HPRD_02887/203-210 HPRD_02888/124-131
>HPRD_03627 RHO				
PPxPxxR	3.18E-48	8	35	HPRD_01381/83-90 HPRD_02130/573-580 HPRD_02314/314-321 HPRD_03240/1827-1834 HPRD_03995/11-18 HPRD_05428/10-17 HPRD_09667/389-396 HPRD_10227/546-553 HPRD_10259/1103-1110
LxFY	1.10E-45	7	35	HPRD_02130/448-452 HPRD_02820/473-477 HPRD_03032/623-627 HPRD_03240/1203-1207 HPRD_03995/127-131 HPRD_04021/9-13 HPRD_05353/413-417 HPRD_05428/126-130 HPRD_05434/147-151 HPRD_05706/479-483 HPRD_06316/168-172 HPRD_09121/1207-1211 HPRD_10138/10-14
PPxPP	1.81E-45	10	35	HPRD_01381/307-312 HPRD_01989/155-160 HPRD_02130/573-578 HPRD_02314/313-318 HPRD_03240/1825-1830 HPRD_03995/11-16 HPRD_05428/10-15 HPRD_05434/277-282 HPRD_06049/582-587 HPRD_09667/389-394 HPRD_10227/506-511 HPRD_10259/1103-1108
>HPRD_03833 COIL MATH Pfam:zf-TRAF RING				
PxxPxExD	5.46E-38	5	30	HPRD_01197/214-222 HPRD_02235/581-589 HPRD_03319/383-391 HPRD_03904/1030-1038 HPRD_05123/474-482
>HPRD_03937 LIM Pfam:Paxillin				
PPxxPxR	6.54E-50	5	34	HPRD_01900/615-622 HPRD_02142/10-17 HPRD_02262/476-483 HPRD_02513/332-339 HPRD_02859/893-900 HPRD_03131/712-719 HPRD_03203/1-8 HPRD_03995/11-18 HPRD_04820/828-835
PPxxxPR	1.10E-44	5	34	HPRD_02142/187-194 HPRD_02262/476-483 HPRD_02513/332-339 HPRD_02859/893-900 HPRD_03131/854-861 HPRD_03995/186-193 HPRD_04820/930-937
PPxxxxR	9.09E-40	8	34	HPRD_00566/435-442 HPRD_01900/615-622 HPRD_02142/10-17 HPRD_02262/476-483 HPRD_02496/35-42 HPRD_02513/332-339 HPRD_02859/893-900 HPRD_03131/712-719 HPRD_03203/1-8 HPRD_03995/11-18 HPRD_04242/125-132 HPRD_04820/828-835 HPRD_05247/457-464 HPRD_06577/480-487
>HPRD_03975 Pfam:IBN_N				
GxFxFG	2.26E-45	4	28	HPRD_00258/2058-2064 HPRD_03111/2858-2864 HPRD_04899/921-927 HPRD_05782/13-19
GFxFG	2.95E-45	4	28	HPRD_00258/1258-1263 HPRD_03111/1857-1862 HPRD_04899/1020-1025 HPRD_05782/2-7
FxFGQ	1.06E-43	4	28	HPRD_00258/1820-1825 HPRD_03111/1456-1461 HPRD_04508/259-264 HPRD_04899/1225-1230
>HPRD_03999 Pfam:SIP1				
SRSRxR	6.89E-42	5	7	HPRD_01617/256-262 HPRD_01872/29-35 HPRD_02887/204-210 HPRD_02888/127-133 HPRD_04530/121-127
RxRSRxR	8.68E-42	5	7	HPRD_01617/241-248 HPRD_01872/30-37 HPRD_02887/203-210 HPRD_02888/124-131 HPRD_04530/152-159
RxxSRSR	4.63E-41	5	7	HPRD_01617/253-260 HPRD_01872/26-33 HPRD_02887/203-210 HPRD_02888/124-131 HPRD_04530/118-125
>HPRD_04015 Pfam:2-Hacid_dh Pfam:2-Hacid_dh_C				
PxDLS	3.08E-53	7	15	HPRD_00883/1081-1086 HPRD_01310/583-588 HPRD_03927/439-444 HPRD_04023/23-28 HPRD_04318/33-38 HPRD_04737/828-833 HPRD_04990/489-494
PLDLS	6.73E-50	4	15	HPRD_00883/1081-1086 HPRD_01310/583-588 HPRD_04023/23-28 HPRD_04990/489-494
SPxN	1.57E-40	7	15	HPRD_00218/631-635 HPRD_00883/2656-2660 HPRD_01310/859-863 HPRD_03143/345-349 HPRD_03927/355-359 HPRD_04318/388-392 HPRD_04990/567-571 HPRD_05521/346-350 HPRD_05874/345-349
>HPRD_04070 HLH PAS Pfam:DUF1518				
PxxSP	9.32E-53	12	39	HPRD_00179/362-367 HPRD_00524/684-689 HPRD_00589/114-119 HPRD_00744/10-15 HPRD_01302/239-244 HPRD_01369/73-78 HPRD_01578/95-100 HPRD_01702/199-204 HPRD_02437/512-517 HPRD_02511/25-30 HPRD_02534/1908-1913 HPRD_02596/209-214 HPRD_02612/462-467 HPRD_02618/103-108 HPRD_03390/101-106 HPRD_03432/213-218 HPRD_04078/251-256 HPRD_05155/269-274 HPRD_06769/445-450 HPRD_06780/36-41 HPRD_07077/290-295
SPxxxS	1.19E-50	15	39	HPRD_00179/373-379 HPRD_00679/403-409 HPRD_00744/94-100 HPRD_01275/20-26 HPRD_01577/90-96 HPRD_01578/81-87 HPRD_02475/287-293 HPRD_02534/744-750 HPRD_02596/591-597 HPRD_02612/465-471 HPRD_02618/415-421 HPRD_02788/310-316 HPRD_02798/17-23 HPRD_03288/111-117 HPRD_03390/512-518 HPRD_04078/1094-1100 HPRD_04274/56-62 HPRD_04517/499-505 HPRD_05140/476-482 HPRD_05155/312-318 HPRD_06769/455-461 HPRD_07077/19-25
SPxxxxS	3.76E-49	18	39	HPRD_00026/726-733 HPRD_00179/389-396 HPRD_00583/33-40 HPRD_00679/403-410 HPRD_00744/133-140 HPRD_01238/892-899 HPRD_01275/31-38 HPRD_01302/242-249 HPRD_01346/218-225 HPRD_01369/5-12 HPRD_01577/20-27 HPRD_01578/130-137 HPRD_02437/425-432 HPRD_02475/279-286 HPRD_02534/1916-1923 HPRD_02596/726-733 HPRD_02612/64-71 HPRD_02618/302-309 HPRD_02788/310-317 HPRD_03288/7-14 HPRD_03390/5-12 HPRD_03432/45-52 HPRD_04078/19-26 HPRD_04274/80-87 HPRD_04517/580-587 HPRD_05155/312-319 HPRD_06769/444-451 HPRD_06780/269-276 HPRD_07077/101-108
>HPRD_04078 BROMO COIL Pfam:DUF906 Pfam:KIX ZnF_TAZ ZnF_ZZ				
PxSP	1.84E-57	22	56	HPRD_00026/724-728 HPRD_00549/23-27 HPRD_00744/234-238 HPRD_00918/136-140 HPRD_01075/197-201 HPRD_01369/9-13 HPRD_01518/12-16 HPRD_02622/280-284 HPRD_02668/219-223 HPRD_02726/452-456 HPRD_02730/70-74 HPRD_02807/405-409 HPRD_03247/390-394 HPRD_03330/536-540 HPRD_03337/125-129 HPRD_03356/184-188 HPRD_03374/8-12 HPRD_03428/271-275 HPRD_03991/276-280 HPRD_04070/348-352 HPRD_04517/512-516 HPRD_05599/1026-1030 HPRD_05665/156-160 HPRD_07567/295-299 HPRD_10144/988-992

QxPxxxxP	3.64E-56	17	56	HPRD_00128/35-43 HPRD_00218/974-982 HPRD_00549/233-241 HPRD_00744/69-77 HPRD_00918/367-375 HPRD_01166/305-313 HPRD_01241/493-501 HPRD_02559/252-260 HPRD_02622/58-66 HPRD_02726/237-245 HPRD_02807/429-437 HPRD_02896/206-214 HPRD_03251/50-58 HPRD_03302/718-726 HPRD_03330/377-385 HPRD_03356/216-224 HPRD_03428/78-86 HPRD_03991/515-523 HPRD_05599/234-242 HPRD_05665/75-83
SxxxSP	1.33E-55	20	56	HPRD_00218/627-633 HPRD_00549/21-27 HPRD_00589/101-107 HPRD_00744/90-96 HPRD_01166/196-202 HPRD_01369/72-78 HPRD_01518/65-71 HPRD_01801/226-232 HPRD_02146/62-68 HPRD_02668/150-156 HPRD_02679/45-51 HPRD_02726/450-456 HPRD_02730/179-185 HPRD_02807/160-166 HPRD_03316/100-106 HPRD_03330/131-137 HPRD_03356/190-196 HPRD_03374/6-12 HPRD_04070/439-445 HPRD_04185/265-271 HPRD_04517/584-590 HPRD_05599/364-370 HPRD_05665/604-610 HPRD_07567/293-299 HPRD_09185/214-220 HPRD_10144/476-482
>HPRD_04199 GuKc PDZ SH3				
PPxxP	2.22E-56	20	52	HPRD_00138/1146-1151 HPRD_00159/85-90 HPRD_00160/87-92 HPRD_00181/284-289 HPRD_00697/1112-1117 HPRD_00699/929-934 HPRD_01498/611-616 HPRD_02164/461-466 HPRD_02177/358-363 HPRD_02275/85-90 HPRD_02336/89-94 HPRD_02509/4612-4617 HPRD_02533/502-507 HPRD_02549/369-374 HPRD_03131/712-717 HPRD_04062/1402-1407 HPRD_04095/290-295 HPRD_04479/299-304 HPRD_04558/43-48 HPRD_04663/319-324 HPRD_05085/725-730 HPRD_05413/927-932 HPRD_05818/1174-1179 HPRD_06090/1116-1121 HPRD_07257/717-722 HPRD_09258/533-538 HPRD_09260/541-546
PCxxxxxG	4.04E-56	7	52	HPRD_00138/2480-2488 HPRD_00699/933-941 HPRD_01226/1247-1255 HPRD_01281/193-201 HPRD_02509/661-669 HPRD_02549/37-45 HPRD_04062/41-49 HPRD_04558/579-587 HPRD_05255/127-135 HPRD_06459/489-497 HPRD_09258/348-356 HPRD_09260/337-345
SxSxxxxS	4.68E-56	21	52	HPRD_00160/1223-1231 HPRD_00181/46-54 HPRD_00406/297-305 HPRD_00693/886-894 HPRD_00697/538-546 HPRD_00698/537-545 HPRD_00699/1015-1023 HPRD_01226/851-859 HPRD_01285/76-84 HPRD_01443/446-454 HPRD_01444/601-609 HPRD_01478/317-325 HPRD_01498/559-567 HPRD_01638/9-17 HPRD_02164/574-582 HPRD_02177/487-495 HPRD_02509/1815-1823 HPRD_02533/280-288 HPRD_02549/1195-1203 HPRD_02614/14-22 HPRD_03131/391-399 HPRD_03732/337-345 HPRD_04062/321-329 HPRD_04095/1110-1118 HPRD_04220/43-51 HPRD_04479/281-289 HPRD_04663/522-530 HPRD_04748/587-595 HPRD_05413/1163-1171 HPRD_05818/395-403 HPRD_06090/659-667 HPRD_06459/296-304 HPRD_09258/162-170 HPRD_09260/151-159
>HPRD_04232 WD40				
SxxxxTxQ	2.14E-43	6	12	HPRD_00218/1388-1396 HPRD_01796/420-428 HPRD_02534/1933-1941 HPRD_03143/345-353 HPRD_04647/290-298 HPRD_04653/187-195 HPRD_05521/346-354 HPRD_10230/832-840
PxxQxxxI	6.27E-43	4	12	HPRD_01796/168-176 HPRD_02534/687-695 HPRD_03143/376-384 HPRD_04647/243-251 HPRD_04653/99-107 HPRD_05521/377-385
QxQxxP	1.47E-40	5	12	HPRD_01796/171-177 HPRD_02534/101-107 HPRD_03143/379-385 HPRD_03483/168-174 HPRD_04633/691-697 HPRD_05521/380-386 HPRD_10230/3276-3282
>HPRD_04241 DWA DWB				
ExxExSxR	6.55E-41	4	17	HPRD_05994/949-957 HPRD_06090/508-516 HPRD_06901/400-408 HPRD_06902/406-414 HPRD_09318/156-164
PxxGH	3.64E-38	6	17	HPRD_04011/407-412 HPRD_05994/1116-1121 HPRD_06090/1310-1315 HPRD_06901/387-392 HPRD_06902/393-398 HPRD_09318/780-785 HPRD_09424/47-52
>HPRD_04248 SH3				
PxKPP	2.62E-54	6	24	HPRD_01381/307-312 HPRD_02513/333-338 HPRD_02627/284-289 HPRD_02859/894-899 HPRD_02900/934-939 HPRD_03131/855-860 HPRD_05026/337-342 HPRD_05283/209-214
PxKP	1.23E-45	8	24	HPRD_00796/245-249 HPRD_01080/228-232 HPRD_01301/230-234 HPRD_01381/307-311 HPRD_01552/1090-1094 HPRD_02019/429-433 HPRD_02513/333-337 HPRD_02627/61-65 HPRD_02859/733-737 HPRD_02900/934-938 HPRD_03131/713-717 HPRD_03942/863-867 HPRD_05026/240-244 HPRD_05283/209-213 HPRD_09524/430-434
PKPxxP	1.74E-45	4	24	HPRD_00674/275-281 HPRD_01301/228-234 HPRD_01381/86-92 HPRD_02627/285-291 HPRD_02859/734-740 HPRD_03131/714-720 HPRD_05026/335-341
>HPRD_04257				
GxxxRGRG	9.47E-66	8	13	HPRD_00617/36-44 HPRD_00660/237-245 HPRD_02834/251-259 HPRD_03655/691-699 HPRD_03926/284-292 HPRD_04185/701-709 HPRD_04420/644-652 HPRD_08375/105-113
GxxxRGxG	1.46E-61	9	13	HPRD_00617/3-11 HPRD_00660/237-245 HPRD_02834/251-259 HPRD_03655/691-699 HPRD_03926/284-292 HPRD_04185/701-709 HPRD_04386/1242-1250 HPRD_04420/644-652 HPRD_08375/105-113
RGRG	9.54E-59	8	13	HPRD_00617/33-37 HPRD_00660/215-219 HPRD_02834/255-259 HPRD_03655/695-699 HPRD_03926/281-285 HPRD_04185/705-709 HPRD_04420/639-643 HPRD_08375/107-111
>HPRD_04380 DWA DWB				
PxxxPxxG	0.00E+00	14	47	HPRD_00304/285-293 HPRD_00564/66-74 HPRD_00645/121-129 HPRD_01271/167-175 HPRD_01303/216-224 HPRD_01310/318-326 HPRD_01313/223-231 HPRD_01319/211-219 HPRD_01801/53-61 HPRD_01827/1386-1394 HPRD_02070/43-51 HPRD_02439/292-300 HPRD_02806/5-13 HPRD_02995/291-299 HPRD_03139/619-627 HPRD_04061/131-139 HPRD_04689/158-166 HPRD_05780/917-925 HPRD_06090/1306-1314 HPRD_09318/481-489
SxxxSxxS	5.60E-68	25	47	HPRD_00312/564-572 HPRD_00443/437-445 HPRD_00645/32-40 HPRD_00679/389-397 HPRD_01113/44-52 HPRD_01271/493-501 HPRD_01310/44-52 HPRD_01313/71-79 HPRD_01319/107-115 HPRD_01798/277-285 HPRD_01801/220-228 HPRD_01818/202-210 HPRD_01827/2198-2206 HPRD_02070/256-264 HPRD_02437/157-165 HPRD_02439/555-563 HPRD_02554/1745-1753 HPRD_02566/9-17 HPRD_02806/306-314 HPRD_03100/386-394 HPRD_03139/305-313 HPRD_03221/459-467 HPRD_04061/310-318 HPRD_04082/138-146 HPRD_04340/28-36 HPRD_04689/163-171 HPRD_04819/320-328 HPRD_05780/319-327 HPRD_06090/674-682 HPRD_09273/142-150 HPRD_09318/134-142
PxxxPxP	7.73E-68	20	47	HPRD_00304/285-292 HPRD_00312/960-967 HPRD_00443/209-216 HPRD_00564/64-71 HPRD_00645/11-18 HPRD_01271/76-83 HPRD_01310/503-510 HPRD_01313/188-195 HPRD_01319/126-133 HPRD_01801/8-15 HPRD_01818/230-237 HPRD_01827/2216-2223 HPRD_01919/127-134 HPRD_02070/71-78 HPRD_02437/145-152 HPRD_02439/140-147 HPRD_02554/2795-2802 HPRD_02566/247-254 HPRD_02806/5-12 HPRD_02995/291-298 HPRD_03139/287-294 HPRD_03672/341-348 HPRD_04061/131-138 HPRD_04340/218-225 HPRD_04689/152-159 HPRD_04819/692-699 HPRD_05780/917-924 HPRD_09273/245-252 HPRD_09318/628-635
>HPRD_04582 PTB				
FxNPxY	6.81E-58	4	9	HPRD_00138/4501-4507 HPRD_01895/831-837 HPRD_02509/4520-4526 HPRD_04002/860-866 HPRD_06091/822-828 HPRD_09903/634-640
NPxY	7.39E-55	5	9	HPRD_00100/758-762 HPRD_00102/639-643 HPRD_00103/751-755 HPRD_00138/844-848 HPRD_01895/833-837 HPRD_02509/2854-2858 HPRD_04002/862-866 HPRD_06091/824-828 HPRD_09903/636-640
FxxPxY	4.99E-38	4	9	HPRD_00138/4501-4507 HPRD_01895/831-837 HPRD_02509/330-336 HPRD_04002/860-866 HPRD_06091/822-828 HPRD_09903/634-640

>HPRD_04720				
SRSRxR	3.29E-43	5	5	HPRD_01617/256-262 HPRD_01872/29-35 HPRD_02887/204-210 HPRD_02888/127-133 HPRD_04530/121-127
RxRSRxR	4.15E-43	5	5	HPRD_01617/241-248 HPRD_01872/30-37 HPRD_02887/203-210 HPRD_02888/124-131 HPRD_04530/152-159
RxxSRSR	2.21E-42	5	5	HPRD_01617/253-260 HPRD_01872/26-33 HPRD_02887/203-210 HPRD_02888/124-131 HPRD_04530/118-125
>HPRD_05125 BAR SH3				
PxxxPxRP	3.80E-39	5	7	HPRD_01320/543-551 HPRD_02301/417-425 HPRD_03851/785-793 HPRD_04091/782-790 HPRD_05373/470-478
>HPRD_05131 CHROMO ChSh				
EExExE	1.88E-42	6	14	HPRD_01038/207-213 HPRD_01071/13-19 HPRD_03148/601-607 HPRD_04459/662-668 HPRD_05130/22-28 HPRD_05149/14-20 HPRD_05610/522-528 HPRD_05944/505-511
>HPRD_05378 SH2 SH3				
PPxP	4.68E-39	12	18	HPRD_01044/268-272 HPRD_01423/36-40 HPRD_01681/1150-1154 HPRD_02834/288-292 HPRD_02859/733-737 HPRD_03240/1799-1803 HPRD_03842/259-263 HPRD_03972/574-578 HPRD_03995/11-15 HPRD_04228/80-84 HPRD_04336/180-184 HPRD_04819/22-26 HPRD_08923/180-184
>HPRD_05457 14_3_3				
RxxSxP	6.33E-51	10	25	HPRD_00305/102-108 HPRD_00307/133-139 HPRD_01069/1133-1139 HPRD_01265/255-261 HPRD_01821/57-63 HPRD_01857/561-567 HPRD_02409/308-314 HPRD_02533/718-724 HPRD_02878/428-434 HPRD_03965/246-252 HPRD_04342/911-917 HPRD_04409/71-77 HPRD_05610/463-469 HPRD_06340/332-338 HPRD_07362/309-315 HPRD_09246/494-500 HPRD_09580/85-91
SxPxS	1.28E-45	15	25	HPRD_00305/281-286 HPRD_00307/343-348 HPRD_00536/1386-1391 HPRD_00943/2-7 HPRD_01069/238-243 HPRD_01265/242-247 HPRD_01819/38-43 HPRD_01857/553-558 HPRD_02409/479-484 HPRD_02533/501-506 HPRD_02878/614-619 HPRD_03720/569-574 HPRD_03965/129-134 HPRD_04342/914-919 HPRD_05610/214-219 HPRD_05813/253-258 HPRD_06340/193-198 HPRD_09246/224-229 HPRD_09580/88-93
RxRxxS	1.49E-45	10	25	HPRD_00536/1463-1469 HPRD_00932/959-965 HPRD_00943/264-270 HPRD_01069/325-331 HPRD_01265/253-259 HPRD_02409/147-153 HPRD_02878/300-306 HPRD_03720/832-838 HPRD_03965/160-166 HPRD_04409/69-75 HPRD_07362/475-481 HPRD_09246/488-494
>HPRD_05521 COIL Pfam:Hist_deacetyl				
HxRxxxxE	4.39E-39	6	32	HPRD_00218/838-846 HPRD_01796/645-653 HPRD_03483/640-648 HPRD_03742/466-474 HPRD_05465/112-120 HPRD_05568/326-334 HPRD_05874/74-82 HPRD_07185/63-71 HPRD_09071/1829-1837 HPRD_10230/122-130
SxSD	2.61E-38	12	32	HPRD_00218/386-390 HPRD_00532/140-144 HPRD_00536/706-710 HPRD_01575/274-278 HPRD_02487/154-158 HPRD_03742/439-443 HPRD_04232/194-198 HPRD_04340/478-482 HPRD_04647/290-294 HPRD_05568/350-354 HPRD_05874/237-241 HPRD_09426/45-49 HPRD_10230/278-282
PxxxLxxS	2.83E-38	11	32	HPRD_00218/370-378 HPRD_00277/362-370 HPRD_00536/1517-1525 HPRD_01575/1124-1132 HPRD_02679/32-40 HPRD_03143/338-346 HPRD_03742/316-324 HPRD_04612/235-243 HPRD_04647/347-355 HPRD_04803/447-455 HPRD_05874/196-204 HPRD_09343/202-210 HPRD_09426/40-48 HPRD_10230/317-325
>HPRD_05610 COIL Pfam:Hist_deacetyl				
RPxSxG	2.34E-41	4	14	HPRD_01310/619-625 HPRD_02807/197-203 HPRD_02809/188-194 HPRD_02810/197-203 HPRD_02910/2452-2458 HPRD_02911/1746-1752
KxExxSP	2.87E-38	4	14	HPRD_01310/532-539 HPRD_02807/402-409 HPRD_02809/390-397 HPRD_02810/438-445 HPRD_02910/143-150 HPRD_02911/1105-1112
EPxSP	2.92E-38	5	14	HPRD_01310/534-539 HPRD_02807/404-409 HPRD_02809/392-397 HPRD_02810/440-445 HPRD_02910/145-150 HPRD_02911/2147-2152 HPRD_05944/18-23
>HPRD_06039 S_TKc				
TPPP	6.48E-39	4	14	HPRD_02534/855-859 HPRD_03221/219-223 HPRD_03356/221-225 HPRD_03587/166-170 HPRD_04469/216-220 HPRD_09367/98-102
>HPRD_06780 BROMO Pfam:Acetyltransf_1 Pfam:PCAF_N				
SPxxxxxS	7.33E-44	12	22	HPRD_00295/466-474 HPRD_00307/41-49 HPRD_00744/204-212 HPRD_00800/351-359 HPRD_00918/133-141 HPRD_01310/321-329 HPRD_01827/876-884 HPRD_02554/3283-3291 HPRD_02607/663-671 HPRD_03374/10-18 HPRD_03927/217-225 HPRD_04070/21-29 HPRD_05620/279-287
SxPxT	6.06E-41	12	22	HPRD_00295/1044-1049 HPRD_00307/48-53 HPRD_00744/22-27 HPRD_00918/350-355 HPRD_00961/142-147 HPRD_01272/153-158 HPRD_01310/255-260 HPRD_01827/2537-2542 HPRD_02554/561-566 HPRD_02607/625-630 HPRD_03927/408-413 HPRD_04070/1421-1426 HPRD_05002/97-102
SxxxxSS	1.96E-40	16	22	HPRD_00295/456-463 HPRD_00307/80-87 HPRD_00744/83-90 HPRD_00800/544-551 HPRD_00918/60-67 HPRD_00920/472-479 HPRD_00961/97-104 HPRD_00962/315-322 HPRD_01272/153-160 HPRD_01310/385-392 HPRD_01827/2283-2290 HPRD_02554/444-451 HPRD_03584/273-280 HPRD_03927/402-409 HPRD_04070/1-8 HPRD_05002/30-37 HPRD_05620/273-280
>HPRD_07521 HOLI ZnF_C4				
PxSxKxK	2.18E-39	4	9	HPRD_04070/13-20 HPRD_05055/1215-1222 HPRD_05141/433-440 HPRD_05155/772-779 HPRD_09066/733-740
>HPRD_08916 Pfam:RNA_pol_Rpb1_4 Pfam:RNA_pol_Rpb1_5 Pfam:RNA_pol_Rpb1_6 Pfam:RNA_pol_Rpb1_7 Pfam:RNA_pol_Rpb1_R RPOLA_N				
ExxFxxH	4.34E-40	4	20	HPRD_00218/999-1006 HPRD_02483/1021-1028 HPRD_02534/1629-1636 HPRD_03435/88-95 HPRD_03437/60-67 HPRD_04459/1055-1062
NxxxxxFC	7.35E-40	4	20	HPRD_00218/297-305 HPRD_02483/1061-1069 HPRD_02534/1835-1843 HPRD_03254/137-145 HPRD_04459/1095-1103 HPRD_09156/12-20
ExPDD	7.98E-40	4	20	HPRD_00218/1147-1152 HPRD_02483/1227-1232 HPRD_04382/176-181 HPRD_04422/361-366 HPRD_04459/1294-1299
>HPRD_08968 EFh EH UIM				

TNPF	1.02E-41	4	12	HPRD_02918/432-436 HPRD_03542/5-9 HPRD_04767/635-639 HPRD_06270/491-495
>HPRD_09045 UBCc				
IKxE	3.90E-41	9	29	HPRD_00535/101-105 HPRD_00679/291-295 HPRD_01138/287-291 HPRD_01260/13-17 HPRD_01302/252-256 HPRD_01701/121-125 HPRD_01810/501-505 HPRD_02398/86-90 HPRD_02575/142-146 HPRD_02777/701-705 HPRD_03111/333-337 HPRD_03742/569-573 HPRD_04078/1022-1026 HPRD_06163/71-75 HPRD_09071/625-629
PxSP	3.38E-38	10	29	HPRD_00443/263-267 HPRD_00683/207-211 HPRD_00688/223-227 HPRD_01138/177-181 HPRD_01302/240-244 HPRD_01701/60-64 HPRD_02726/452-456 HPRD_02777/724-728 HPRD_03111/125-129 HPRD_03742/77-81 HPRD_04078/2060-2064 HPRD_04141/252-256 HPRD_04209/317-321 HPRD_06039/1185-1189 HPRD_09071/710-714
>HPRD_06224 RGS				
CxxSxExK	7.64E-42	4	4	HPRD_00756/2-10 HPRD_00764/2-10 HPRD_00765/2-10 HPRD_02998/8-16
**V. Domain sets**				
**>Domains and putative binding motifs**	**P_{cons}**	**Motif count**	**Total**	**Proteins with corresponding motif (identifiers/coordinates)**
***S.cerevisiae* domain set**				
>SH3*				
PxxPPxP	1.91E-20	6	38	YBL007C/621-628 YER144C/415-422 YGR268C/26-33 YNL152W/328-335 YNR006W/523-530 YOR181W/322-329
***D.melanogaster* domain set**				
>2-Hacid_dh				
PxDLS	8.1E-23	9	22	CG3758/258-263 CG3956/40-45 CG3340/413-418 CG4717/330-335 CG13651/388-393 CG9653/174-179 CG10318/100-105 CG7383/313-318 CG4761/498-503
PxDxS	1.9E-21	12	22	CG3758/258-263 CG3956/40-45 CG3340/413-418 CG4717/330-335 CG3850/334-339 CG13651/388-393 CG9653/174-179 CG8524/334-339 CG5460/804-809 CG10318/100-105 CG7383/313-318 CG4761/498-503
PxDLSxxS	2.2E-19	5	22	CG3340/463-471 CG13651/388-396 CG9653/174-182 CG10318/100-108 CG7383/313-321
>Dynein_light*				
TQT	1.4E-15	8	18	CG3183/85-88 CG8262/163-166 CG18568/38-41 CG14962/257-260 CG3429/292-295 CG17601/31-34 CG32130/217-220 CG13074/53-56 CG10573/399-402
>FBOX				
GxxRGRG	1.1E-15	3	12	CG5920/22-29 CG3496/1838-1845 CG6745/1-8
>G_patch				
RSRxR	7.1E-29	8	32	CG9998/15-20 CG4602/285-290 CG7818/10-15 CG5808/452-457 CG5792/165-170 CG3019/839-844 CG6340/134-139 CG3162/14-19 CG11274/140-145 CG6843/339-344
RxRxxSRS	6.3E-25	6	32	CG9998/11-19 CG4602/283-291 CG3019/896-904 CG6340/136-144 CG3162/14-22 CG11274/136-144 CG6843/341-349
>IPP-2*				
PGK	2.3E-15	6	8	CG5650/21-24 CG12396/658-661 CG1553/233-236 CG32685/195-198 CG31291/636-639 CG6477/299-302
KKxVxxxD	5.1E-15	3	8	CG1553/733-741 CG32685/1829-1837 CG9238/102-110
>SH2/SH3*				
PPR	1.6E-18	13	44	CG2079/347-350 CG17256/499-502 CG4563/20-23 CG2225/412-415 CG1363/319-322 CG6203/493-496 CG31045/202-205 CG30052/144-147 CG17050/12-15 CG9181/212-215 CG4821/332-335 CG9695/782-785 CG31534/626-629
PxPP	8.2E-18	18	44	CG2079/263-267 CG17256/497-501 CG2225/422-426 CG1363/324-328 CG14632/201-205 CG12724/53-57 CG31342/188-192 CG9759/99-103 CG31045/75-79 CG30052/59-63 CG1212/144-148 CG9181/210-214 CG13503/3-7 CG13289/214-218 CG4821/269-273 CG9695/329-333 CG31534/137-141 CG4285/244-248 CG8505/108-112
PPxxP	1.2E-17	17	44	CG2079/406-411 CG17256/496-501 CG13964/9-14 CG2225/462-467 CG1363/570-575 CG14632/131-136 CG31342/190-195 CG9759/99-104 CG31045/1-6 CG30052/59-64 CG17050/1-6 CG9181/208-213 CG13503/4-9 CG13289/163-168 CG4821/271-276 CG9695/626-631 CG31534/139-144 CG4285/744-749 CG8505/110-115
***C.elegans* domain set**				
>SH2/SH3*				
PxLPPR	2.78E-23	5	26	C02F4.1/1759-1765 C11E4.6/989-995 F46F6.1a/615-621 M02A10.3a/556-562 T28F12.3/1309-1315
PPLPPR	1.12E-21	5	26	C02F4.1/1759-1765 C11E4.6/989-995 F46F6.1a/615-621 M02A10.3a/556-562 T28F12.3/1309-1315
PLPPR	4.77E-20	5	26	C02F4.1/1760-1765 C11E4.6/990-995 F46F6.1a/616-621 M02A10.3a/557-562 T28F12.3/1310-1315
>SH3*				
PxxPPR	6.13E-26	9	33	C02F4.1/1759-1765 C11E4.6/989-995 C18H9.7/550-556 F45E10.1a/489-495 F46F6.1a/615-621 F56C9.1/314-320 M02A10.3a/469-475 T28F12.3/1309-1315 W04D2.1a/900-906

PPxxPR	5.12E-23	9	33	C02F4.1/1759-1765 C11E4.6/989-995 C18H9.7/550-556 F46F6.1a/557-563 M02A10.3a/556-562 T17H7.4a/531-537 T20F5.6/400-406 T28F12.3/1207-1213 ZK418.4/98-104
PxLPPR	9.98E-23	5	33	C02F4.1/1759-1765 C11E4.6/989-995 F46F6.1a/615-621 M02A10.3a/556-562 T28F12.3/1309-1315
>SH3_1*				
PxxPPR	6.13E-26	9	33	C02F4.1/1759-1765 C11E4.6/989-995 C18H9.7/550-556 F45E10.1a/489-495 F46F6.1a/615-621 F56C9.1/314-320 M02A10.3a/469-475 T28F12.3/1309-1315 W04D2.1a/900-906
PPxxPR	5.12E-23	9	33	C02F4.1/1759-1765 C11E4.6/989-995 C18H9.7/550-556 F46F6.1a/557-563 M02A10.3a/556-562 T17H7.4a/531-537 T20F5.6/400-406 T28F12.3/1207-1213 ZK418.4/98-104
PxLPPR	9.98E-23	5	33	C02F4.1/1759-1765 C11E4.6/989-995 F46F6.1a/615-621 M02A10.3a/556-562 T28F12.3/1309-1315
>SH3_2*				
PxxPPR	4.5E-26	9	32	C02F4.1/1759-1765 C11E4.6/989-995 C18H9.7/550-556 F45E10.1a/489-495 F46F6.1a/615-621 F56C9.1/314-320 M02A10.3a/469-475 T28F12.3/1309-1315 W04D2.1a/900-906
PPxxPR	3.8E-23	9	32	C02F4.1/1759-1765 C11E4.6/989-995 C18H9.7/550-556 F46F6.1a/557-563 M02A10.3a/556-562 T17H7.4a/531-537 T20F5.6/400-406 T28F12.3/1207-1213 ZK418.4/98-104
PxLPPR	8.5E-23	5	32	C02F4.1/1759-1765 C11E4.6/989-995 F46F6.1a/615-621 M02A10.3a/556-562 T28F12.3/1309-1315
PxxPxR	2.6E-22	13	32	C02F4.1/1759-1765 C11E4.6/727-733 C18H9.7/550-556 F42H10.7/375-381 F45E10.1a/489-495 F46F6.1a/549-555 F56C9.1/314-320 M02A10.3a/469-475 T17H7.4a/532-538 T20F5.6/398-404 T28F12.3/1284-1290 W04D2.1a/900-906 ZK418.4/201-207
***H.sapiens* domain set**				
>14_3_3				
SxPxSP	0.00E+00	14	91	HPRD_00739/836-842 HPRD_01069/932-938 HPRD_01142/514-520 HPRD_01264/145-151 HPRD_01850/659-665 HPRD_02533/501-507 HPRD_02878/983-989 HPRD_03470/436-442 HPRD_03904/1028-1034 HPRD_03927/802-808 HPRD_05298/455-461 HPRD_05610/297-303 HPRD_05622/509-515 HPRD_05813/253-259 HPRD_06340/193-199 HPRD_09246/316-322 HPRD_09436/879-885
>2-Hacid_dh				
PxDLS	1.32E-64	8	20	HPRD_00883/1081-1086 HPRD_01310/583-588 HPRD_01798/733-738 HPRD_02238/85-90 HPRD_03927/439-444 HPRD_04023/23-28 HPRD_04318/33-38 HPRD_04737/828-833 HPRD_04990/489-494
PLDLS	1.06E-61	5	20	HPRD_00883/1081-1086 HPRD_01310/583-588 HPRD_01798/733-738 HPRD_04023/23-28 HPRD_04990/489-494
>2-Hacid_dh_C				
PxDLS	1.32E-64	8	20	HPRD_00883/1081-1086 HPRD_01310/583-588 HPRD_01798/733-738 HPRD_02238/85-90 HPRD_03927/439-444 HPRD_04023/23-28 HPRD_04318/33-38 HPRD_04737/828-833 HPRD_04990/489-494
PLDLS	1.06E-61	5	20	HPRD_00883/1081-1086 HPRD_01310/583-588 HPRD_01798/733-738 HPRD_04023/23-28 HPRD_04990/489-494
>Adaptin_N				
LxDL	4.40E-45	13	45	HPRD_00350/283-287 HPRD_00569/6-10 HPRD_01500/302-306 HPRD_01686/480-484 HPRD_01822/176-180 HPRD_02358/331-335 HPRD_02541/164-168 HPRD_02549/2299-2303 HPRD_02739/96-100 HPRD_02893/76-80 HPRD_02981/317-321 HPRD_03150/389-393 HPRD_03461/268-272 HPRD_04637/356-360 HPRD_05074/749-753 HPRD_06270/231-235 HPRD_06291/613-617 HPRD_08968/47-51 HPRD_09061/176-180 HPRD_09135/170-174 HPRD_09256/232-236 HPRD_09887/752-756
>Cyclin_C				
KRK	5.03E-60	15	42	HPRD_00024/184-187 HPRD_00093/124-127 HPRD_00218/502-505 HPRD_00298/160-163 HPRD_01576/934-937 HPRD_02436/281-284 HPRD_02437/845-848 HPRD_02605/162-165 HPRD_03247/180-183 HPRD_03435/323-326 HPRD_03570/16-19 HPRD_04070/17-20 HPRD_04078/333-336 HPRD_04459/1331-1334 HPRD_05258/303-306 HPRD_05888/168-171 HPRD_06780/427-430 HPRD_07185/24-27 HPRD_08909/103-106
>PROF				
PxPxPP	4.33E-42	5	9	HPRD_01164/1348-1354 HPRD_01381/86-92 HPRD_03415/171-177 HPRD_03851/797-803 HPRD_03852/782-788 HPRD_04893/186-192 HPRD_05434/277-283 HPRD_05448/276-282
PxPxxPxP	5.76E-40	5	9	HPRD_01164/1348-1356 HPRD_01381/86-94 HPRD_03415/170-178 HPRD_03851/822-830 HPRD_03852/803-811 HPRD_04893/189-197 HPRD_05434/321-329 HPRD_05448/286-294
>STAT_alpha				
WSxWS	9.62E-71	4	80	HPRD_00587/232-237 HPRD_00896/219-224 HPRD_01000/302-307 HPRD_02391/236-241 HPRD_02824/309-314 HPRD_03001/318-323 HPRD_03363/221-226 HPRD_03383/304-309
>STAT_bind				
WSxWS	9.62E-71	4	80	HPRD_00587/232-237 HPRD_00896/219-224 HPRD_01000/302-307 HPRD_02391/236-241 HPRD_02824/309-314 HPRD_03001/318-323 HPRD_03363/221-226 HPRD_03383/304-309
>TUDOR				
GxxRG	1.71E-57	11	44	HPRD_01655/128-133 HPRD_01806/105-110 HPRD_02605/413-418 HPRD_03039/93-98 HPRD_03040/96-101 HPRD_03302/463-468 HPRD_03342/356-361 HPRD_04386/1170-1175 HPRD_04646/28-33 HPRD_05130/115-120 HPRD_05131/115-120 HPRD_06228/202-207 HPRD_06284/86-91 HPRD_06734/199-204 HPRD_07569/4-9 HPRD_07600/19-24
RGRG	5.23E-56	8	44	HPRD_01806/108-112 HPRD_02605/412-416 HPRD_03039/109-113 HPRD_03040/97-101 HPRD_06109/257-261 HPRD_06228/446-450 HPRD_06284/87-91 HPRD_06734/443-447 HPRD_07569/212-216
>WW				

PPxxP	0.00E+00	25	85	HPRD_00102/211-216 HPRD_00103/596-601 HPRD_00138/1146-1151 HPRD_00181/284-289 HPRD_00304/400-405 HPRD_00546/827-832 HPRD_00610/44-49 HPRD_00932/632-637 HPRD_01142/434-439 HPRD_01302/239-244 HPRD_01477/307-312 HPRD_01504/532-537 HPRD_01827/1227-1232 HPRD_02301/341-346 HPRD_02490/83-88 HPRD_02491/92-97 HPRD_02509/4612-4617 HPRD_02566/247-252 HPRD_02734/238-243 HPRD_03009/73-78 HPRD_03260/256-261 HPRD_03333/88-93 HPRD_03356/228-233 HPRD_03421/208-213 HPRD_03432/211-216 HPRD_03587/333-338 HPRD_03602/63-68 HPRD_03655/65-70 HPRD_03920/51-56 HPRD_04062/1402-1407 HPRD_04191/9-14 HPRD_04264/74-79 HPRD_04411/189-194 HPRD_05085/725-730 HPRD_05136/504-509 HPRD_05457/163-168 HPRD_05946/383-388 HPRD_06311/160-165 HPRD_10108/155-160
>ZnF_C3H1				
SRSRxR	2.02E-62	6	10	HPRD_01871/194-200 HPRD_01872/29-35 HPRD_02068/419-425 HPRD_02887/204-210 HPRD_02888/127-133 HPRD_09185/222-228
SRSRxRxR	2.62E-62	6	10	HPRD_01871/194-202 HPRD_01872/29-37 HPRD_02068/419-427 HPRD_02887/204-212 HPRD_02888/127-135 HPRD_09185/222-230
>zf-TRAF				
PxQE	5.17E-46	12	74	HPRD_00178/228-232 HPRD_01074/560-564 HPRD_01504/188-192 HPRD_01723/245-249 HPRD_01857/238-242 HPRD_02230/22-26 HPRD_02636/261-265 HPRD_02988/401-405 HPRD_03319/368-372 HPRD_04491/182-186 HPRD_04609/568-572 HPRD_05230/168-172 HPRD_05365/239-243 HPRD_05374/680-684 HPRD_09442/43-47 HPRD_09571/376-380
PxxExG	1.24E-44	15	74	HPRD_00144/146-152 HPRD_01074/336-342 HPRD_01723/236-242 HPRD_01863/461-467 HPRD_02060/583-589 HPRD_02235/613-619 HPRD_02988/311-317 HPRD_03028/16-22 HPRD_03319/195-201 HPRD_03767/176-182 HPRD_03904/879-885 HPRD_04011/227-233 HPRD_04012/470-476 HPRD_04609/606-612 HPRD_04625/174-180 HPRD_04879/213-219 HPRD_05230/198-204 HPRD_05365/284-290 HPRD_05801/112-118 HPRD_06427/257-263 HPRD_09442/1667-1673
